# Supplementary material for: Effects of education and age on the experience of youth violence in a very low-resource setting: a fixed-effects analysis in rural Burkina Faso
Source: BMJ Open. 2023 Oct 18;13(10):e071104. doi: 10.1136/bmjopen-2022-071104 (PMC10603425; doi:10.1136/bmjopen-2022-071104)
Supplement: Supplementary data [file bmjopen-2022-071104supp001.pdf]

SUPPLEMENTARY MATERIAL

Title: The effects of education and age on experience of youth violence in a very low-income setting: a fixed-effects analysis from rural Burkina Faso

Supplementary Table 1a: Variables and their measurements

	Measurement
Outcome	
Violence experience (VE1)	Ever experienced bullying, physical attack or unwelcome sexual advances in the last 6 months
Bullying	Yes / No
Physical attack	Yes / No
Sexual violence	Yes / No
Violence perpetration (VP1)	Ever perpetrated bullying or physical attack in the last 6 months
Bullying	Yes / No
Physical attack	Yes / No
Individual level factors	
Age	Age in complete years
Education	
Level of schooling	Primary, post-primary or secondary level
School attainment	Grade/class/standard
Years of schooling	Years of education completed in years
Highest level of schooling	Primary, post-primary, secondary, technical/vocational or university
Marital status	Single, engaged, married (monogamous), married (polygamous), civil union, separated/divorced or widowed
Experienced first sex	Had first sex in the last 6 months (Yes / No)
Multiple sex partners	Had sex with more than one person in your life time (Yes / No)
Number of sexual partners	Number of life time sexual partners
Employment	Done any work in the last 12 months to earn money or obtain food (Yes/ No)
Alcohol use	Ever drunk alcohol on your own (Yes / No)
Substance use	Ever used drugs (Yes / No)
Household level factors	
Household asset	Asset index for household
Household size	Number of persons living in household
Aged 0 – 14 years	
Aged 15 – 29 years	
Aged 30 – 44 years	
Aged 45 – 59 years	
Aged 60 and above	
Moving between household	Sometimes live in another household other than primary household (Yes / No)
Living with adult figure	Yes / No
Adult figure	Father / Mother
Age of adult figure	Age in complete years

Kuunibe N, *et al. BMJ Open* 2023; 13:e071104. doi: 10.1136/bmjopen-2022-071104

Less than 30 years	
30 – 45 years	
46 – 60 years	
Above 60 years	
No adult figure	
Educational level adult figure	Primary, post-primary, secondary, technical/vocational or university
Male	
Female	
Occupation of adult figure	Farmer, merchant, teacher, other government worker, other (specified)
Male	
Female	
Frequency of parental /guardian support	In the last 30 days how often did parent/ guardian……
With important life decisions	Helped you make important decisions (never, rarely, sometimes, most of the times or always)
With understanding problems	Understand your problems and worries (never, rarely, sometimes, most of the times or always)
Talking about what happens	Take time to talk with you about things that happen to you (never, rarely, sometimes, most of the times or always)
Paying attention to opinion	Pay attention to your opinion (never, rarely, sometimes, most of the times or always)
Media use	
Access to media	
Radio	Yes / No
TV	Yes / No
Magazines	Yes / No
Use of media	
Radio	Average number of hours per day spent listening to radio
TV_1	Frequency of watching TV [never, rarely (some hours per month), often (some hours per week) or very frequently (several hours per day)]
TV_2	Average number of hour per day spent watching TV
Magazines	How often do you read magazines [never, rarely (some hours per month), often (some hours per week) or very frequently (several hours per day)]
Community level factor	
Access to education	Community share of school enrolment

Supplementary Table 2: Parental support (n=1291)

Variable	2017		2018	
	Frequency	Percent	Frequency	Percent
Parents understand worries				
Never	299	23.27	68	5.29
Rarely	201	15.64	193	15.01
Sometimes	271	21.09	391	30.40
Most of the time	159	12.37	295	22.94
Always	351	27.32	339	26.36
Missing	4	0.31		
Parents help make decisions				
Never	212	16.50	27	2.10
Rarely	257	20.00	193	15.01
Sometimes	311	24.20	350	27.22
Most of the time	159	12.37	331	25.74
Always	337	26.23	384	29.86
Missing	9	0.70	1	0.08
Parents talk about things that happen				
Never	299	23.27	83	6.45
Rarely	253	19.69	268	20.84
Sometimes	324	25.21	333	25.89
Most of the time	161	12.53	340	26.44
Always	243	18.91	262	20.37
Missing	5	0.39		
Parents pay attention to what you say				
Never	317	24.67	94	7.31
Rarely	296	23.04	225	17.50
Sometimes	381	29.65	416	32.35
Most of the time	133	10.35	236	18.35
Always	153	11.91	314	24.42
Missing	5	0.39	1	0.08

We built a summative score of parental support by adding the four variables, with a minimum score of 0 and a maximum score of 16.

Kuunibe N, *et al. BMJ Open* 2023; 13:e071104. doi: 10.1136/bmjopen-2022-071104

Supplementary Table 3: Difference between those who dropped out in wave 2 and remaining sample.

	participation_w2 = 0	participation_w2 = 1	Test	Statistic	p-value
	N=353	N=1,291			
Number of physical attacks, last 12m	0 (0-0)	0 (0-0)	Wilcoxon rank-sum	Z= -0.74	0.46
Days bullied, last 30d	0 (0-0)	0 (0-0)	Wilcoxon rank-sum	Z= -1.18	0.24
Gender			Chi-square	Chi2(1)= 4.55	0.033
Female	167 (47.3%)	529 (41.0%)			
Male	186 (52.7%)	762 (59.0%)			
Age (years)	15 (14-18)	15 (13-17)	Wilcoxon rank-sum	Z= 3.64	<0.001
Marital status cat			Chi-square	Chi2(1)= 2.08	0.15
other	*41 (11.6%)*	*117 (9.1%)*			
single	*312 (88.4%)*	*1,174 (90.9%)*			
Ever worked	222 (62.9%)	786 (60.9%)	Chi-square	Chi2(1)= 0.47	0.49
Mother is alive	342 (96.9%)	1,261 (97.7%)	Chi-square	Chi2(1)= 0.72	0.40
Father is alive	316 (89.5%)	1,186 (91.9%)	Chi-square	Chi2(1)= 1.94	0.16
Lives with Mother	262 (74.2%)	1,005 (77.8%)	Chi-square	Chi2(1)= 2.06	0.15
Lives with father	245 (69.4%)	965 (74.7%)	Chi-square	Chi2(1)= 4.07	0.044
Lives alone	23 (6.5%)	56 (4.3%)	Chi-square	Chi2(1)= 2.87	0.090
Has own bedroom	58 (16.4%)	228 (17.7%)	Chi-square	Chi2(1)= 0.29	0.59
Wealth quintile			Chi-square	Chi2(4)= 1.12	0.89
1	*76 (21.5%)*	*255 (19.8%)*			
2	*74 (21.0%)*	*257 (19.9%)*			
3	*73 (20.7%)*	*282 (21.8%)*			
4	*64 (18.1%)*	*235 (18.2%)*			
5	*66 (18.7%)*	*262 (20.3%)*			
Currently in school	119 (33.7%)	703 (54.5%)	Chi-square	Chi2(1)= 47.71	<0.001
Current school level			Chi-square	Chi2(4)= 53.56	<0.001
None	*234 (67.2%)*	*589 (46.2%)*			
Primary (1-6)	*40 (11.5%)*	*268 (21.0%)*			
Post Primary (7-10)	*66 (19.0%)*	*386 (30.3%)*			
Secondary (1-3)	*7 (2.0%)*	*33 (2.6%)*			
Technical/Vocational	*1 (0.3%)*	*0 (0.0%)*			
Has access to TV	295 (83.6%)	1,035 (80.2%)	Chi-square	Chi2(1)= 2.07	0.15
Frequency of watching TV			Chi-square	Chi2(3)= 2.69	0.44
Never	*59 (16.8%)*	*263 (20.4%)*			
Rarely (some hours per month)	*88 (25.0%)*	*290 (22.5%)*			

Often (several hours per week)	*151 (42.9%)*	*540 (41.9%)*			
Very often (several hours per day)	*54 (15.3%)*	*197 (15.3%)*			
Frequency of reading magazines			Chi-square	Chi2(3)= 6.12	0.11
Never	*334 (94.6%)*	*1,177 (91.2%)*			
Rarely (some hours per month)	*8 (2.3%)*	*30 (2.3%)*			
Often (several hours per week)	*5 (1.4%)*	*30 (2.3%)*			
Very often (several hours per day)	*6 (1.7%)*	*54 (4.2%)*			
Ever had intercourse?	*55 (16.8%)*	*202 (17.0%)*	*Chi-square*	*Chi2(1)= 0.01*	*0.93*
Number of sex partners, lifetime			Chi-square	Chi2(11)= 6.01	0.87
0	*298 (86.4%)*	*1,087 (86.3%)*			
1	*29 (8.4%)*	*121 (9.6%)*			
2	*9 (2.6%)*	*25 (2.0%)*			
3	*5 (1.4%)*	*14 (1.1%)*			
4	*1 (0.3%)*	*3 (0.2%)*			
5	*0 (0.0%)*	*4 (0.3%)*			
6	*1 (0.3%)*	*1 (0.1%)*			
7	*0 (0.0%)*	*1 (0.1%)*			
8	*1 (0.3%)*	*1 (0.1%)*			
9	*0 (0.0%)*	*1 (0.1%)*			
10	*1 (0.3%)*	*1 (0.1%)*			
17	*0 (0.0%)*	*1 (0.1%)*			
Scores for component 1	-.2998273 (-1.694384-.8893526)	-.6083845 (-1.707631-.9265742)	Wilcoxon rank-sum	Z= 0.57	0.57
Parental_support	2 (1-2.75)	1.75 (1-2.75)	Wilcoxon rank-sum	Z= 0.71	0.48

Data are presented as median (IQR) for continuous measures, and n (%) for categorical measures.

Supplementary Table 4: Full regression models predicting violence experiences

Variables	Bullying		Physical Attacks	
	Model 2 Adjusted	Model 3 Adjusted & interaction	Model 2 Adjusted	Model 3 Adjusted & interaction
Interview 2 vs interview 1	-0.42[-0.75,-0.08]	-0.44 [-0.78,-0.11]	-0.10[-0.27,0.07]	-0.09[-0.26,0.08]
Marital status (vs other)				
Single	-0.20[-1.03,0.63]	-0.24[-1.07, 0.59]	0.03[-0.40,0.46]	0.05[-0.38,0.48]
Ever worked	0.01[-0.27,0.28]	0.01[-0.27,0.28]	0.05[-0.09,0.20]	0.05[-0.09,0.19]
Number of people in the household	-0.04 [-0.07,-0.02]	-0.04 [-0.07,-0.02]	-0.01[-0.02,0.00]	-0.01[-0.02,0.00]
Mother is alive	0.74[-1.34, 2.82]	0.77[-1.31,2.85]	-0.30[-1.38,0.77]	-0.32[-1.40,0.76]
Father is alive	1.12[-2.41,0.17]	-1.11[-2.41, 0.18]	-0.35[-0.99,0.28]	-0.34[-0.98,0.29]
Lives with Mother	-0.12[-0.74,0.51]	-0.15[-0.77,0.48]	0.06[-0.26,0.39]	0.08[-0.25,0.40]
Lives with father	0.46[-0.18,1.10]	0.45[-0.18,1.09]	-0.13[-0.46,0.20]	-0.12[-0.45,0.21]
Lives alone	-0.17[-1.03,0.69]	-0.14[-1.00,0.72]	-0.38[-0.83,0.07]	-0.40[-0.84,0.05]
Parental support	-0.18 [-0.31,-0.05]	-0.18 [-0.31,-0.05]	-0.04[-0.11,0.03]	-0.04[-0.11,0.03]
Household wealth quintile				
Lowest (base)				
2	0.08[-0.34,0.50-]	0.06[-0.36,0.49]	-0.05[-0.27,0.17]	-0.04[-0.26,0.17]
3	-0.01[-0.44,0.42]	-0.02[-0.44,0.41]	-0.06[-0.28,0.16]	-0.06[-0.28,0.16]
4	-0.04[-0.52,0.44]	-0.07[-0.55,0.41]	-0.08[-0.32,0.17]	-0.06[-0.31,0.18]
Highest	0.43[-0.15,1.00]	0.40[-0.18,0.98]	0.12[-0.18,0.41]	0.13[-0.17,0.42]
Has own bedroom	-0.08[0.47,0.32]	-0.08[-0.47,0.32]	-0.01[-0.21,0.20]	-0.01[-0.21,0.20]
Radio hours/day	0.13[-0.01,0.27]	0.13[-0.00,0.27]	-0.02[-0.09,0.05]	-0.02[-0.09,0.05]
Frequency of watching television				
Never (base)				
Rarely (some hours per month)	-0.34[0.72,0.03]	-0.29[-0.67,0.09]	0.06[-0.13,0.26]	0.03[-0.16,0.23]
Often (several hours per week)	-0.11[-0.46,0.24]	-0.07[-0.42,0.29]	-0.04[-0.22,0.14]	-0.06-0.24,0.12]
Very often (several hours per day)	0.63 [0.14,1.11]	0.63 [0.15,1.12]	-0.19[00.44,0.06]	-0.19[-0.44,0.06]
Frequency of reading magazines				

Kuunibe N, *et al. BMJ Open* 2023; 13:e071104. doi: 10.1136/bmjopen-2022-071104

Never (base)				
Rarely (some hours per month)	1.01 [0.46,1.57]	0.99 [0.43,1.54]	-0.18[-0.47,0.11]	-0.17[-0.46,0.12]
Often (several hours per week)	1.12 [0.38,1.85]	1.08 [0.35,1.81]	-0.22[-0.61,0.16]	-0.21[-0.59,0.17]
Very often (several hours per day)	-0.17[-1.06,0.73]	-0.07[-0.97,0.83]	0.90 [0.44,1.36]	0.85 [0.38,1.31]
Household size missing	-1.77[-3.73, 0.20]	-1.68[-3.64, 0.29]	-0.46[-1.39,0.46]	-0.50[-1.43,0.43]
Freq. of reading magazine missing	6.99 [4.23, 9.75]	6.93 [4.16,9.69]	0.46[-0.97,1.89]	0.49[-0.94,1.92]

All models present coefficients and 95% confidence intervals. All models contain fixed effects for each respondent. Age centred at 15.

Kuunibe N, *et al. BMJ Open* 2023; 13:e071104. doi: 10.1136/bmjopen-2022-071104
