# Supplementary material for: Effects of education and age on the experience of youth violence in a very low-resource setting: a fixed-effects analysis in rural Burkina Faso
Source: BMJ Open. 2023 Oct 18;13(10):e071104. doi: 10.1136/bmjopen-2022-071104 (PMC10603425; doi:10.1136/bmjopen-2022-071104)
Supplement: Author's manuscript [file bmjopen-2022-071104.draft_revisions.pdf]

BMJ Open

BMJ Open is committed to open peer review. As part of this commitment we make the peer review history of every article we publish publicly available.

When an article is published we post the peer reviewers' comments and the authors' responses online. We also post the versions of the paper that were used during peer review. These are the versions that the peer review comments apply to.

The versions of the paper that follow are the versions that were submitted during the peer review process. They are not the versions of record or the final published versions. They should not be cited or distributed as the published version of this manuscript.

BMJ Open is an open access journal and the full, final, typeset and author-corrected version of record of the manuscript is available on our site with no access controls, subscription charges or pay-per-view fees (http://bmjopen.bmj.com).

If you have any questions on BMJ Open's open peer review process please email info.bmjopen@bmj.com

BMJ Open

The effects of education and age on the experience of youth violence in a very low-resource setting: a fixed-effects analysis in rural Burkina Faso.

Journal:	*BMJ Open*
Manuscript ID	bmjopen-2022-071104
Article Type:	Original research
Date Submitted by the Author:	16-Dec-2022
Complete List of Authors:	Kuunibe, Naasegnibe; Simon Diedong Dombo University of Business and Integrated Development Studies,<br>Bountogo, Mamadou; Centre de Recherche en Sante de Nouna, Nouna; Université de Ouagadougou, Ouaga<br>Ouermi, Lucienne; Centre de Recherche en Sante de Nouna<br>Sié, Ali; Centre de Recherche en Sante de Nouna Nouna, Boucle du Mouhoun, BF<br>Bärnighausen, Till ; Heidelberg University, Heidelberg Institute of Global Health<br>Harling, Guy; University College London, Institute for Global Health; Harvard University T H Chan School of Public Health, Department of Epidemiology
Keywords:	HEALTH ECONOMICS, PUBLIC HEALTH, STATISTICS & RESEARCH METHODS

SCHOLARONE™
Manuscripts

I, the Submitting Author has the right to grant and does grant on behalf of all authors of the Work (as defined in the below author licence), an exclusive licence and/or a non-exclusive licence for contributions from authors who are: i) UK Crown employees; ii) where BMJ has agreed a CC-BY licence shall apply, and/or iii) in accordance with the terms applicable for US Federal Government officers or employees acting as part of their official duties; on a worldwide, perpetual, irrevocable, royalty-free basis to BMJ Publishing Group Ltd ("BMJ") its licensees and where the relevant Journal is co-owned by BMJ to the co-owners of the Journal, to publish the Work in this journal and any other BMJ products and to exploit all rights, as set out in our licence.

The Submitting Author accepts and understands that any supply made under these terms is made by BMJ to the Submitting Author unless you are acting as an employee on behalf of your employer or a postgraduate student of an affiliated institution which is paying any applicable article publishing charge ("APC") for Open Access articles. Where the Submitting Author wishes to make the Work available on an Open Access basis (and intends to pay the relevant APC), the terms of reuse of such Open Access shall be governed by a Creative Commons licence – details of these licences and which Creative Commons licence will apply to this Work are set out in our licence referred to above.

Other than as permitted in any relevant BMJ Author's Self Archiving Policies, I confirm this Work has not been accepted for publication elsewhere, is not being considered for publication elsewhere and does not duplicate material already published. I confirm all authors consent to publication of this Work and authorise the granting of this licence.

For peer review only

The effects of education and age on the experience of youth violence in a very low-resource setting: a fixed-effects analysis in rural Burkina Faso.

Naasegnibe Kuunibe[0,2*], Mamadou Bountogo[3], Lucienne Ouermi[3], Ali Sié[3], Till Bärnighausen[2,4,5,], Guy Harling[4,6,7,8,9]

* Corresponding Author:

P O Box WA64, Wa, Upper West Region Ghana

nkuunibe@ubids.edu.gh / +233(0)209006165.

1. Department of Economics, Faculty of Social Science and Arts, SD-Dombo University of Business and Integrated Development Studies, Wa, Ghana
2. Heidelberg Institute for Global Health, Heidelberg University Hospital, Heidelberg, Germany
3. Centre de Recherche en Santé de Nouna, Nouna, Burkina Faso
4. Africa Health Research Institute, KwaZulu-Natal, South Africa
5. Department of Global Health and Population, Harvard T.H. Chan School of Public Health, Boston, MA, USA
6. Institute for Global Health, University College London, London, United Kingdom
7. MRC/Wits Rural Public Health & Health Transitions Research Unit (Agincourt), University of the Witwatersrand, South Africa
8. Department of Epidemiology & Harvard Center for Population and Development Studies, Harvard T.H. Chan School of Public Health, Boston, MA, USA
9. School of Nursing & Public Health, College of Health Sciences, University of KwaZulu-Natal, KwaZulu-Natal, South Africa

Keywords: Adolescence, physical violence, bullying, panel fixed-effects, Burkina Faso.

Word count: 3679

ABSTRACT

Youth violence is both a public health and an economic problem. Cross-sectional evidence from low- and middle-income settings suggests individual-level factors influence adolescent

For peer review only - http://bmjopen.bmj.com/site/about/guidelines.xhtml

violence experience. However, longitudinal evidence on the effect of age and education on youth violence in such settings is limited, especially where educational attainment is low.

Methods: We analysed two waves of data from 1644 adolescents in north-western Burkina Faso from 2017 and 2018. We examined experience of physical attack in the past 12 months and of bullying in the past 30 days. Our exposures were completed years of age and educational attainment. We used panel fixed-effects OLS regression models to analyse the associations of interest adjusting for individual- and household-level time-varying covariates and tested for effect-modification by gender.

Results: A substantial minority of respondents experienced violence in both waves (24.1% bullying and 12.2% physical attack), with males experiencing more violence. Bullying was positively associated with more education (β=0.12; 95% confidence interval [CI]: 0.02, 0.22) and non-significantly with older age. Both effects were stronger in males than females, although the gender differences were not significant. Physical attacks fell with increasing age (β=-0.18; 95% confidence interval [CI]: -0.31, -0.05) and this association was again stronger in males than females; education and physical attacks were not substantively associated.

Conclusions: Bullying and physical attacks are commond for rural adeolscent Burkinabe. The age patterns found suggest that, particularly for males, there is a need to target violence prevention at younger ages and bullying prevention at slightly older ones, particularly for those remaining in school. Nevertheless, a fuller understanding of the mechanisms behind our findings are needed to design effective interventions to protect youth in low-income settings from violence.

INTRODUCTION

Violence globally represents both public health and an economic problem. In health terms, violence generates both mortality and morbidity. Violence is estimated to cause 1.3 million

For peer review only - http://bmjopen.bmj.com/site/about/guidelines.xhtml

deaths annually, accounting for 2.5% of global mortality,[1]. Violence often requires acute health service access (e.g., assault victims requiring emergency hospital care) and can result in long-term physical disability, depression or reproductive health problems,[1, 2]. Violence also affects local economies through workforce absenteeism, loss of productivity and loss of human capital. Families can fall into poverty if a breadwinner dies or becomes permanently disabled due to violence,[1, 3].

Youth violence – which includes bullying, physical fighting, sexual and physical assault and homicide – is particularly problematic because it generates higher economic, welfare and criminal justice costs,[4]. Besides death, injury and psychological harm, youth violence can lead to increased subsequent health risks behaviours such as smoking, substance abuse, unsafe sex and further violence,[5, 6]. An estimated 200,000 youth homicides occur each year, 83% among males, nearly all in in low- and middle-income countries (LMICs), with particularly high rates in Latin America and the Caribbean (LAC) and sub-Saharan Africa [4]. In sub-Saharan Africa in particualar, historical, economic and social factors continue to expose youth to violence,[7, 8]. Although violence affects all youth, adolescent girls and young women (AGYW) in LMICs appear to be most affected,[9-11]. Social and cultural norms, such as arranged and teenage marriage and denial of resource access to women, often keep AGYW economically dependent on males and thus vulnerable to abuse, especially from intimate partners,[12-14]. AGYW experience all forms of violence (emotional, physical and sexual), often perpetrated by other females in domestic and social settings,[15-18].

Many factors have been proposed as determinants of youth violence receipt and perpetration in LMICs, often adopting or adapting Heise's integrated ecological framework for violence against women,[19, 20]. This framework conceptualizes violence as a multifaceted phenomenon grounded in an interplay among personal, situational and sociocultural factors.

For peer review only - http://bmjopen.bmj.com/site/about/guidelines.xhtml

We modified this conceptualization to cover all forms of adolescent violence, concentrating on individual-level and microsystem (family/household/relationship) factors given our focus within a specific geography. We focus on two individual-level factors believed to play an important role in determining violence experience: age and education. Given the magnitude of youth violence, evidence of associations between violence, age and educational attainment, and calls for youth violence interventions,[1, 2], causal evidence on whether policies based on age or education are likely to affect violence levels is important. Past longitudinal analyses of adolescent violence often focused on the consequences of violence experience, rather than predictors of violence itself,[21, 22]. We instead focus on predictors of adolescent violence experience, to contribute to the upstream prevention of violence, rather than efforts to break the connection between violence and its sequelae.

Age is strongly associated with both the experience and perpetuation of violence,[3, 23]. Youth aged 15 to 29 are more likely to both experience and perpetrate violence than older adults, often experiencing violence perpetrated by their older peers or family members,[3, 23]. Males are more likely to perpetrate violence, while young girls and women are more likely to experience it,[9, 10, 14, 24, 25]. However, evidence on the causal effect of age on violence is comparatively scarce, with few even cross-sectional studies explicitly focused on youth,[3, 11, 26].

Education is theorized to protect against violence, since more-educated persons are less likely to either perpetrate or experience violence,[26, 27]. However, causal evidence on the effect of education on youth violence in sub-Saharan Africa is again limited. Two studies have used changes in national school policy as natural experiments in this context. One focused on violence, finding that a one-year increase in grade attainment was associated to a nine percentage-point reduction in the probability of experiencing sexual violence in Uganda, but

For peer review only

For peer review only - http://bmjopen.bmj.com/site/about/guidelines.xhtml

no significant effect in Malawi,[27]. A second focused on sexual health, finding an additional year of schooling was associated with 0.11 fewer births and 14 percentage points less teen marraige in Ghana,[28]. Both of these studies necessarily assess the overall impact of policy change, rather than the increase in education alone, and it is unclear how their findings extrapolate to lower educational attainment settings.

Burkina Faso is a landlocked country in West Africa, which despite economic and political reforms remains one of the poorest in the world, with about half of its population living below the international poverty line,[29]. Economic deprivation is strongly associated with youth violence,[7, 30, 31]. The country is very young, with around 45% aged under fifteen and a further 20% aged 15-24 in 2015,[29, 32]. Educational access and youth literacy are limited, with only 13% of adults having completed primary education,[32, 33]. Within Burkina Faso, poverty is highest in the Boucle du Mouhoun region,[34]. Violence experience is common for young Burkinabe, with lifetime physical violence prevalence reported at 47-80% and sexual violence at 33-51%,[35, 36]. Adverse psychological and mental health outcomes commonly follow such experiences,[37, 38]. However, studies of Burkinabe youth violence have generally used cross-sectional designs and have not explored the effects of education or age specifically,[3, 39].

We therefore analysed longitudinal data on adolescents in Boucle de Mouhoun to assess the effects of age and education on violence experience. The potential for educational interventions to have violence-specific benefits in such high-poverty, low-education settings is likely to be substantial,[37, 38]. By using fixed-effects analysis we were able to exclude time-specific and time-invariant confounders, something particularly important given the many unobserved predictors of violence perpetration and victimization,[22, 40].

For peer review only - http://bmjopen.bmj.com/site/about/guidelines.xhtml

METHODS

Study design

We used data from the Nouna Health and Demographic Surveillance Site (HDSS) in north western Burkina Faso, which has been gathering demographic and epidemiological health information data since 1992. The 59 villages and the town of Nouna that comprise the HDSS have a population of slightly over 100,000 individuals and include one hospital and 13 primary health centres (CSPS),[41].

Our study used longitudinal data from two Burkina Faso waves of the Africa Research, Implementation Science and Education (ARISE) adolescent health study,[42], collected in the Nouna HDSS in 2017 and 2018. Data were collected from 1644 adolescents aged 12-19 in 2017, based on a stratified random sample of 2544 age-eligible residents in Nouna town and ten villages,[42, 43]. A follow-up round was conducted in 2018, attempting to contact all those who participated in 2017; 1291 interviews were completed. In both years a standardized questionnaire was used to collect self-reported information on socio-demographics, health practices, health outcomes and risk factors.

Patient and Public Involvement

No patient involved

Measures

We used two primary outcomes of youth violence, both captured as count variables: experience of physical attack in the past 12 months; and experience of bullying in the past 30 days. We also generated binary measures of any experience for each outcome, in alignment with the World Report of Violence and Health's definitions,[2]. Our exposures were age (in completed years) and education (years of fulltime education).

For peer review only - http://bmjopen.bmj.com/site/about/guidelines.xhtml

We additionally considered a range of time-varying covariates at the individual and household levels (based on Heise,[20] framework,[31]), plus media use,[44, 45]. Specifically, our individual-level covariates were: currently in school; marital status (never married vs all else); any work in the past 12 months; and sexual behaviour (sexual debut and number of sexual partners). Our household-level covariates were: household size; household wealth quintile; parental vital status; parental support level (16 point scale, converted to the first principal component of the four variables included); parental co-residence (respondent lives with both parents, only mother, only father or lives alone); respondent has their own bedroom. Our media covariates were: any access to television; and frequency of watching television or reading magazines (never, rarely, often, very often). Full variable definitions are provided in **Error! Reference source not found.** and **Error! Reference source not found.**.

Statistical analyses

We first described our data using frequency and percentages in both waves, including a comparison of those lost-to-follow-up vs those completing both waves. We then dropped any respondents who were missing data for the outcome variables, either due to preferring not to respond, not know their answer or where fieldwork errors affected responses, while for some the question was not applicable.

When considering the causal effect of education and age on youth violence, a major concern is unobserved confounding. Given the difficulty of implementing randomized controlled trials, since age is not directly manipulatable and intentional exposure to violence unethical,[46, 47], we exploited the panel nature of the data structure to run fixed-effects regression models to remove all time-invariant confounding. We specified our model as:

$$y_{it} = \alpha_i + \beta X_{it} + \gamma Z_{it} + \delta_t + \rho_i \qquad (1)$$

For peer review only - http://bmjopen.bmj.com/site/about/guidelines.xhtml

Where y_{it} is youth violence for each individual i at each time point t, X_{it} represents our time-varying exposures (education and age), Z_{it} is other time-varying factors for each individual, δ_t is a period-specific fixed effect to capture all individual-invariant factors and ρ_i are individual-specific fixed-effects which capture all time-invariant factors for each individual, e.g., gender, ethnicity, underlying proclivity to violence.

For each outcome (bullying and physical attack), we implemented three linear regression models using count outcome variables (Poisson and Negative Binomial models did not converge). Model 1 considered only mean-centred age and years of full-time education. In model 2, we add all time-varying covariates. In model 3, we included interaction terms for gender with age and education to identify any gender-specific effects.

RESULTS

Description of sample

At baseline in 2017, 1,644 young people were interviewed, of whom 948 (57.7%) were male. By 2018, 21.5% of respondents, comprising 167 (24.0%) females and 186 (19.6%) males, were lost to follow up, leaving 1,291 respondents who participated in both rounds. We dropped 32 individuals (64 data points) who had missing values for the question on bullying, leaving 1,258 respondents for the bulling analysis. Similarly, we dropped 14 individuals (28 data points) who did not answer physical attack question to arrive at 1,276 respondents.

The 1,291 respondents were aged 12 to 20 years in 2017: median 15.5, interquartile range (IQR): 14-18 (**Error! Reference source not found.**A). Around half were enrolled in school at each interview: 703 (54.5%) in 2017; 671 (52.0%) in 2018 (**Error! Reference source not found.**B).

[Figure 1A about Here]

For peer review only - http://bmjopen.bmj.com/site/about/guidelines.xhtml

[[Figure 1B about Here]]

Around one-quarter of respondents never completed a year of fulltime education, while most others had at most attended primary or post-primary level. Over 90% of respondents were single at both interviews, and less than 20% in both 2017 and 2018 reported ever having sexual intercourse (Table 1). The proportion of respondents who ever worked fell from over 60% in 2017 to under 42% in 2018. Most respondents had living fathers (over 97%) and mothers (over 91%), however around one-quarter did not live with their parents. Media access was mixed: about 20% had no access to television in 2017 but around 15% watched several hours a day (access dropped by 2018); magazine reading was rare. Household wealth was by design evenly distributed across wealth quintiles. Households were large, with a median of 8 or 9 members.

For peer review only

Table 1. Descriptive statistics of independent variables

	Bullying		Physical attacks	
	**2017**	**2018**	**2017**	**2018**
**N**	1253		1271	
Marital status				
Single vs. all other	90.7	89.6	90.8	89.6
Living situation				
Ever worked vs never	624	42.1	61.4	41.7
Mother is alive	97.6	96.8	97.7	97.0
Father is alive	91.7	89.7	91.9	89.8
Lives with mother	78.0	80.1	77.7	80.3
Lives with father	74.8	76.1	74.6	76.1
Lives alone	4.5	0.2	4.4	0.2
Has own bedroom	18.0	19.9	17.8	19.7
Household wealth quintile				
Lowest	19.0	20.8	19.8	21.0
Second lowest	19.5	20.4	20.0	20.7
Middle	22.4	19.0	22.1	19.0
Second highest	18.4	20.1	17.9	20.2
Highest	20.8	19.6	20.2	19.1
Education				
Currently in school	54.0	51.6	54.6	52.1
Highest school level				
None	46.1	48.4	45.5	47.9
Primary (1 – 6)	20.3	10.8	20.8	11.3
Post-primary (7 – 10)	29.9	33.6	30.1	33.7
Secondary (1 – 3)	2.6	3.9	2.6	3.9
University		0.1		0.1
Not applicable	1.1	3.2	1.1	3.2

For peer review only - http://bmjopen.bmj.com/site/about/guidelines.xhtml

Media use				
Has access to television	80.5	70.6	80.1	70.3
Frequency of watching television				
Never	20.1	30.8	20.5	31.0
Rarely (some hours per month)	22.8	22.8	22.7	22.8
Often (several hours per week)	42.1	36.2	41.8	36.1
Very often (several hours per day)	15.1	10.1	15.1	10.2
Frequency of reading magazines				
Never	90.9	87.1	91.1	87.3
Rarely (some hours per month)	2.4	8.5	2.23	8.4
Often (several hours per week)	2.4	4.1	2.4	3.9
Very often (several hours per day)	4.3	0.3	4.3	0.3
Sexual behaviour				
Ever had intercourse	17.3	19.4	15.8	18.9
Number of lifetime sexual partners				
1	9.7	13.5	9.4	13.3
2 – 7	3.8	5.2	3.8	5.3
8 – 17	0.3		0.3	
No response	2.1	1.3	2.4	0.3
Household size †	9 [6, 12]	8.5 [7, 11]	8.5 [6, 12]	8 [7, 11]
Parental support†	7 [4, 12]	10 [7, 13]	7 [4, 11]	10 [7, 13]

All values are percent except † which are medians and interquartile ranges.

A substantial minority of respondents experienced bullying and physical attacks (Table 2). Overall, 189 females (18.9%) and 416 males (27.6%) experienced bullying in the 30 days preceding the interview, while 111 females (10.6%) and 199 males (13.2%) experienced physical attacks in the preceding 12 months. Across both rounds, males experienced both more violence than females: males experienced 416 of 605 (68.8%) unique bullying instances in the past 30 days, and 199 of 310 (64.2%) unique violence instances in the past 12 months. Bullying experience declined slightly with age – from over 26% among under 15s to 21% among over 18s; physical attacks fell more sharply, especially in early adolescence.

Table 2: Distribution of violence experience across observations by age and gender

Variable	N	Bullying (%)	N	Physical attacks (%)
Gender				
Female	996	189 (18.9)	1,038	111 (10.6)
Male	1,510	416 (27.6)	1,504	199 (13.2)
Age				
12	19	52 (26.1)	210	51 (24.3)
13	387	100 (25.8)	398	73 (18.3)
14	389	104 (26.7)	403	65 (16.1)

For peer review only - http://bmjopen.bmj.com/site/about/guidelines.xhtml
For peer review only

15	327	76 (23.2)	331	30 (9.1)
16	286	67 (23.4)	288	22 (7.6)
17	274	64 (23.4)	272	27 (9.9)
18	297	63 (21.2)	295	20 (6.8)
19	214	43 (20.1)	212	15 (7.1)
20	130	35 (26.9)	130	7 (5.4)
21	3	1 (33.3)	3	0 (0.0)
Total	2506	605 (23.8)	2542	310 (12.2)

Note: Each individual is represented twice in this table, once per survey round.

Fixed-effects analysis

In bivariate fixed-effects regression, older age was associated with non-significantly more bullying (0.14 more bullying experiences per month for each additional year of age, 95% confidence interval [CI]: -0.12, 0.39) and associated with significantly fewer physical attacks (-0.19, 95%CI: -0.32, -0.06). More education was associated with significantly more bullying (0.11 bullying experiences per month for each additional year of schooling, 95%CI: 0.01, 0.21), but not with physical attacks (Table 3, Model 1). Controlling for time-varying potential confounders attenuated the association of age and bullying but otherwise had limited effects on our relationship of interest (Table 3, Model 2). When we allowed effects to vary by gender (Table 3, Model 2), associations in all four models were more positive for men than for women, with wider gaps for the impact of age on both outcomes than for education. All these results were independent of a substantial but imprecise negative association between currently being in school and bullying or attacks.

For peer review only

For peer review only - http://bmjopen.bmj.com/site/about/guidelines.xhtml

Table 3: OLS models predicting count of violence events

	Model 1 Unadjusted	Model 2 Adjusted	Model 3 Adjusted & interaction	Interaction test
Bullying in the last 30 days (N=2506)				
Age in years	0.14 [-0.12, 0.39]	0.08 [-0.17, 0.33]		
Female			-0.08 [-0.39, 0.24]	
Male			0.18 [-0.10, 0.45]	F=2.76, p=0.10
Fulltime education in years	0.11 [0.01, 0.21]	0.12 [0.02, 0.22]		
Female			0.07 [-0.11, 0.26]	
Male			0.14 [0.03, 0.26]	F=0.39, p=0.53
Currently in school		-0.43 [-1.05, 0.19]	-0.41 [-1.03, 0.21]	
Physical attacks in the last 12 months (N=2542)				
Age in years	-0.19 [-0.32, -0.06]	-0.18 [-0.31, -0.05]		
Female			-0.10 [-0.26, 0.05]	
Male			-0.23 [-0.38, -0.09]	F=2.74, p=0.10
Fulltime education in years	0.02 [-0.03, 0.07]	0.04 [-0.01, 0.09]		
Female			0.06 [-0.03, 0.16]	
Male			0.03 [-0.03, 0.09]	F=0.41, p=0.52
Currently in school		-0.27 [-0.59, 0.05]	-0.27 [-0.59, 0.04]	

The table presents four regression models per column, all showing point estimates and 95% confidence intervals. All models contain fixed-effects for each respondent and interview round. Age centred at 15. Models 2 and 3 are also adjusted for marital status, ever worked, household size, parent alive, living with parents, household wealth, has own bedroom and access to media; full results available in ***Error! Reference source not found.***

DISCUSSION

In this study we employed individual and time fixed-effects models to assess the effects of age and education on violence experience in the form of bullying and physical attacks among adolescents and young adults in a panel study in rural Burkina Faso. We found bullying experience prevalence in the past 30 days ranging from 20-30%, and physical attack experience of 10-15% in the previous 12 months. While there is little directly comparable data in Burkina Faso, both levels seem concerning, if in line with studies elsewhere,[3, 39]. In fixed-effects models we found bullying was associated with more education and weakly with greater age, both effects stronger in males, while physical attacks were associated with younger age (again more strongly for males) but not with education.

For peer review only - http://bmjopen.bmj.com/site/about/guidelines.xhtml

Our findings on the effect of age on youth experience of physical attacks are consistent with some observational evidence elsewhere, for example physical violence from both peers and caregivers falls with age in Latin America and the Caribbean,[39]. Evidence on the causal effect of age on violence is rare, with even cross-sectional studies focused on youth uncommon,[3, 11, 26]. Studies of intimate partner violence (IPV) amongst women suggest rates are higher in older teenage girls compared to adults,[48, 49], but this does not allow within-adolescence comparisons. The faster decline with age that we see for males (from a higher initial level) is important to note: while criminal interpersonal violence appears peaks around age 18 in many settings, our findings and past work suggest that overall frequency of violence experience may in fact decline across teenage years, at least in non-urban settings,[50]. In combination, this evidence suggests a shift in violence experience composition for adolescent males that would be worth further investigation.

The implications of this negative association between age and violence experience in adolescence depend on what mechanisms are generating them. First, age might be a proxy for predictors of violence that we have not captured in this analysis. This might include adolescent autonomy in decision-making, for example relating to bedtime and the amount and type of television watched, which typically rises with age,[51]: adolescent autonomy was negatively associated with youth violence among US Latino youth,[52]. Alternatively, age might be a distal determinant of factors leading more directly to violence experience. For example, older adolescents may be better able to protect themselves against aggressive behaviour from their peers or adults. If this is the case, then structural interventions (at the family, community or national levels) or behaviour change interventions (e.g., teaching adolescents how to avoid confrontations) might be beneficial in protecting younger adolescents,[53, 54]. Further research to understand these causal mechanisms, and thus design effective interventions, will

For peer review only - http://bmjopen.bmj.com/site/about/guidelines.xhtml
For peer review only

need to include more detailed quantitative data on who perpetrates violence against younger adolescents and qualitative information on how and why such violence comes about.

Our finding that bullying increases with age contradicts some past resaerch. Observationally, bullying victimization rates are higher in younger children than in older adolescents, both in the US and in sub-Saharan Africa,[55, 56]. Our results may reflect the stronger control our fixed-effects approach provides against between-individual and temporal confounding, suggesting that the decline in bullying seen elsewhere is a function of factors associted with age, rather than age itself. Our finding of a stronger association for males adds to a mixed literature, aligning with studies from Taiwan and Saskatchwan Canada,[57, 58], but in contrast to findings from the US and Manitoba Canada,[57, 59].

We found that education was not associated with violence experience in our setting. A similar null effect of education (grade attainment) was reported for 19-31 year old Malawian women,[27]. A recent meta-analysis of 86 studies in 60 LMICs noted that poor academic performance and weak school attachment were correlated with increased youth violence,[23], in contrast to our null findings. Again, more detail is available for IPV, with a Ugandan study finding that less-educated women were more likely to experience physical IPV – however this study included women aged 15-49, which makes direct comparison difficult,[60]. Other studies have confirmed the protective effect of education on violence in different settings,[48, 61]. The discrepancy between others' findings and ours may reflect the majority of past studies being cross-sectional, while we were able to use panel data. It may also reflect different exposures, since we considered quantity (years of schooling) rather than quality (performance or attachment).

Past evidence on the effect of education on bullying is mixed, with several studies finding lower bullying amongst those with more education,[56, 57] and few finding the opposite,[59].

For peer review only - http://bmjopen.bmj.com/site/about/guidelines.xhtml

Our finding of a positive association between education and bullying in both males and females may reflect the a true causal association, or the residual effect of being in school – given the opportunities that this provides for bullying relative to the alternative settings of field-based work or animal herding.

Finally, our analysis covers a population where half of adolescents are not now, and one-quarter never have been, attending school. The role of education in promoting or protecting against violence at the community level may be different in settings where education is not even close to universal. Further investigation of why our results looks different from other settings, including qualitative study of social norms surrounding violence across levels of educational attainment, would be instructional.

Strengths and limitations

A key strength of our study is the fixed-effects design which robustly removes time-invariant confounding of effect measures,[62]. Nevertheless, our study also has potential limitations: while fixed-effects analyses are powerful, they cannot control for unmeasured time-varying confounding and we may have therefore not fully accounted for factors that change rapidly among adolescents, such as increased social media access or social network change. The use of self-reported data may have led to reporting biases, although these would have had to vary differentially over time within respondents in order to bias our fixed-effects analyses. Generalizability from a sample drawn from a single district is always difficult to assess. However, by using a population-based sampling frame our analysis provides results that represent the entire local population and are likely to be broadly applicable in poor, rural settings in Burkina Faso and beyond.

For peer review only - http://bmjopen.bmj.com/site/about/guidelines.xhtml

Conclusion

A substantial minority of adolescents in rural north-western Burkina Faso report recent experiences of bullying or physical attack. The prevalence of these experiences was not significantly associated with years of education received, even within individual respondents, but did fall with age. A fuller understanding of the mechanisms behind this association is important in designing interventions to better protect youth in low-income settings from violence.

Contributorship statement

NK: Conceptualization, Methodology, Formal analysis, Writing – original draft. **MB**: Investigation, Writing – Review & Editing, Project administration. **LO**: Investigation, Writing – Review & Editing, Project administration. **AS**: Investigation, Resources, Writing – Review & Editing, Supervision. **TB**: Conceptualization, Methodology, Writing – Review & Editing, Project administration, Supervision, Funding acquisition. **GH**: Methodology, Data curation, Formal analysis, Writing – Review & Editing, Supervision, Project administration. All authors contributed revisions to the text, approved the final version and agree to be accountable for the work.

Competing interest

Authors declare no competing interests.

Funding

Funding for the study on which these data are based is provided by the Alexander von Humboldt Foundation through an award to Professor Bärnighausen. GH was supported by a fellowship from the Royal Society and the Wellcome Trust [Grant number 210479/Z/18/Z]. This research was funded in whole, or in part, by the Wellcome Trust [Grant number 210479/Z/18/Z]. For the purpose of open access, the authors have applied a CC BY public copyright licence to any Author Accepted Manuscript version arising from this submission.

For peer review only

For peer review only - http://bmjopen.bmj.com/site/about/guidelines.xhtml

Data sharing statement

The data that support the findings of this study are available from the corresponding author, TB, upon reasonable request and after signing a data use agreement. The data are not publicly available due to the risk of participant re-identification given the large amount of information contained for each person.

Ethical considerations

The Nouna ARISE study was approved by CRSN's Institutional Ethics Committee (2017/08 and 2018/017) and Heidelberg Medical Faculty's Ethics Commission (S-607/2018). Secondary data analysis was approved by University College London's Research Ethics Committee (15231/005). Prior to the study village elder provided oral assent. Each participants provided written informed consent; for respondents aged under 18, parents or guardians provided written consent alongside the minor's written assent. In cases of illiteracy, a literate witness assisted.

For peer review only

For peer review only - http://bmjopen.bmj.com/site/about/guidelines.xhtml

References

1. World Health organization, *Global Status report on Viloence Prevention*. 2014.

2. Krug, E.G., et al., *The world report on violence and health*, in *Lancet*. 2002. p. 1083-8.

3. Golshiri, P., et al., *Youth Violence and Related Risk Factors: A Cross-sectional Study in 2800 Adolescents.* Adv Biomed Res, 2018. **7**: p. 138.

4. World Health Organization, *Preventing youth violence: an overview of the evidence*. 2015.

5. Bellis, M.A., et al., *Adverse childhood experiences and associations with health-harming behaviours in young adults: surveys in eight eastern European countries.* Bulletin of the World Health Organization, 2014. **92**: p. 641-655.

6. World Health Organization. *Youth Violence*. 2019; Available from: https://www.who.int/health-topics/violence-against-children#tab=tab_3.

7. Ward, C.L., A. Dawes, and R. Matzopoulos, *Youth violence in South Africa: Setting the scene*, in *Youth violence: Sources and Solutions in South Africa*, C.L. Ward, A. Van der Merwe, and A. Dawes, Editors. 2013, University of Cape Town Press: Cape Town. p. 1-20.

8. Lamb, G., *Social cohesion and violence in South Africa: constructing a puzzle with missing pieces.* Crime, Law and Social Change, 2019. **72**(4): p. 365-385.

9. Rumble, L., et al., *Childhood sexual violence in Indonesia: a systematic review.* Trauma, Violence and Abuse, 2020. **21**(2): p. 284-299.

10. Iman'ishimwe Mukamana, J., P. Machakanja, and N.K. Adjei, *Trends in prevalence and correlates of intimate partner violence against women in Zimbabwe, 2005-2015.* BMC Int Health Hum Rights, 2020. **20**(1): p. 2.

For peer review only - http://bmjopen.bmj.com/site/about/guidelines.xhtml

11. Malta, D.C., et al., *Factors associated with family violence against adolescents based on the results of the National School Health Survey (PeNSE).* Cien Saude Colet, 2019. **24**(4): p. 1287-1298.

12. World Health Organization, *World Bank Group Gender Strategy (FY16-23) : Gender Equality, Poverty Reduction and Inclusive Growth*. 2015: World Bank, Washington, DC. © World Bank. .

13. Shamu, S., et al., *A systematic review of African studies on intimate partner violence against pregnant women: prevalence and risk factors.* PLoS One, 2011. **6**(3): p. e17591.

14. Girmay, A., et al., *Intimate partner physical violence and associated factors in reproductive age married women in Aksum Town, Tigray, Ethiopia 2018, and community based study.* BMC Res Notes, 2019. **12**(1): p. 627.

15. Breiding MJ, S.S., Basile KC, Walters ML, Chen J, & Merrick MT, *Prevalence and characteristics of sexual violence, stalking, and intimate partner violence victimization—National Intimate Partner and Sexual Violence Survey, United States, 2011.* Morbidity and Mortality Weekly Report, 2014. **63**(8): p. 1-18.

16. Baigorria, J., et al., *[Prevalence and associated factors with sexual violence against women: systematic review].* Rev Salud Publica (Bogota), 2017. **19**(6): p. 818-826.

17. Belay, S., et al., *Intimate partner violence and maternal depression during pregnancy: A community-based cross-sectional study in Ethiopia.* PLoS One, 2019. **14**(7): p. e0220003.

18. Semahegn, A. and B. Mengistie, *Domestic violence against women and associated factors in Ethiopia; systematic review.* Reprod Health, 2015. **12**: p. 78.

19. Puri, M., et al., *The prevalence and determinants of sexual violence against young married women by husbands in rural Nepal.* BMC research notes, 2012. **5**(1): p. 291.

20. Heise, L.L., *Violence against women: an integrated, ecological framework.* Violence Against Women, 1998. **4**(3): p. 262-90.

For peer review only - http://bmjopen.bmj.com/site/about/guidelines.xhtml

21. Ttofi, M.M., et al., *Do the victims of school bullies tend to become depressed later in life? A systematic review and meta-analysis of longitudinal studies.* Journal of Aggression, Conflict and Peace Research, 2011. **3**(2): p. 63-73.

22. Valido, A., et al., *Intra-familial Violence and Peer Aggression Among Early Adolescents: Moderating Role of School Sense of Belonging.* Journal of Family Violence, 2020. **36**: p. 87-98.

23. de Ribera, O.S., et al., *Correlates of youth violence in low-and middle-income countries: A meta-analysis.* Aggression and Violent Behavior, 2019. **49**: p. 101306.

24. Mason-Jones, A.J., et al., *Intimate partner violence in early adolescence: The role of gender, socioeconomic factors and the school.* South African Medical Journal, 2016. **106**(5): p. 502-509.

25. Masuda, K. and C. Yamauchi, *How Does Female Education Reduce Adolescent Pregnancy and Improve Child Health?: Evidence from Uganda's Universal Primary Education for Fully Treated Cohorts.* The Journal of Development Studies, 2020. **56**(1): p. 63-86.

26. Obradovic-Tomasevic, B., et al., *Prevalence and Predictors of Violence Victimization and Violent Behavior among Youths: A Population-Based Study in Serbia.* Int J Environ Res Public Health, 2019. **16**(17): p. 3203.

27. Behrman, J.A., A. Peterman, and T. Palermo, *Does Keeping Adolescent Girls in School Protect Against Sexual Violence? Quasi-Experimental Evidence From East and Southern Africa.* Journal of Adolescent Health, 2017. **60**(2): p. 184-190.

28. Adu Boahen, E. and C. Yamauchi, *The effect of female education on adolescent fertility and early marriage: Evidence from free compulsory universal basic education in Ghana.* Journal of African Economies, 2018. **27**(2): p. 227-248.

29. World Health Organization. *WHO Country Cooperation Strategy at a glance: Burkina Faso.* 2016 [cited 2019 19/03/2019]; Available from: https://apps.who.int/iris/handle/10665/136973.

For peer review only

For peer review only - http://bmjopen.bmj.com/site/about/guidelines.xhtml

30. de Ribera, O.S., et al., *Correlates of youth violence in low-and middle-income countries: A meta-analysis.* 2019. **49**: p. 101306.

31. van der Merwe, A. and A. Dawes, *Youth violence: A review of risk factors, causal pathways and effective intervention.* Journal of Child and Adolescent Mental Health, 2007. **19**(2): p. 95-113.

32. World Health Organization. *World Health Statistics 2015*. 2015 [cited 2018 10/06/2018]; Available from: https://www.who.int/gho/publications/world_health_statistics/2015/en/.

33. World Health Organization, *Country Cooperation Strategy at a glance: Burkina Faso*. 2016.

34. Burkina Faso Mininstere de L'Economie des Finances et du Development Secretariat General, *Tableau de Bord Social du Burkina Faso*. 2017, INSTITUT NATIONAL DE LA STATISTIQUE ET DE LA DEMOGRAPHIE

35. Wirtz, A.L., et al., *Sexual violence, condom negotiation, and condom use in the context of sex work: results from two West African countries.* J Acquir Immune Defic Syndr, 2015. **68 Suppl 2**: p. S171-9.

36. Ouédraogo, S.Y.Y.A., E.J. Sisawo, and S.-L. Huang, *Sexual abuse and risky sexual behaviors among young female hawkers in Burkina Faso: a mixed method study.* BMC international health and human rights, 2017. **17**(1): p. 1.

37. Ismayilova, L., et al., *Maltreatment and mental health outcomes among ultra-poor children in Burkina Faso: A latent class analysis.* Plos One, 2016. **11**(10): p. e0164790.

38. Ismayilova, L. and L. Karimli, *Harsh parenting and violence against children: a trial with ultrapoor families in Francophone West Africa.* Journal of Clinical, Child and Adult psychology, 2020. **49**(1): p. 18-35.

39. Devries, K., et al., *Violence against children in Latin America and the Caribbean: What do available data reveal about prevalence and perpetrators?* Revista panamericana de salud publica, 2019. **43**: p. e66.

For peer review only - http://bmjopen.bmj.com/site/about/guidelines.xhtml

40. Farrington, D.P. and M.M. Ttofi, *Advancing Knowledge about Youth Violence: Child Maltreatment, Bullying, Dating Violence, and Intimate Partner Violence.* Journal of Family Violence, 2020. **36**(1): p. 109-115.

41. Sié, A., et al., *The Health and Demographic Surveillance System (HDSS) in Nouna, Burkina Faso, 1993-2007.* Glob Health Action, 2010. **3**(1).

42. Darling, A.M., et al., *Design and field methods of the ARISE Network Adolescent Health Study.* Journal of Tropical Medicine and International Health, 2020. **25**(1): p. 5-14.

43. Greis, A., et al., *Attitudes towards female genital cutting among adolescents in rural Burkina Faso: A multilevel analysis Female genital cutting in Burkina Faso.* Journal of Tropical Medicine and International Health, 2019. **25**(1): p. 119-131.

44. Bushman, B.J., et al., *Youth violence: What we know and what we need to know.* American Psychologist, 2016. **71**(1): p. 17.

45. Tripathi, V., *Youth violence and social media.* Journal of Social Sciences, 2017. **52**(1-3): p. 1-7.

46. Khandker, S.R., G.B. Koolwal, and H.A. Samad, *Handbook on impact evaluation: quantitative methods and practices*. 2010: World Bank Publications.

47. Gertler, P.J., et al., *Impact Evaluation in Practice*. Second ed. 2016, Washington D.C: International Bank for Reconstruction and Development/The World Bank.

48. Abramsky, T., et al., *What factors are associated with recent intimate partner violence? Findings from the WHO multi-country study on women's health and domestic violence.* BMC public health, 2011. **11**(1): p. 1-17.

49. Black, E., et al., *Prevalence and correlates of intimate partner violence against women in conflict affected northern Uganda: a cross-sectional study.* Confl Health, 2019. **13**: p. 35.

50. Fahlgren, M.K., et al., *Age and gender effects in recent violence perpetration.* Journal of interpersonal violence, 2020. **35**(17-18): p. 3513-3529.

For peer review only - http://bmjopen.bmj.com/site/about/guidelines.xhtml

51. Tashjian, S.M., J.L. Mullins, and A. Galván, *Bedtime autonomy and cellphone use influence sleep duration in adolescents.* Journal of Adolescent Health, 2019. **64**(1): p. 124-130.

52. Estrada-Martínez, L.M., et al., *Families, Neighborhood Socio-Demographic Factors, and Violent Behaviors among Latino, White, and Black Adolescents.* Youth & Society, 2011. **45**(2): p. 221-242.

53. Kellermann, A.L., et al., *Preventing youth violence: what works?* Annual review of public health, 1998. **19**(1): p. 271-292.

54. Botvin, G.J., K.W. Griffin, and T.D. Nichols, *Preventing youth violence and delinquency through a universal school-based prevention approach.* Prevention science, 2006. **7**(4): p. 403-408.

55. Lebrun-Harris, L.A., et al., *Bullying victimization and perpetration among US children and adolescents: 2016 National Survey of Children's Health.* Journal of Child and Family Studies, 2019. **28**(9): p. 2543-2557.

56. Aboagye, R.G., et al., *A multi-country analysis of the prevalence and factors associated with bullying victimisation among in-school adolescents in sub-Saharan Africa: evidence from the global school-based health survey.* BMC Psychiatry, 2021. **21**(1): p. 325.

57. Ashrafi, A., et al., *Bullying victimization among preadolescents in a community-based sample in Canada: a latent class analysis.* BMC research notes, 2020. **13**(1): p. 138.

58. Hokoda, A., H.-H.A. Lu, and M. Angeles, *School bullying in Taiwanese adolescents.* Journal of Emotional Abuse, 2006. **6**(4): p. 69-90.

59. Salmon, S., et al., *Bullying victimization experiences among middle and high school adolescents: Traditional bullying, discriminatory harassment, and cybervictimization.* Journal of Adolescence, 2018. **63**: p. 29-40.

For peer review only - http://bmjopen.bmj.com/site/about/guidelines.xhtml

60. Amegbor, P.M. and L. Pascoe, *Variations in Emotional, Sexual, and Physical Intimate Partner Violence Among Women in Uganda: A Multilevel Analysis.* J Interpers Violence, 2019. **36**(15-16): p. NP7868-NP7898.

61. Cofie, N., *A multilevel analysis of contextual risk factors for intimate partner violence in Ghana.* international Review of Victimology, 2020. **26**(1): p. 50-78.

62. Imai, K. and I.S. Kim, *When should we use unit fixed effects regression models for causal inference with longitudinal data?* American Journal of Political Science, 2019. **63**(2): p. 467-490.

For peer review only - http://bmjopen.bmj.com/site/about/guidelines.xhtml

Figure 1A. Age distribution among respondents

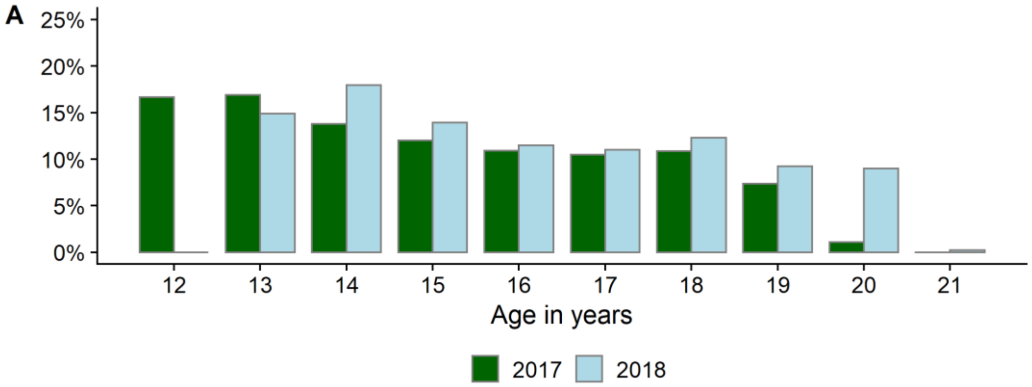

For peer review only

Figure 1. Distribution of years of education among respondents

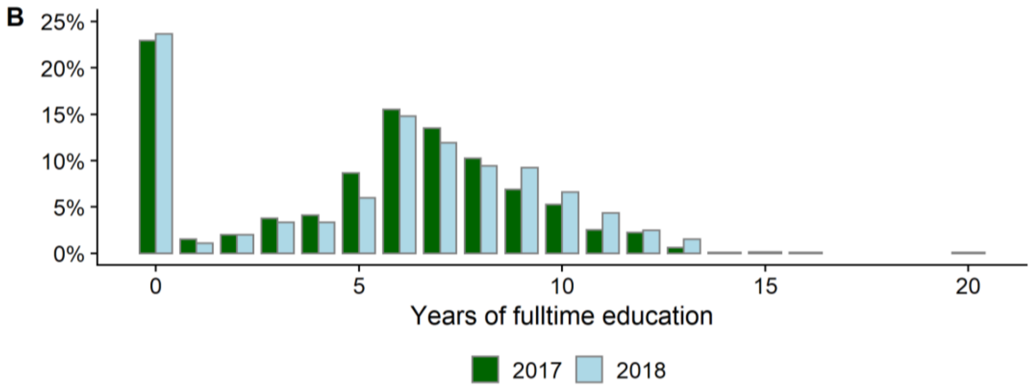

SUPPLEMENTARY MATERIAL

Title: The effects of education and age on experience of youth violence in a very low-income setting: a fixed-effects analysis from rural Burkina Faso

Supplementary Table 1: Variables and their measurements

	Measurement
Outcome	
Violence experience (VE1)	Ever experienced bullying, physical attack or unwelcome sexual advances in the last 6 months
Bullying	Yes / No
Physical attack	Yes / No
Sexual violence	Yes / No
Violence perpetration (VP1)	Ever perpetrated bullying or physical attack in the last 6 months
Bullying	Yes / No
Physical attack	Yes / No
Individual level factors	
Age	Age in complete years
Education	
Level of schooling	Primary, post-primary or secondary level
School attainment	Grade/class/standard
Years of schooling	Years of education completed in years
Highest level of schooling	Primary, post-primary, secondary, technical/vocational or university
Marital status	Single, engaged, married (monogamous), married (polygamous), civil union, separated/divorced or widowed
Experienced first sex	Had first sex in the last 6 months (Yes / No)
Multiple sex partners	Had sex with more than one person in your life time (Yes / No)
Number of sexual partners	Number of life time sexual partners
Employment	Done any work in the last 12 months to earn money or obtain food (Yes/ No)
Alcohol use	Ever drunk alcohol on your own (Yes / No)
Substance use	Ever used drugs (Yes / No)
Household level factors	
Household asset	Asset index for household
Household size	Number of persons living in household
Aged 0 – 14 years	
Aged 15 – 29 years	
Aged 30 – 44 years	
Aged 45 – 59 years	
Aged 60 and above	
Moving between household	Sometimes live in another household other than primary household (Yes / No)
Living with adult figure	Yes / No
Adult figure	Father / Mother
Age of adult figure	Age in complete years

For peer review only

Less than 30 years	
30 – 45 years	
46 – 60 years	
Above 60 years	
No adult figure	
Educational level adult figure	Primary, post-primary, secondary, technical/vocational or university
Male	
Female	
Occupation of adult figure	Farmer, merchant, teacher, other government worker, other (specified)
Male	
Female	
Frequency of parental /guardian support	In the last 30 days how often did parent/ guardian……
With important life decisions	Helped you make important decisions (never, rarely, sometimes, most of the times or always)
With understanding problems	Understand your problems and worries (never, rarely, sometimes, most of the times or always)
Talking about what happens	Take time to talk with you about things that happen to you (never, rarely, sometimes, most of the times or always)
Paying attention to opinion	Pay attention to your opinion (never, rarely, sometimes, most of the times or always)
Media use	
Access to media	
Radio	Yes / No
TV	Yes / No
Magazines	Yes / No
Use of media	
Radio	Average number of hours per day spent listening to radio
TV_1	Frequency of watching TV [never, rarely (some hours per month), often (some hours per week) or very frequently (several hours per day)]
TV_2	Average number of hour per day spent watching TV
Magazines	How often do you read magazines [never, rarely (some hours per month), often (some hours per week) or very frequently (several hours per day)]
Community level factor	
Access to education	Community share of school enrolment

For peer review only

Supplementary Table 2: Parental support (n=1291)

Variable	2017		2018	
	Frequency	Percent	Frequency	Percent
Parents understand worries				
Never	299	23.27	68	5.29
Rarely	201	15.64	193	15.01
Sometimes	271	21.09	391	30.40
Most of the time	159	12.37	295	22.94
Always	351	27.32	339	26.36
Missing	4	0.31		
Parents help make decisions				
Never	212	16.50	27	2.10
Rarely	257	20.00	193	15.01
Sometimes	311	24.20	350	27.22
Most of the time	159	12.37	331	25.74
Always	337	26.23	384	29.86
Missing	9	0.70	1	0.08
Parents talk about things that happen				
Never	299	23.27	83	6.45
Rarely	253	19.69	268	20.84
Sometimes	324	25.21	333	25.89
Most of the time	161	12.53	340	26.44
Always	243	18.91	262	20.37
Missing	5	0.39		
Parents pay attention to what you say				
Never	317	24.67	94	7.31
Rarely	296	23.04	225	17.50
Sometimes	381	29.65	416	32.35
Most of the time	133	10.35	236	18.35
Always	153	11.91	314	24.42
Missing	5	0.39	1	0.08

We built a summative score of parental support by adding the four variables, with a minimum score of 0 and a maximum score of 16.

For peer review only

Supplementary Table 3: Full regression models predicting violence experiences

Variables	Bullying		Physical Attacks	
	Model 2 Adjusted	Model 3 Adjusted & interaction	Model 2 Adjusted	Model 3 Adjusted & interaction
Interview 2 vs interview 1	-0.42[-0.75,-0.08]	-0.44 [-0.78,-0.11]	-0.10[-0.27,0.07]	-0.09[-0.26,0.08]
Marital status (vs other)				
Single	-0.20[-1.03,0.63]	-0.24[-1.07, 0.59]	0.03[-0.40,0.46]	0.05[-0.38,0.48]
Ever worked	0.01[-0.27,0.28]	0.01[-0.27,0.28]	0.05[-0.09,0.20]	0.05[-0.09,0.19]
Number of people in the household	-0.04 [-0.07,-0.02]	-0.04 [-0.07,-0.02]	-0.01[-0.02,0.00]	-0.01[-0.02,0.00]
Mother is alive	0.74[-1.34, 2.82]	0.77[-1.31,2.85]	-0.30[-1.38,0.77]	-0.32[-1.40,0.76]
Father is alive	1.12[-2.41,0.17]	-1.11[-2.41, 0.18]	-0.35[-0.99,0.28]	-0.34[-0.98,0.29]
Lives with Mother	-0.12[-0.74,0.51]	-0.15[-0.77,0.48]	0.06[-0.26,0.39]	0.08[-0.25,0.40]
Lives with father	0.46[-0.18,1.10]	0.45[-0.18,1.09]	-0.13[-0.46,0.20]	-0.12[-0.45,0.21]
Lives alone	-0.17[-1.03,0.69]	-0.14[-1.00,0.72]	-0.38[-0.83,0.07]	-0.40[-0.84,0.05]
Parental support	-0.18 [-0.31,-0.05]	-0.18 [-0.31,-0.05]	-0.04[-0.11,0.03]	-0.04[-0.11,0.03]
Household wealth quintile				
Lowest (base)				
2	0.08[-0.34,0.50-]	0.06[-0.36,0.49]	-0.05[-0.27,0.17]	-0.04[-0.26,0.17]
3	-0.01[-0.44,0.42]	-0.02[-0.44,0.41]	-0.06[-0.28,0.16]	-0.06[-0.28,0.16]
4	-0.04[-0.52,0.44]	-0.07[-0.55,0.41]	-0.08[-0.32,0.17]	-0.06[-0.31,0.18]

For peer review only

Highest	0.43[-0.15,1.00]	0.40[-0.18,0.98]	0.12[-0.18,0.41]	0.13[-0.17,0.42]
Has own bedroom	-0.08[0.47,0.32]	-0.08[-0.47,0.32]	-0.01[-0.21,0.20]	-0.01[-0.21,0.20]
Radio hours/day	0.13[-0.01,0.27]	0.13[-0.00,0.27]	-0.02[-0.09,0.05]	-0.02[-0.09,0.05]
Frequency of watching television				
Never (base)				
Rarely (some hours per month)	-0.34[0.72,0.03]	-0.29[-0.67,0.09]	0.06[-0.13,0.26]	0.03[-0.16,0.23]
Often (several hours per week)	-0.11[-0.46,0.24]	-0.07[-0.42,0.29]	-0.04[-0.22,0.14]	-0.06-0.24,0.12]
Very often (several hours per day)	0.63 [0.14,1.11]	0.63 [0.15,1.12]	-0.19[00.44,0.06]	-0.19[-0.44,0.06]
Frequency of reading magazines				
Never (base)				
Rarely (some hours per month)	1.01 [0.46,1.57]	0.99 [0.43,1.54]	-0.18[-0.47,0.11]	-0.17[-0.46,0.12]
Often (several hours per week)	1.12 [0.38,1.85]	1.08 [0.35,1.81]	-0.22[-0.61,0.16]	-0.21[-0.59,0.17]
Very often (several hours per day)	-0.17[-1.06,0.73]	-0.07[-0.97,0.83]	0.90 [0.44,1.36]	0.85 [0.38,1.31]
Household size missing	-1.77[-3.73, 0.20]	-1.68[-3.64, 0.29]	-0.46[-1.39,0.46]	-0.50[-1.43,0.43]
Freq. of reading magazine missing	6.99 [4.23, 9.75]	6.93 [4.16,9.69]	0.46[-0.97,1.89]	0.49[-0.94,1.92]

All models present coefficients and 95% confidence intervals. All models contain fixed effects for each respondent. Age centred at 15.

For peer review only

BMJ Open

The effects of education and age on the experience of youth violence in a very low-resource setting: a fixed-effects analysis in rural Burkina Faso.

Journal:	*BMJ Open*
Manuscript ID	bmjopen-2022-071104.R1
Article Type:	Original research
Date Submitted by the Author:	19-Jul-2023
Complete List of Authors:	Kuunibe, Naasegnibe; Simon Diedong Dombo University of Business and Integrated Development Studies,<br>Bountogo, Mamadou; Centre de Recherche en Sante de Nouna, Nouna; Université de Ouagadougou, Ouaga<br>Ouermi, Lucienne; Centre de Recherche en Sante de Nouna<br>Sié, Ali; Centre de Recherche en Sante de Nouna Nouna, Boucle du Mouhoun, BF<br>Bärnighausen, Till ; Heidelberg University, Heidelberg Institute of Global Health<br>Harling, Guy; University College London, Institute for Global Health; Harvard University T H Chan School of Public Health, Department of Epidemiology
**Primary Subject Heading**:	Public health
Secondary Subject Heading:	Public health
Keywords:	HEALTH ECONOMICS, PUBLIC HEALTH, STATISTICS & RESEARCH METHODS, Adolescent

SCHOLARONE™
Manuscripts

I, the Submitting Author has the right to grant and does grant on behalf of all authors of the Work (as defined in the below author licence), an exclusive licence and/or a non-exclusive licence for contributions from authors who are: i) UK Crown employees; ii) where BMJ has agreed a CC-BY licence shall apply, and/or iii) in accordance with the terms applicable for US Federal Government officers or employees acting as part of their official duties; on a worldwide, perpetual, irrevocable, royalty-free basis to BMJ Publishing Group Ltd ("BMJ") its licensees and where the relevant Journal is co-owned by BMJ to the co-owners of the Journal, to publish the Work in this journal and any other BMJ products and to exploit all rights, as set out in our licence.

The Submitting Author accepts and understands that any supply made under these terms is made by BMJ to the Submitting Author unless you are acting as an employee on behalf of your employer or a postgraduate student of an affiliated institution which is paying any applicable article publishing charge ("APC") for Open Access articles. Where the Submitting Author wishes to make the Work available on an Open Access basis (and intends to pay the relevant APC), the terms of reuse of such Open Access shall be governed by a Creative Commons licence – details of these licences and which Creative Commons licence will apply to this Work are set out in our licence referred to above.

Other than as permitted in any relevant BMJ Author's Self Archiving Policies, I confirm this Work has not been accepted for publication elsewhere, is not being considered for publication elsewhere and does not duplicate material already published. I confirm all authors consent to publication of this Work and authorise the granting of this licence.

For peer review only

The effects of education and age on the experience of youth violence in a very low-resource setting: a fixed-effects analysis in rural Burkina Faso.

Naasegnibe Kuunibe[1,2*], Mamadou Bountogo[3], Lucienne Ouermi[3], Ali Sié[3], Till Bärnighausen[2,4,5,], Guy Harling[4,6,7,8,9]

* Corresponding Author:

P O Box WA64, Wa, Upper West Region Ghana

nkuunibe@ubids.edu.gh / +233(0)209006165.

1. Department of Economics, Faculty of Social Science and Arts, SD-Dombo University of Business and Integrated Development Studies, Wa, Ghana
2. Heidelberg Institute for Global Health, Heidelberg University Hospital, Heidelberg, Germany
3. Centre de Recherche en Santé de Nouna, Nouna, Burkina Faso
4. Africa Health Research Institute, KwaZulu-Natal, South Africa
5. Department of Global Health and Population, Harvard T.H. Chan School of Public Health, Boston, MA, USA
6. Institute for Global Health, University College London, London, United Kingdom
7. MRC/Wits Rural Public Health & Health Transitions Research Unit (Agincourt), University of the Witwatersrand, South Africa
8. Department of Epidemiology & Harvard Center for Population and Development Studies, Harvard T.H. Chan School of Public Health, Boston, MA, USA
9. School of Nursing & Public Health, College of Health Sciences, University of KwaZulu-Natal, KwaZulu-Natal, South Africa

Keywords: Adolescence, physical violence, bullying, panel fixed-effects, Burkina Faso.

Word count: 3679

ABSTRACT

Youth violence is both a public health and an economic problem. Cross-sectional evidence from low- and middle-income settings suggests individual-level factors influence adolescent

For peer review only - http://bmjopen.bmj.com/site/about/guidelines.xhtml

violence experience. However, longitudinal evidence on the effect of age and education on youth violence in such settings is limited, especially where educational attainment is low.

Methods: We analysed two waves of data from 1644 adolescents in north-western Burkina Faso from 2017 and 2018. We examined experience of physical attack in the past 12 months and of bullying in the past 30 days. Our exposures were completed years of age and educational attainment. We used panel fixed-effects OLS regression models to analyse the associations of interest adjusting for individual- and household-level time-varying covariates and tested for effect-modification by gender.

Results: A substantial minority of respondents experienced violence in both waves (24.1% bullying and 12.2% physical attack), with males experiencing more violence. Bullying was positively associated with more education (β=0.12; 95% confidence interval [CI]: 0.02, 0.22) and non-significantly with older age. Both effects were stronger in males than females, although the gender differences were not significant. Physical attacks fell with increasing age (β=-0.18; 95% confidence interval [CI]: -0.31, -0.05) and this association was again stronger in males than females; education and physical attacks were not substantively associated.

Conclusions: Bullying and physical attacks are common for rural adolescent Burkinabe. The age patterns found suggest that, particularly for males, there is a need to target violence prevention at younger ages and bullying prevention at slightly older ones, particularly for those remaining in school. Nevertheless, a fuller understanding of the mechanisms behind our findings are needed to design effective interventions to protect youth in low-income settings from violence.

INTRODUCTION

Violence globally represents both public health and an economic problem. In health terms, violence generates both mortality and morbidity. Violence is estimated to cause 1.3 million

For peer review only - http://bmjopen.bmj.com/site/about/guidelines.xhtml

deaths annually, accounting for 2.5% of global mortality,[1]. Violence often requires acute health service access (e.g., assault victims requiring emergency hospital care) and can result in long-term physical disability, depression or reproductive health problems,[1, 2]. Violence also affects local economies through workforce absenteeism, loss of productivity and loss of human capital. Families can fall into poverty if a breadwinner dies or becomes permanently disabled due to violence,[1, 3].

Youth violence – which includes bullying, physical fighting, sexual and physical assault and homicide – is particularly problematic because it generates higher economic, welfare and criminal justice costs,[4]. Besides death, injury and psychological harm, youth violence can lead to increased subsequent health risks behaviours such as smoking, substance abuse, unsafe sex and further violence,[5, 6]. An estimated 200,000 youth homicides occur each year, 83% among males, nearly all in low- and middle-income countries (LMICs), with particularly high rates in Latin America and the Caribbean (LAC) and sub-Saharan Africa [4]. In sub-Saharan Africa in particular, historical, economic and social factors continue to expose youth to violence,[7, 8]. Although violence affects all youth, adolescent girls and young women (AGYW) in LMICs appear to be most affected,[9-11]. Social and cultural norms, such as arranged and teenage marriage and denial of resource access to women, often keep AGYW economically dependent on males and thus vulnerable to abuse, especially from intimate partners,[12-14]. AGYW experience all forms of violence (emotional, physical and sexual), perpetrated both by men and often other females in domestic and social settings [15-18].

Many factors have been proposed as determinants of youth violence receipt and perpetration in LMICs, often adopting or adapting Heise's integrated ecological framework for violence against women,[19, 20]. This framework conceptualizes violence as a multifaceted phenomenon grounded in an interplay among personal, situational and sociocultural factors.

For peer review only - http://bmjopen.bmj.com/site/about/guidelines.xhtml

We modified this conceptualization to cover all forms of adolescent violence, concentrating on individual-level and microsystem (family/household/relationship) factors given our focus within a specific geography. We focus on two individual-level factors believed to play an important role in determining violence experience: age and education. Given the magnitude of youth violence, evidence of associations between violence, age and educational attainment, and calls for youth violence interventions,[1, 2], causal evidence on whether policies based on age or education are likely to affect violence levels is important. Past longitudinal analyses of adolescent violence often focused on the consequences of violence experience, rather than predictors of violence itself,[21, 22]. We instead focus on predictors of adolescent violence experience, to contribute to the upstream prevention of violence, rather than efforts to break the connection between violence and its sequelae.

Age is strongly associated with both the experience and perpetuation of violence,[3, 23]. Youth aged 15 to 29 are more likely to both experience and perpetrate violence than older adults, often experiencing violence perpetrated by their older peers or family members,[3, 23]. Males are more likely to perpetrate violence, while young girls and women are more likely to experience it,[9, 10, 14, 24, 25]. However, evidence on the causal effect of age on violence is comparatively scarce, with few even cross-sectional studies explicitly focused on youth,[3, 11, 26].

Education is theorized to protect against violence, since more-educated persons are less likely to either perpetrate or experience violence,[26, 27]. Evidence shows that women without education were 5.6 times more likely than those with college education to experience intimate partner violence (IPV). Similarly, wives of uneducated men were 1.84 times more likely than those whose husbands had college education to experience IPV. Even at the community level, the likelihood of IPV declined as community male and female literacy increased (after

For peer review only - http://bmjopen.bmj.com/site/about/guidelines.xhtml

controlling for individual level factors) [28]. However, causal evidence on the effect of education on youth violence in sub-Saharan Africa is again limited. Two studies have used changes in national school policy as natural experiments in this context. One focused on violence, finding that a one-year increase in grade attainment was associated to a nine percentage-point reduction in the probability of experiencing sexual violence in Uganda, but no significant effect in Malawi,[27]. A second focused on sexual health, finding an additional year of schooling was associated with 0.11 fewer births and 14 percentage points less teen marriage in Ghana,[29]. Both of these studies necessarily assess the overall impact of policy change, rather than the increase in education alone, and it is unclear how their findings extrapolate to lower educational attainment settings.

Burkina Faso is a landlocked country in West Africa, which despite economic and political reforms remains one of the poorest in the world, with about half of its population living below the international poverty line,[30]. Economic deprivation is strongly associated with youth violence,[7, 31, 32]. The country is very young, with around 45% aged under fifteen and a further 20% aged 15-24 in 2015,[30, 33]. Educational access and youth literacy are limited, with only 13% of adults having completed primary education,[33, 34]. Within Burkina Faso, poverty is highest in the Boucle du Mouhoun region,[35]. Violence experience is common for young Burkinabe, with lifetime physical violence prevalence reported at 47-80% and sexual violence at 33-51%,[36, 37]. Adverse psychological and mental health outcomes commonly follow such experiences,[38, 39]. However, studies of Burkinabe youth violence have generally used cross-sectional designs and have not explored the effects of education or age specifically,[3, 40].

We therefore analysed longitudinal data on adolescents in Boucle de Mouhoun to assess the effects of age and education on violence experience. The potential for educational interventions

For peer review only - http://bmjopen.bmj.com/site/about/guidelines.xhtml

to have violence-specific benefits in such high-poverty, low-education settings is likely to be substantial,[38, 39]. By using fixed-effects analysis we were able to exclude time-specific and time-invariant confounders, something particularly important given the many unobserved predictors of violence perpetration and victimization,[22, 41].

METHODS

Study design

We used data from the Nouna Health and Demographic Surveillance Site (HDSS) in north western Burkina Faso, which has been gathering demographic and epidemiological health information data since 1992. The 59 villages and the town of Nouna that comprise the HDSS have a population of slightly over 100,000 individuals and include one hospital and 13 primary health centres (CSPS),[42].

Our study used longitudinal data from two Burkina Faso waves of the Africa Research, Implementation Science and Education (ARISE) adolescent health study,[43], collected in the Nouna HDSS in 2017 and 2018. Data were collected from 1644 adolescents aged 12-20 in 2017, based on a stratified random sample of 2544 age-eligible residents in Nouna town and ten villages,[43, 44]. A follow-up round was conducted in 2018, attempting to contact all those who participated in 2017; 1291 interviews were completed. In both years a standardized questionnaire was used to collect self-reported information on socio-demographics, health practices, health outcomes and risk factors. Data were collected by field staff with background in public health, medicine or a related field who had experience in conducting research and had general knowledge about local culture, health issues and the population under study. All study staff received in-depth training at the beginning of the study, covering the topic of research, human research ethics, the study protocol, questionnaire modules, electronic data entry and the procedures for implementing the study, including anthropometric evaluation.

For peer review only - http://bmjopen.bmj.com/site/about/guidelines.xhtml

Patient and Public Involvement

No patient involved.

Measures

We used two primary outcomes of youth violence, both captured as count variables: experience of physical attack in the past 12 months; and experience of bullying in the past 30 days (where bullying was defined as physical attacks, threats, insults, frequent nasty teasing, being left out on purpose or having rumors passed about them). We also generated binary measures of any experience for each outcome, in alignment with the World Report of Violence and Health's definitions,[2]. Our exposures were age (in completed years) and education (years of fulltime education).

We additionally considered a range of time-varying covariates at the individual and household levels (based on Heise [20] framework,[32]), plus media use,[45, 46]. Specifically, our individual-level covariates were: currently in school; marital status (never married vs all else); any work in the past 12 months; and sexual behaviour (sexual debut and number of sexual partners). Our household-level covariates were: household size; household wealth quintile; parental vital status; parental support level (16 point scale, converted to the first principal component of the four variables included); parental co-residence (respondent lives with both parents, only mother, only father or lives alone); respondent has their own bedroom. Our media covariates were: any access to television; and frequency of watching television or reading magazines (never, rarely, often, very often). Full variable definitions are provided in Supplementary Table 1 and Supplementary Table 2.

For peer review only - http://bmjopen.bmj.com/site/about/guidelines.xhtml

Statistical analyses

We first described our data using frequency and percentages in both waves, including a comparison of those lost-to-follow-up vs those completing both waves. We then dropped any respondents who were missing data for the outcome variables, either due to preferring not to respond, not know their answer or where fieldwork errors affected responses, while for some the question was not applicable.

When considering the causal effect of education and age on youth violence, a major concern is unobserved confounding. Given the difficulty of implementing randomized controlled trials, since age is not directly manipulatable and intentional exposure to violence unethical,[47, 48], we exploited the panel nature of the data structure to run fixed-effects regression models to remove all time-invariant confounding. We specified our model as:

$$y_{it} = \alpha_i + \beta X_{it} + \gamma Z_{it} + \delta_t + \rho_i \qquad (1)$$

Where y_{it} is youth violence for each individual i at each time point t, X_{it} represents our time-varying exposures (education and age), Z_{it} is other time-varying factors for each individual, δ_t is a period-specific fixed effect to capture all individual-invariant factors and ρ_i are individual-specific fixed-effects which capture all time-invariant factors for each individual, e.g., gender, ethnicity, underlying proclivity to violence.

For each outcome (bullying and physical attack), we implemented three linear regression models of the count of reported events, i.e., assuming an observation-specific error structure $\epsilon_{it} \sim N(0,\sigma^2)$. We attempted to use Poisson and Negative Binomial models, i.e., modelling y_{it} as count data using a log-link and assuming that the variance of y_{it} is either equal to its mean (Poisson) or its mean plus a dispersion term (Negative Binomial), however neither model converged.

For peer review only - http://bmjopen.bmj.com/site/about/guidelines.xhtml

Model 1 considered only mean-centred age and years of full-time education. In model 2, we add all time-varying covariates. In model 3, we included interaction terms for gender with age and education to identify any gender-specific effects.

RESULTS

Description of sample

At baseline in 2017, 1,644 young people were interviewed, of whom 948 (57.7%) were male. By 2018, 21.5% of respondents, comprising 167 (24.0%) females and 186 (19.6%) males, were lost to follow up, leaving 1,291 respondents who participated in both rounds. We dropped 32 individuals (64 data points) who had missing values for the question on bullying, leaving 1,258 respondents for the bulling analysis. Similarly, we dropped 14 individuals (28 data points) who did not answer physical attack question to arrive at 1,276 respondents. Figure 1 provides a flow chart of how the data were managed. We compared those who were and were not lost to follow up (Supplementary Table 3) and found only one significant difference with those who were retained, those not reinterviewed were less likely to be in school and had lower school attainment in wave 1.

[Figure 1 about Here]

The 1,291 respondents were aged 12 to 20 years in 2017: median 15.5, interquartile range (IQR): 14-18 (2A). Around half were enrolled in school at each interview: 703 (54.5%) in 2017; 671 (52.0%) in 2018 (2B).

[Figure 2A about Here]

[[Figure 2B about Here]

Around one-quarter of respondents never completed a year of fulltime education, while most others had at most attended primary or post-primary level. Over 90% of respondents were

For peer review only - http://bmjopen.bmj.com/site/about/guidelines.xhtml

single at both interviews, and less than 20% in both 2017 and 2018 reported ever having sexual intercourse (Table 1). The proportion of respondents who ever worked fell from over 60% in 2017 to under 42% in 2018. Most respondents had living fathers (over 97%) and mothers (over 91%), however around one-quarter did not live with their parents. Media access was mixed: about 20% had no access to television in 2017 but around 15% watched several hours a day (access dropped by 2018); magazine reading was rare. Household wealth was by design evenly distributed across wealth quintiles. Households were large, with a median of 8 or 9 members.

Table 1. Descriptive statistics of independent variables (in percent)

	Bullying sample		Physical attacks sample	
	**2017**	**2018**	**2017**	**2018**
**N**	1253		1271	
Marital status				
Single vs. all other	90.7	89.6	90.8	89.6
Living situation				
Ever worked vs never	62.4	42.1	61.4	41.7
Mother is alive	97.6	96.8	97.7	97.0
Father is alive	91.7	89.7	91.9	89.8
Lives with mother	78.0	80.1	77.7	80.3
Lives with father	74.8	76.1	74.6	76.1
Lives alone	4.5	0.2	4.4	0.2
Has own bedroom	18.0	19.9	17.8	19.7
Household wealth quintile				
Lowest	19.0	20.8	19.8	21.0
Second lowest	19.5	20.4	20.0	20.7
Middle	22.4	19.0	22.1	19.0
Second highest	18.4	20.1	17.9	20.2
Highest	20.8	19.6	20.2	19.1
Education				
Currently in school	54.0	51.6	54.6	52.1
Highest school level				
None	46.1	48.4	45.5	47.9
Primary (1 – 6)	20.3	10.8	20.8	11.3
Post-primary (7 – 10)	29.9	33.6	30.1	33.7
Secondary (1 – 3)	2.6	3.9	2.6	3.9
University		0.1		0.1
Not applicable	1.1	3.2	1.1	3.2
Media use				
Has access to television	80.5	70.6	80.1	70.3
Frequency of watching television				
Never	20.1	30.8	20.5	31.0
Rarely (some hours per month)	22.8	22.8	22.7	22.8
Often (several hours per week)	42.1	36.2	41.8	36.1

For peer review only - http://bmjopen.bmj.com/site/about/guidelines.xhtml

Very often (several hours per day)	15.1	10.1	15.1	10.2
Frequency of reading magazines				
Never	90.9	87.1	91.1	87.3
Rarely (some hours per month)	2.4	8.5	2.23	8.4
Often (several hours per week)	2.4	4.1	2.4	3.9
Very often (several hours per day)	4.3	0.3	4.3	0.3
Sexual behaviour				
Ever had intercourse	17.3	19.4	15.8	18.9
Number of lifetime sexual partners				
1	9.7	13.5	9.4	13.3
2 – 7	3.8	5.2	3.8	5.3
8 – 17	0.3		0.3	
No response	2.1	1.3	2.4	0.3
Household size †	9 [6, 12]	8.5 [7, 11]	8.5 [6, 12]	8 [7, 11]
Parental support†	7 [4, 12]	10 [7, 13]	7 [4, 11]	10 [7, 13]

† depict medians and interquartile ranges. Samples are those with non-missing outcome responses for each of the two measures.

A substantial minority of respondents experienced bullying and physical attacks (Table 2). Overall, 189 females (18.9%) and 416 males (27.6%) experienced bullying in the 30 days preceding the interview, while 111 females (10.6%) and 199 males (13.2%) experienced physical attacks in the preceding 12 months. Across both rounds, males experienced both more violence than females: males experienced 416 of 605 (68.8%) unique bullying instances in the past 30 days, and 199 of 310 (64.2%) unique violence instances in the past 12 months. Bullying experience declined slightly with age – from over 26% among under 15s to 21% among over 18s; physical attacks fell more sharply, especially in early adolescence.

Table 2: Distribution of violence experience across observations by age and gender

Variable	N	Bullying (%)	N	Physical attacks (%)
Gender				
Female	996	189 (18.9)	1,038	111 (10.6)
Male	1,510	416 (27.6)	1,504	199 (13.2)
Age				
12	19	52 (26.1)	210	51 (24.3)
13	387	100 (25.8)	398	73 (18.3)
14	389	104 (26.7)	403	65 (16.1)
15	327	76 (23.2)	331	30 (9.1)
16	286	67 (23.4)	288	22 (7.6)
17	274	64 (23.4)	272	27 (9.9)
18	297	63 (21.2)	295	20 (6.8)
19	214	43 (20.1)	212	15 (7.1)

For peer review only

For peer review only - http://bmjopen.bmj.com/site/about/guidelines.xhtml

20	130	35 (26.9)	130	7 (5.4)
21	3	1 (33.3)	3	0 (0.0)
Total	2506	605 (23.8)	2542	310 (12.2)

Note: Each individual is represented twice in this table, once per survey round.

Fixed-effects analysis

In bivariate fixed-effects regression, older age was associated with non-significantly more bullying (0.14 more bullying experiences per month for each additional year of age, 95% confidence interval [CI]: -0.12, 0.39) and associated with significantly fewer physical attacks (-0.19, 95%CI: -0.32, -0.06). More education was associated with significantly more bullying (0.11 bullying experiences per month for each additional year of schooling, 95%CI: 0.01, 0.21), but not with physical attacks (Table 3, Model 1). Controlling for time-varying potential confounders attenuated the association of age and bullying but otherwise had limited effects on our relationship of interest (Table 3, Model 2). When we allowed effects to vary by gender (Table 3, Model 2), associations in all four models were more positive for men than for women, with wider gaps for the impact of age on both outcomes than for education. All these results were independent of a substantial but imprecise negative association between currently being in school and bullying or attacks.

For peer review only

For peer review only - http://bmjopen.bmj.com/site/about/guidelines.xhtml

Table 3: OLS models predicting count of violence events

	Model 1<br><br>Unadjusted	Model 2<br><br>Adjusted	Model 3<br>Adjusted &<br>interaction	Interaction test
Bullying in the last 30 days (N=2506)				
Age in years	0.14 [-0.12, 0.39]	0.08 [-0.17, 0.33]		
Female			-0.08 [-0.39, 0.24]	
Male			0.18 [-0.10, 0.45]	F=2.76, p=0.10
Fulltime education in years	0.11 [0.01, 0.21]	0.12 [0.02, 0.22]		
Female			0.07 [-0.11, 0.26]	
Male			0.14 [0.03, 0.26]	F=0.39, p=0.53
Currently in school		-0.43 [-1.05, 0.19]	-0.41 [-1.03, 0.21]	
Physical attacks in the last 12 months (N=2542)				
Age in years	-0.19 [-0.32, -0.06]	-0.18 [-0.31, -0.05]		
Female			-0.10 [-0.26, 0.05]	
Male			-0.23 [-0.38, -0.09]	F=2.74, p=0.10
Fulltime education in years	0.02 [-0.03, 0.07]	0.04 [-0.01, 0.09]		
Female			0.06 [-0.03, 0.16]	
Male			0.03 [-0.03, 0.09]	F=0.41, p=0.52
Currently in school		-0.27 [-0.59, 0.05]	-0.27 [-0.59, 0.04]	

The table presents four regression models per column, all showing point estimates and 95% confidence intervals. All models contain fixed-effects for each respondent and interview round. Age centred at 15. Models 2 and 3 are also adjusted for marital status, ever worked, household size, parent alive, living with parents, household wealth, has own bedroom and access to media; full results available in Supplementary Table 4.

DISCUSSION

In this study we employed individual and time fixed-effects models to assess the effects of age and education on violence experience in the form of bullying and physical attacks among adolescents and young adults in a panel study in rural Burkina Faso. We found bullying experience prevalence in the past 30 days ranging from 20-30%, and physical attack experience of 10-15% in the previous 12 months. While there is little directly comparable data in Burkina Faso, both levels seem concerning, if in line with studies elsewhere,[3, 40]. In fixed-effects models we found bullying was associated with more education and weakly with greater age, both effects stronger in males, while physical attacks were associated with younger age (again more strongly for males) but not with education.

For peer review only - http://bmjopen.bmj.com/site/about/guidelines.xhtml

Our findings on the effect of age on youth experience of physical attacks are consistent with some observational evidence elsewhere, for example physical violence from both peers and caregivers falls with age in Latin America and the Caribbean,[40]. Evidence on the causal effect of age on violence is rare, with even cross-sectional studies focused on youth uncommon,[3, 11, 26]. Studies of intimate partner violence (IPV) amongst women suggest rates are higher in older teenage girls compared to adults,[49, 50], but this does not allow within-adolescence comparisons. The faster decline with age that we see for males (from a higher initial level) is important to note: while criminal interpersonal violence appears peaks around age 18 in many settings, our findings and past work suggest that overall frequency of violence experience may in fact decline across teenage years, at least in non-urban settings,[51]. In combination, this evidence suggests a shift in violence experience composition for adolescent males that would be worth further investigation.

The implications of this negative association between age and violence experience in adolescence depend on what mechanisms are generating them. First, age might be a proxy for predictors of violence that we have not captured in this analysis. This might include adolescent autonomy in decision-making, for example relating to bedtime and the amount and type of television watched, which typically rises with age,[52]: adolescent autonomy was negatively associated with youth violence among US Latino youth,[53]. Alternatively, age might be a distal determinant of factors leading more directly to violence experience. For example, older adolescents may be better able to protect themselves against aggressive behaviour from their peers or adults. If this is the case, then structural interventions (at the family, community or national levels) or behaviour change interventions (e.g., teaching adolescents how to avoid confrontations) might be beneficial in protecting younger adolescents,[54, 55]. Further research to understand these causal mechanisms, and thus design effective interventions, will

For peer review only - http://bmjopen.bmj.com/site/about/guidelines.xhtml

need to include more detailed quantitative data on who perpetrates violence against younger adolescents and qualitative information on how and why such violence comes about.

Our finding that bullying increases with age contradicts some past research. Observationally, bullying victimization rates are higher in younger children than in older adolescents, both in the US and in sub-Saharan Africa,[56, 57]. Our results may reflect the stronger control our fixed-effects approach provides against between-individual and temporal confounding, suggesting that the decline in bullying seen elsewhere is a function of factors associted with age, rather than age itself. Our finding of a stronger association for males adds to a mixed literature, aligning with studies from Taiwan and Saskatchwan Canada,[58, 59], but in contrast to findings from the US and Manitoba Canada,[58, 60].

We found that education was not associated with violence experience in our setting. A similar null effect of education (grade attainment) was reported for 19-31 year old Malawian women,[27]. A recent meta-analysis of 86 studies in 60 LMICs noted that poor academic performance and weak school attachment were correlated with increased youth violence,[23], in contrast to our null findings. Again, more detail is available for IPV, with a Ugandan study finding that less-educated women were more likely to experience physical IPV – however this study included women aged 15-49, which makes direct comparison difficult,[61]. Other studies have confirmed the protective effect of education on violence in different settings,[49, 62]. The discrepancy between others' findings and ours may reflect the majority of past studies being cross-sectional, while we were able to use panel data. It may also reflect different exposures, since we considered quantity (years of schooling) rather than quality (performance or attachment).

Past evidence on the effect of education on bullying is mixed, with several studies finding lower bullying amongst those with more education,[57, 58] and few finding the opposite,[60].

For peer review only - http://bmjopen.bmj.com/site/about/guidelines.xhtml

Our finding of a positive association between education and bullying in both males and females may reflect the a true causal association, or the residual effect of being in school – given the opportunities that this provides for bullying relative to the alternative settings of field-based work or animal herding.

Finally, our analysis covers a population where half of adolescents are not now, and one-quarter never have been, attending school. The role of education in promoting or protecting against violence at the community level may be different in settings where education is not even close to universal. Further investigation of why our results looks different from other settings, including qualitative study of social norms surrounding violence across levels of educational attainment, would be instructional.

Strengths and limitations

A key strength of our study is the fixed-effects design which robustly removes time-invariant confounding of effect measures,[63]. Nevertheless, our study also has potential limitations: while fixed-effects analyses are powerful, they cannot control for unmeasured time-varying confounding and we may have therefore not fully accounted for factors that change rapidly among adolescents, such as increased social media access or social network change. The use of self-reported data may have led to reporting biases, although these would have had to vary differentially over time within respondents in order to bias our fixed-effects analyses. Generalizability from a sample drawn from a single district is always difficult to assess. However, by using a population-based sampling frame our analysis provides results that represent the entire local population and are likely to be broadly applicable in poor, rural settings in Burkina Faso and beyond.

For peer review only - http://bmjopen.bmj.com/site/about/guidelines.xhtml

Conclusion

A substantial minority of adolescents in rural north-western Burkina Faso report recent experiences of bullying or physical attack. We hypothesised years of education received and age would be associated with violence experience in Burkina Faso. Our findings show the prevalence of these experiences was not significantly associated with years of education received, even within individual respondents, but did fall with age. However, our study was not able to identify mechanisms behind these associations, and we therefore recommend a mixed method study that includes study of household dynamics to move beyond an individualized understanding of violence amongst adolescents. Such an understanding is central to designing interventions to better protect youth in low-income settings from violence.

For peer review only

Contributorship statement

NK: Conceptualization, Methodology, Formal analysis, Writing – original draft. **MB**: Investigation, Writing – Review & Editing, Project administration. **LO**: Investigation, Writing – Review & Editing, Project administration. **AS**: Investigation, Resources, Writing – Review & Editing, Supervision. **TB**: Conceptualization, Methodology, Writing – Review & Editing, Project administration, Supervision, Funding acquisition. **GH**: Methodology, Data curation, Formal analysis, Writing – Review & Editing, Supervision, Project administration. All authors contributed revisions to the text, approved the final version and agree to be accountable for the work.

Competing interest

Authors declare no competing interests.

Funding

Funding for the study on which these data are based is provided by the Alexander von Humboldt Foundation through an award to Professor Bärnighausen. GH was supported by a fellowship from the Royal Society and the Wellcome Trust [Grant number 210479/Z/18/Z]. This research was funded in whole, or in part, by the Wellcome Trust [Grant number 210479/Z/18/Z]. For the purpose of open access, the authors have applied a CC BY public copyright licence to any Author Accepted Manuscript version arising from this submission.

Data sharing statement

The data that support the findings of this study are available from the corresponding author, TB, upon reasonable request and after signing a data use agreement. The data are not publicly available due to the risk of participant re-identification given the large amount of information contained for each person.

Ethical considerations

The Nouna ARISE study was approved by CRSN's Institutional Ethics Committee (2017/08 and 2018/017) and Heidelberg Medical Faculty's Ethics Commission (S-607/2018). Secondary

For peer review only - http://bmjopen.bmj.com/site/about/guidelines.xhtml

data analysis was approved by University College London's Research Ethics Committee (15231/005). Prior to the study village elder provided oral assent. Each participants provided written informed consent; for respondents aged under 18, parents or guardians provided written consent alongside the minor's written assent. In cases of illiteracy, a literate witness assisted.

For peer review only

For peer review only - http://bmjopen.bmj.com/site/about/guidelines.xhtml

References

1. World Health organization, *Global Status report on Viloence Prevention*. 2014.

2. Krug, E.G., et al., *The world report on violence and health*, in *Lancet*. 2002. p. 1083-8.

3. Golshiri, P., et al., *Youth Violence and Related Risk Factors: A Cross-sectional Study in 2800 Adolescents.* Adv Biomed Res, 2018. **7**: p. 138.

4. World Health Organization, *Preventing youth violence: an overview of the evidence*. 2015.

5. Bellis, M.A., et al., *Adverse childhood experiences and associations with health-harming behaviours in young adults: surveys in eight eastern European countries.* Bulletin of the World Health Organization, 2014. **92**: p. 641-655.

6. World Health Organization. *Youth Violence*. 2019; Available from: https://www.who.int/health-topics/violence-against-children#tab=tab_3.

7. Ward, C.L., A. Dawes, and R. Matzopoulos, *Youth violence in South Africa: Setting the scene*, in *Youth violence: Sources and Solutions in South Africa*, C.L. Ward, A. Van der Merwe, and A. Dawes, Editors. 2013, University of Cape Town Press: Cape Town. p. 1-20.

8. Lamb, G., *Social cohesion and violence in South Africa: constructing a puzzle with missing pieces.* Crime, Law and Social Change, 2019. **72**(4): p. 365-385.

9. Rumble, L., et al., *Childhood sexual violence in Indonesia: a systematic review.* Trauma, Violence and Abuse, 2020. **21**(2): p. 284-299.

10. Iman'ishimwe Mukamana, J., P. Machakanja, and N.K. Adjei, *Trends in prevalence and correlates of intimate partner violence against women in Zimbabwe, 2005-2015.* BMC Int Health Hum Rights, 2020. **20**(1): p. 2.

For peer review only - http://bmjopen.bmj.com/site/about/guidelines.xhtml

11. Malta, D.C., et al., *Factors associated with family violence against adolescents based on the results of the National School Health Survey (PeNSE).* Cien Saude Colet, 2019. **24**(4): p. 1287-1298.

12. World Health Organization, *World Bank Group Gender Strategy (FY16-23) : Gender Equality, Poverty Reduction and Inclusive Growth*. 2015: World Bank, Washington, DC. © World Bank. .

13. Shamu, S., et al., *A systematic review of African studies on intimate partner violence against pregnant women: prevalence and risk factors.* PLoS One, 2011. **6**(3): p. e17591.

14. Girmay, A., et al., *Intimate partner physical violence and associated factors in reproductive age married women in Aksum Town, Tigray, Ethiopia 2018, and community based study.* BMC Res Notes, 2019. **12**(1): p. 627.

15. Breiding MJ, S.S., Basile KC, Walters ML, Chen J, & Merrick MT, *Prevalence and characteristics of sexual violence, stalking, and intimate partner violence victimization—National Intimate Partner and Sexual Violence Survey, United States, 2011.* Morbidity and Mortality Weekly Report, 2014. **63**(8): p. 1-18.

16. Baigorria, J., et al., *[Prevalence and associated factors with sexual violence against women: systematic review].* Rev Salud Publica (Bogota), 2017. **19**(6): p. 818-826.

17. Belay, S., et al., *Intimate partner violence and maternal depression during pregnancy: A community-based cross-sectional study in Ethiopia.* PLoS One, 2019. **14**(7): p. e0220003.

18. Semahegn, A. and B. Mengistie, *Domestic violence against women and associated factors in Ethiopia; systematic review.* Reprod Health, 2015. **12**: p. 78.

19. Puri, M., et al., *The prevalence and determinants of sexual violence against young married women by husbands in rural Nepal.* BMC research notes, 2012. **5**(1): p. 291.

20. Heise, L.L., *Violence against women: an integrated, ecological framework.* Violence Against Women, 1998. **4**(3): p. 262-90.

For peer review only - http://bmjopen.bmj.com/site/about/guidelines.xhtml

21. Ttofi, M.M., et al., *Do the victims of school bullies tend to become depressed later in life? A systematic review and meta-analysis of longitudinal studies.* Journal of Aggression, Conflict and Peace Research, 2011. **3**(2): p. 63-73.

22. Valido, A., et al., *Intra-familial Violence and Peer Aggression Among Early Adolescents: Moderating Role of School Sense of Belonging.* Journal of Family Violence, 2020. **36**: p. 87-98.

23. de Ribera, O.S., et al., *Correlates of youth violence in low-and middle-income countries: A meta-analysis.* Aggression and Violent Behavior, 2019. **49**: p. 101306.

24. Mason-Jones, A.J., et al., *Intimate partner violence in early adolescence: The role of gender, socioeconomic factors and the school.* South African Medical Journal, 2016. **106**(5): p. 502-509.

25. Masuda, K. and C. Yamauchi, *How Does Female Education Reduce Adolescent Pregnancy and Improve Child Health?: Evidence from Uganda's Universal Primary Education for Fully Treated Cohorts.* The Journal of Development Studies, 2020. **56**(1): p. 63-86.

26. Obradovic-Tomasevic, B., et al., *Prevalence and Predictors of Violence Victimization and Violent Behavior among Youths: A Population-Based Study in Serbia.* Int J Environ Res Public Health, 2019. **16**(17): p. 3203.

27. Behrman, J.A., A. Peterman, and T. Palermo, *Does Keeping Adolescent Girls in School Protect Against Sexual Violence? Quasi-Experimental Evidence From East and Southern Africa.* Journal of Adolescent Health, 2017. **60**(2): p. 184-190.

28. Ackerson, L.K., et al., *Effects of individual and proximate educational context on intimate partner violence: a population-based study of women in India.* American journal of public health, 2008. **98**(3): p. 507-514.

29. Adu Boahen, E. and C. Yamauchi, *The effect of female education on adolescent fertility and early marriage: Evidence from free compulsory universal basic education in Ghana.* Journal of African Economies, 2018. **27**(2): p. 227-248.

For peer review only

For peer review only - http://bmjopen.bmj.com/site/about/guidelines.xhtml

30. World Health Organization. *WHO Country Cooperation Strategy at a glance: Burkina Faso*. 2016 [cited 2019 19/03/2019]; Available from: https://apps.who.int/iris/handle/10665/136973.

31. de Ribera, O.S., et al., *Correlates of youth violence in low-and middle-income countries: A meta-analysis.* 2019. **49**: p. 101306.

32. van der Merwe, A. and A. Dawes, *Youth violence: A review of risk factors, causal pathways and effective intervention.* Journal of Child and Adolescent Mental Health, 2007. **19**(2): p. 95-113.

33. World Health Organization. *World Health Statistics 2015*. 2015 [cited 2018 10/06/2018]; Available from: https://www.who.int/gho/publications/world_health_statistics/2015/en/.

34. World Health Organization, *Country Cooperation Strategy at a glance: Burkina Faso*. 2016.

35. Burkina Faso Mininstere de L'Economie des Finances et du Development Secretariat General, *Tableau de Bord Social du Burkina Faso*. 2017, INSTITUT NATIONAL DE LA STATISTIQUE ET DE LA DEMOGRAPHIE

36. Wirtz, A.L., et al., *Sexual violence, condom negotiation, and condom use in the context of sex work: results from two West African countries.* J Acquir Immune Defic Syndr, 2015. **68 Suppl 2**: p. S171-9.

37. Ouédraogo, S.Y.Y.A., E.J. Sisawo, and S.-L. Huang, *Sexual abuse and risky sexual behaviors among young female hawkers in Burkina Faso: a mixed method study.* BMC international health and human rights, 2017. **17**(1): p. 1.

38. Ismayilova, L., et al., *Maltreatment and mental health outcomes among ultra-poor children in Burkina Faso: A latent class analysis.* Plos One, 2016. **11**(10): p. e0164790.

39. Ismayilova, L. and L. Karimli, *Harsh parenting and violence against children: a trial with ultrapoor families in Francophone West Africa.* Journal of Clinical, Child and Adult psychology, 2020. **49**(1): p. 18-35.

For peer review only - http://bmjopen.bmj.com/site/about/guidelines.xhtml

40. Devries, K., et al., *Violence against children in Latin America and the Caribbean: What do available data reveal about prevalence and perpetrators?* Revista panamericana de salud publica, 2019. **43**: p. e66.

41. Farrington, D.P. and M.M. Ttofi, *Advancing Knowledge about Youth Violence: Child Maltreatment, Bullying, Dating Violence, and Intimate Partner Violence.* Journal of Family Violence, 2020. **36**(1): p. 109-115.

42. Sié, A., et al., *The Health and Demographic Surveillance System (HDSS) in Nouna, Burkina Faso, 1993-2007.* Glob Health Action, 2010. **3**(1).

43. Darling, A.M., et al., *Design and field methods of the ARISE Network Adolescent Health Study.* Journal of Tropical Medicine and International Health, 2020. **25**(1): p. 5-14.

44. Greis, A., et al., *Attitudes towards female genital cutting among adolescents in rural Burkina Faso: A multilevel analysis Female genital cutting in Burkina Faso.* Journal of Tropical Medicine and International Health, 2019. **25**(1): p. 119-131.

45. Bushman, B.J., et al., *Youth violence: What we know and what we need to know.* American Psychologist, 2016. **71**(1): p. 17.

46. Tripathi, V., *Youth violence and social media.* Journal of Social Sciences, 2017. **52**(1-3): p. 1-7.

47. Khandker, S.R., G.B. Koolwal, and H.A. Samad, *Handbook on impact evaluation: quantitative methods and practices*. 2010: World Bank Publications.

48. Gertler, P.J., et al., *Impact Evaluation in Practice*. Second ed. 2016, Washington D.C: International Bank for Reconstruction and Development/The World Bank.

49. Abramsky, T., et al., *What factors are associated with recent intimate partner violence? Findings from the WHO multi-country study on women's health and domestic violence.* BMC public health, 2011. **11**(1): p. 1-17.

For peer review only - http://bmjopen.bmj.com/site/about/guidelines.xhtml

50. Black, E., et al., *Prevalence and correlates of intimate partner violence against women in conflict affected northern Uganda: a cross-sectional study.* Confl Health, 2019. **13**: p. 35.

51. Fahlgren, M.K., et al., *Age and gender effects in recent violence perpetration.* Journal of interpersonal violence, 2020. **35**(17-18): p. 3513-3529.

52. Tashjian, S.M., J.L. Mullins, and A. Galván, *Bedtime autonomy and cellphone use influence sleep duration in adolescents.* Journal of Adolescent Health, 2019. **64**(1): p. 124-130.

53. Estrada-Martínez, L.M., et al., *Families, Neighborhood Socio-Demographic Factors, and Violent Behaviors among Latino, White, and Black Adolescents.* Youth & Society, 2011. **45**(2): p. 221-242.

54. Kellermann, A.L., et al., *Preventing youth violence: what works?* Annual review of public health, 1998. **19**(1): p. 271-292.

55. Botvin, G.J., K.W. Griffin, and T.D. Nichols, *Preventing youth violence and delinquency through a universal school-based prevention approach.* Prevention science, 2006. **7**(4): p. 403-408.

56. Lebrun-Harris, L.A., et al., *Bullying victimization and perpetration among US children and adolescents: 2016 National Survey of Children's Health.* Journal of Child and Family Studies, 2019. **28**(9): p. 2543-2557.

57. Aboagye, R.G., et al., *A multi-country analysis of the prevalence and factors associated with bullying victimisation among in-school adolescents in sub-Saharan Africa: evidence from the global school-based health survey.* BMC Psychiatry, 2021. **21**(1): p. 325.

58. Ashrafi, A., et al., *Bullying victimization among preadolescents in a community-based sample in Canada: a latent class analysis.* BMC research notes, 2020. **13**(1): p. 138.

59. Hokoda, A., H.-H.A. Lu, and M. Angeles, *School bullying in Taiwanese adolescents.* Journal of Emotional Abuse, 2006. **6**(4): p. 69-90.

For peer review only - http://bmjopen.bmj.com/site/about/guidelines.xhtml

60. Salmon, S., et al., *Bullying victimization experiences among middle and high school adolescents: Traditional bullying, discriminatory harassment, and cybervictimization.* Journal of Adolescence, 2018. **63**: p. 29-40.

61. Amegbor, P.M. and L. Pascoe, *Variations in Emotional, Sexual, and Physical Intimate Partner Violence Among Women in Uganda: A Multilevel Analysis.* J Interpers Violence, 2019. **36**(15-16): p. NP7868-NP7898.

62. Cofie, N., *A multilevel analysis of contextual risk factors for intimate partner violence in Ghana.* international Review of Victimology, 2020. **26**(1): p. 50-78.

63. Imai, K. and I.S. Kim, *When should we use unit fixed effects regression models for causal inference with longitudinal data?* American Journal of Political Science, 2019. **63**(2): p. 467-490.

For peer review only - http://bmjopen.bmj.com/site/about/guidelines.xhtml

Figure 1: Flow chart of sample

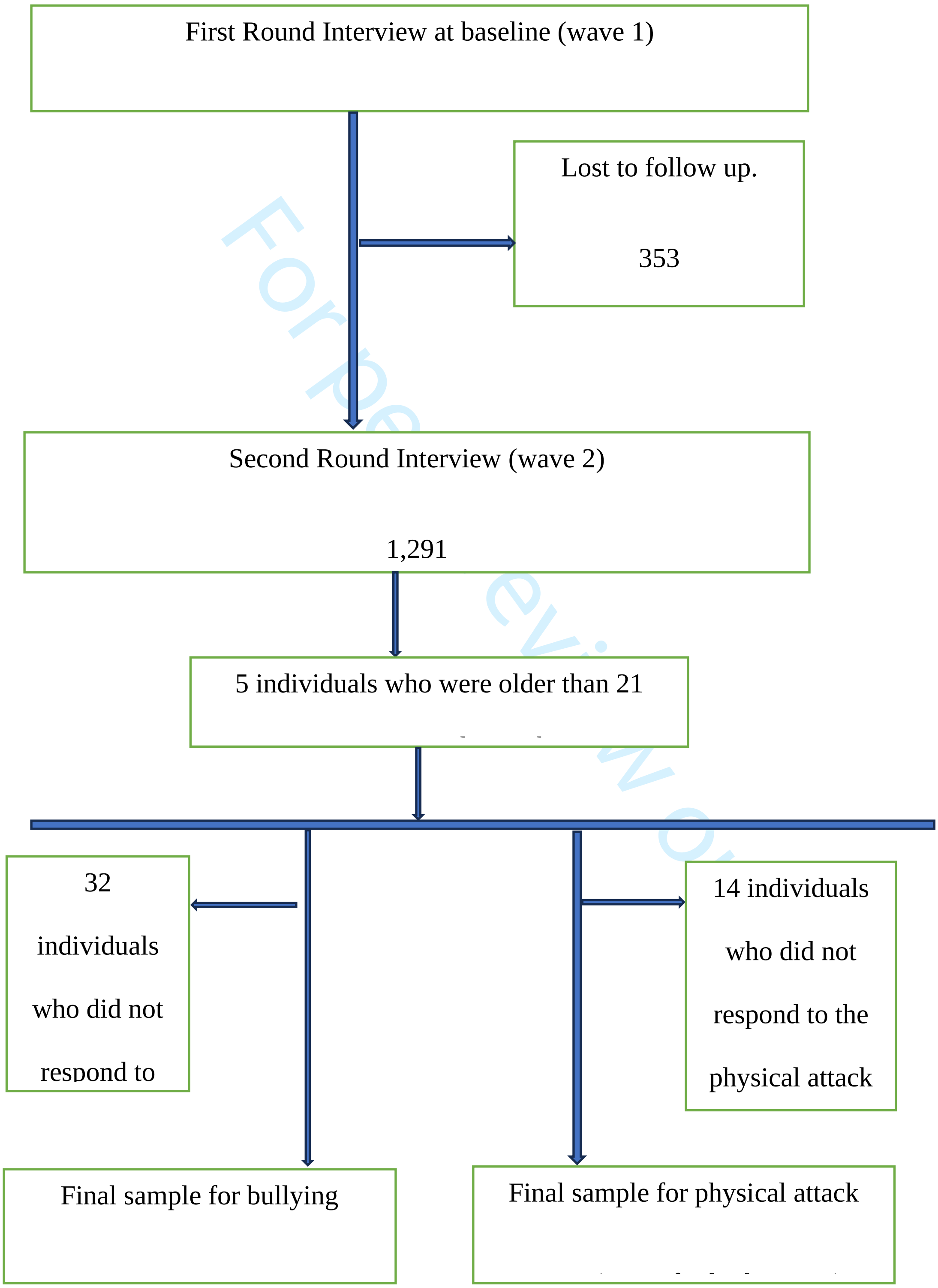

Figure 2. Exposure distribution among respondents

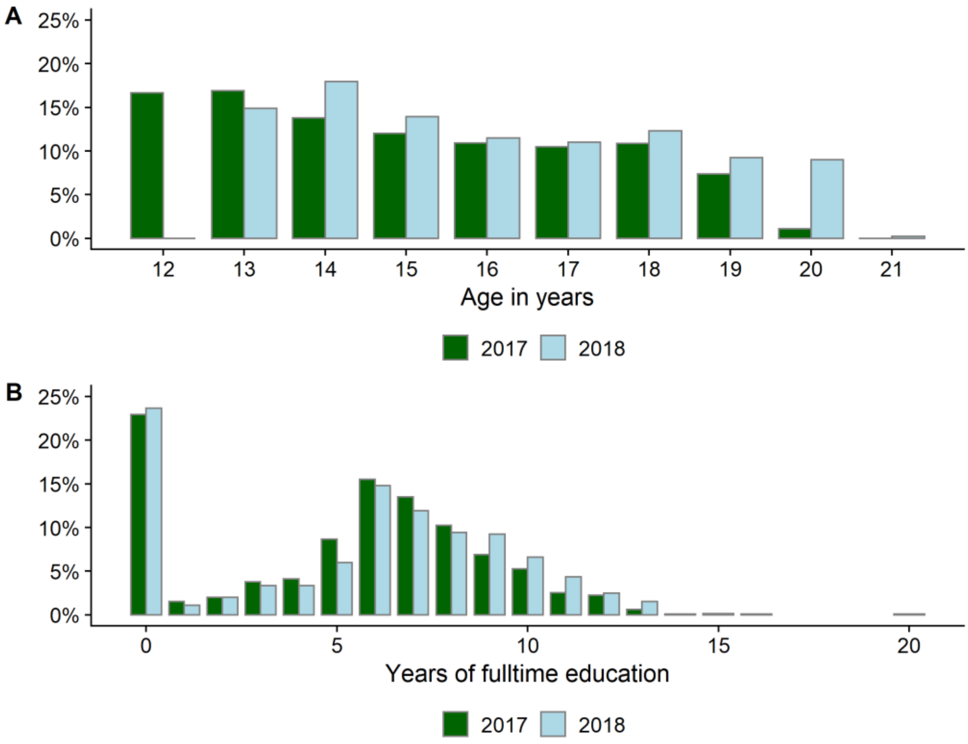

For peer review only - http://bmjopen.bmj.com/site/about/guidelines.xhtml

SUPPLEMENTARY MATERIAL

Title: The effects of education and age on experience of youth violence in a very low-income setting: a fixed-effects analysis from rural Burkina Faso

Supplementary Table 1a: Variables and their measurements

	Measurement
Outcome	
Violence experience (VE1)	Ever experienced bullying, physical attack or unwelcome sexual advances in the last 6 months
Bullying	Yes / No
Physical attack	Yes / No
Sexual violence	Yes / No
Violence perpetration (VP1)	Ever perpetrated bullying or physical attack in the last 6 months
Bullying	Yes / No
Physical attack	Yes / No
Individual level factors	
Age	Age in complete years
Education	
Level of schooling	Primary, post-primary or secondary level
School attainment	Grade/class/standard
Years of schooling	Years of education completed in years
Highest level of schooling	Primary, post-primary, secondary, technical/vocational or university
Marital status	Single, engaged, married (monogamous), married (polygamous), civil union, separated/divorced or widowed
Experienced first sex	Had first sex in the last 6 months (Yes / No)
Multiple sex partners	Had sex with more than one person in your life time (Yes / No)
Number of sexual partners	Number of life time sexual partners
Employment	Done any work in the last 12 months to earn money or obtain food (Yes/ No)
Alcohol use	Ever drunk alcohol on your own (Yes / No)
Substance use	Ever used drugs (Yes / No)
Household level factors	
Household asset	Asset index for household
Household size	Number of persons living in household
Aged 0 – 14 years	
Aged 15 – 29 years	
Aged 30 – 44 years	
Aged 45 – 59 years	
Aged 60 and above	
Moving between household	Sometimes live in another household other than primary household (Yes / No)
Living with adult figure	Yes / No
Adult figure	Father / Mother
Age of adult figure	Age in complete years

For peer review only - http://bmjopen.bmj.com/site/about/guidelines.xhtml

Less than 30 years	
30 – 45 years	
46 – 60 years	
Above 60 years	
No adult figure	
Educational level adult figure Male Female	Primary, post-primary, secondary, technical/vocational or university
Occupation of adult figure Male Female	Farmer, merchant, teacher, other government worker, other (specified)
Frequency of parental /guardian support	In the last 30 days how often did parent/ guardian……
With important life decisions	Helped you make important decisions (never, rarely, sometimes, most of the times or always)
With understanding problems	Understand your problems and worries (never, rarely, sometimes, most of the times or always)
Talking about what happens	Take time to talk with you about things that happen to you (never, rarely, sometimes, most of the times or always)
Paying attention to opinion	Pay attention to your opinion (never, rarely, sometimes, most of the times or always)
Media use	
Access to media	
Radio	Yes / No
TV	Yes / No
Magazines	Yes / No
Use of media	
Radio	Average number of hours per day spent listening to radio
TV_1	Frequency of watching TV [never, rarely (some hours per month), often (some hours per week) or very frequently (several hours per day)]
TV_2	Average number of hour per day spent watching TV
Magazines	How often do you read magazines [never, rarely (some hours per month), often (some hours per week) or very frequently (several hours per day)]
Community level factor	
Access to education	Community share of school enrolment

For peer review only - http://bmjopen.bmj.com/site/about/guidelines.xhtml

Supplementary Table 2: Parental support (n=1291)

Variable	2017		2018	
	Frequency	Percent	Frequency	Percent
Parents understand worries				
Never	299	23.27	68	5.29
Rarely	201	15.64	193	15.01
Sometimes	271	21.09	391	30.40
Most of the time	159	12.37	295	22.94
Always	351	27.32	339	26.36
Missing	4	0.31		
Parents help make decisions				
Never	212	16.50	27	2.10
Rarely	257	20.00	193	15.01
Sometimes	311	24.20	350	27.22
Most of the time	159	12.37	331	25.74
Always	337	26.23	384	29.86
Missing	9	0.70	1	0.08
Parents talk about things that happen				
Never	299	23.27	83	6.45
Rarely	253	19.69	268	20.84
Sometimes	324	25.21	333	25.89
Most of the time	161	12.53	340	26.44
Always	243	18.91	262	20.37
Missing	5	0.39		
Parents pay attention to what you say				
Never	317	24.67	94	7.31
Rarely	296	23.04	225	17.50
Sometimes	381	29.65	416	32.35
Most of the time	133	10.35	236	18.35
Always	153	11.91	314	24.42
Missing	5	0.39	1	0.08

We built a summative score of parental support by adding the four variables, with a minimum score of 0 and a maximum score of 16.

For peer review only

Supplementary Table 3: Difference between those who dropped out in wave 2 and remaining sample.

	participation_w2 = 0	participation_w2 = 1	Test	Statistic	p-value
	N=353	N=1,291			
Number of physical attacks, last 12m	0 (0-0)	0 (0-0)	Wilcoxon rank-sum	Z= -0.74	0.46
Days bullied, last 30d	0 (0-0)	0 (0-0)	Wilcoxon rank-sum	Z= -1.18	0.24
Gender			Chi-square	Chi2(1)= 4.55	0.033
Female	167 (47.3%)	529 (41.0%)			
Male	186 (52.7%)	762 (59.0%)			
Age (years)	15 (14-18)	15 (13-17)	Wilcoxon rank-sum	Z= 3.64	<0.001
Marital status cat			Chi-square	Chi2(1)= 2.08	0.15
other	*41 (11.6%)*	*117 (9.1%)*			
single	*312 (88.4%)*	*1,174 (90.9%)*			
Ever worked	222 (62.9%)	786 (60.9%)	Chi-square	Chi2(1)= 0.47	0.49
Mother is alive	342 (96.9%)	1,261 (97.7%)	Chi-square	Chi2(1)= 0.72	0.40
Father is alive	316 (89.5%)	1,186 (91.9%)	Chi-square	Chi2(1)= 1.94	0.16
Lives with Mother	262 (74.2%)	1,005 (77.8%)	Chi-square	Chi2(1)= 2.06	0.15
Lives with father	245 (69.4%)	965 (74.7%)	Chi-square	Chi2(1)= 4.07	0.044
Lives alone	23 (6.5%)	56 (4.3%)	Chi-square	Chi2(1)= 2.87	0.090
Has own bedroom	58 (16.4%)	228 (17.7%)	Chi-square	Chi2(1)= 0.29	0.59
Wealth quintile			Chi-square	Chi2(4)= 1.12	0.89
1	*76 (21.5%)*	*255 (19.8%)*			
2	*74 (21.0%)*	*257 (19.9%)*			
3	*73 (20.7%)*	*282 (21.8%)*			
4	*64 (18.1%)*	*235 (18.2%)*			
5	*66 (18.7%)*	*262 (20.3%)*			
Currently in school	119 (33.7%)	703 (54.5%)	Chi-square	Chi2(1)= 47.71	<0.001
Current school level			Chi-square	Chi2(4)= 53.56	<0.001
None	*234 (67.2%)*	*589 (46.2%)*			
Primary (1-6)	*40 (11.5%)*	*268 (21.0%)*			
Post Primary (7-10)	*66 (19.0%)*	*386 (30.3%)*			
Secondary (1-3)	*7 (2.0%)*	*33 (2.6%)*			
Technical/Vocational	*1 (0.3%)*	*0 (0.0%)*			
Has access to TV	295 (83.6%)	1,035 (80.2%)	Chi-square	Chi2(1)= 2.07	0.15
Frequency of watching TV			Chi-square	Chi2(3)= 2.69	0.44
Never	*59 (16.8%)*	*263 (20.4%)*			
Rarely (some hours per month)	*88 (25.0%)*	*290 (22.5%)*			

For peer review only - http://bmjopen.bmj.com/site/about/guidelines.xhtml

Often (several hours per week)	*151 (42.9%)*	*540 (41.9%)*			
Very often (several hours per day)	*54 (15.3%)*	*197 (15.3%)*			
Frequency of reading magazines			Chi-square	Chi2(3)= 6.12	0.11
Never	*334 (94.6%)*	*1,177 (91.2%)*			
Rarely (some hours per month)	*8 (2.3%)*	*30 (2.3%)*			
Often (several hours per week)	*5 (1.4%)*	*30 (2.3%)*			
Very often (several hours per day)	*6 (1.7%)*	*54 (4.2%)*			
Ever had intercourse?	*55 (16.8%)*	*202 (17.0%)*	*Chi-square*	*Chi2(1)= 0.01*	*0.93*
Number of sex partners, lifetime			Chi-square	Chi2(11)= 6.01	0.87
0	*298 (86.4%)*	*1,087 (86.3%)*			
1	*29 (8.4%)*	*121 (9.6%)*			
2	*9 (2.6%)*	*25 (2.0%)*			
3	*5 (1.4%)*	*14 (1.1%)*			
4	*1 (0.3%)*	*3 (0.2%)*			
5	*0 (0.0%)*	*4 (0.3%)*			
6	*1 (0.3%)*	*1 (0.1%)*			
7	*0 (0.0%)*	*1 (0.1%)*			
8	*1 (0.3%)*	*1 (0.1%)*			
9	*0 (0.0%)*	*1 (0.1%)*			
10	*1 (0.3%)*	*1 (0.1%)*			
17	*0 (0.0%)*	*1 (0.1%)*			
Scores for component 1	-.2998273 (-1.694384-.8893526)	-.6083845 (-1.707631-.9265742)	Wilcoxon rank-sum	Z= 0.57	0.57
Parental_support	2 (1-2.75)	1.75 (1-2.75)	Wilcoxon rank-sum	Z= 0.71	0.48

Data are presented as median (IQR) for continuous measures, and n (%) for categorical measures.

For peer review only

For peer review only - http://bmjopen.bmj.com/site/about/guidelines.xhtml

Supplementary Table 4: Full regression models predicting violence experiences

Variables	Bullying		Physical Attacks	
	Model 2 Adjusted	Model 3 Adjusted & interaction	Model 2 Adjusted	Model 3 Adjusted & interaction
Interview 2 vs interview 1	-0.42[-0.75,-0.08]	-0.44 [-0.78,-0.11]	-0.10[-0.27,0.07]	-0.09[-0.26,0.08]
Marital status (vs other)				
Single	-0.20[-1.03,0.63]	-0.24[-1.07, 0.59]	0.03[-0.40,0.46]	0.05[-0.38,0.48]
Ever worked	0.01[-0.27,0.28]	0.01[-0.27,0.28]	0.05[-0.09,0.20]	0.05[-0.09,0.19]
Number of people in the household	-0.04 [-0.07,-0.02]	-0.04 [-0.07,-0.02]	-0.01[-0.02,0.00]	-0.01[-0.02,0.00]
Mother is alive	0.74[-1.34, 2.82]	0.77[-1.31,2.85]	-0.30[-1.38,0.77]	-0.32[-1.40,0.76]
Father is alive	1.12[-2.41,0.17]	-1.11[-2.41, 0.18]	-0.35[-0.99,0.28]	-0.34[-0.98,0.29]
Lives with Mother	-0.12[-0.74,0.51]	-0.15[-0.77,0.48]	0.06[-0.26,0.39]	0.08[-0.25,0.40]
Lives with father	0.46[-0.18,1.10]	0.45[-0.18,1.09]	-0.13[-0.46,0.20]	-0.12[-0.45,0.21]
Lives alone	-0.17[-1.03,0.69]	-0.14[-1.00,0.72]	-0.38[-0.83,0.07]	-0.40[-0.84,0.05]
Parental support	-0.18 [-0.31,-0.05]	-0.18 [-0.31,-0.05]	-0.04[-0.11,0.03]	-0.04[-0.11,0.03]
Household wealth quintile				
Lowest (base)				
2	0.08[-0.34,0.50-]	0.06[-0.36,0.49]	-0.05[-0.27,0.17]	-0.04[-0.26,0.17]
3	-0.01[-0.44,0.42]	-0.02[-0.44,0.41]	-0.06[-0.28,0.16]	-0.06[-0.28,0.16]
4	-0.04[-0.52,0.44]	-0.07[-0.55,0.41]	-0.08[-0.32,0.17]	-0.06[-0.31,0.18]
Highest	0.43[-0.15,1.00]	0.40[-0.18,0.98]	0.12[-0.18,0.41]	0.13[-0.17,0.42]
Has own bedroom	-0.08[0.47,0.32]	-0.08[-0.47,0.32]	-0.01[-0.21,0.20]	-0.01[-0.21,0.20]
Radio hours/day	0.13[-0.01,0.27]	0.13[-0.00,0.27]	-0.02[-0.09,0.05]	-0.02[-0.09,0.05]
Frequency of watching television				
Never (base)				
Rarely (some hours per month)	-0.34[0.72,0.03]	-0.29[-0.67,0.09]	0.06[-0.13,0.26]	0.03[-0.16,0.23]
Often (several hours per week)	-0.11[-0.46,0.24]	-0.07[-0.42,0.29]	-0.04[-0.22,0.14]	-0.06-0.24,0.12]
Very often (several hours per day)	0.63 [0.14,1.11]	0.63 [0.15,1.12]	-0.19[00.44,0.06]	-0.19[-0.44,0.06]
Frequency of reading magazines				

For peer review only - http://bmjopen.bmj.com/site/about/guidelines.xhtml

Never (base)				
Rarely (some hours per month)	1.01 [0.46,1.57]	0.99 [0.43,1.54]	-0.18[-0.47,0.11]	-0.17[-0.46,0.12]
Often (several hours per week)	1.12 [0.38,1.85]	1.08 [0.35,1.81]	-0.22[-0.61,0.16]	-0.21[-0.59,0.17]
Very often (several hours per day)	-0.17[-1.06,0.73]	-0.07[-0.97,0.83]	0.90 [0.44,1.36]	0.85 [0.38,1.31]
Household size missing	-1.77[-3.73, 0.20]	-1.68[-3.64, 0.29]	-0.46[-1.39,0.46]	-0.50[-1.43,0.43]
Freq. of reading magazine missing	6.99 [4.23, 9.75]	6.93 [4.16,9.69]	0.46[-0.97,1.89]	0.49[-0.94,1.92]

All models present coefficients and 95% confidence intervals. All models contain fixed effects for each respondent. Age centred at 15.

For peer review only

For peer review only - http://bmjopen.bmj.com/site/about/guidelines.xhtml

STROBE Statement—Checklist of items that should be included in reports of ***cross-sectional studies***

	Item No	Recommendation	Reported on page no.
**Title and abstract**	1	(*a*) Indicate the study's design with a commonly used term in the title or the abstract	6
		(*b*) Provide in the abstract an informative and balanced summary of what was done and what was found	1 – 2
**Introduction**			
Background/rationale	2	Explain the scientific background and rationale for the investigation being reported	2 – 5
Objectives	3	State specific objectives, including any prespecified hypotheses	5 – 6
**Methods**			
Study design	4	Present key elements of study design early in the paper	6
Setting	5	Describe the setting, locations, and relevant dates, including periods of recruitment, exposure, follow-up, and data collection	6 – 7
Participants	6	(*a*) Give the eligibility criteria, and the sources and methods of selection of participants	6
Variables	7	Clearly define all outcomes, exposures, predictors, potential confounders, and effect modifiers. Give diagnostic criteria, if applicable	7
Data sources/ measurement	8*	For each variable of interest, give sources of data and details of methods of assessment (measurement). Describe comparability of assessment methods if there is more than one group	6 -7
Bias	9	Describe any efforts to address potential sources of bias	8 - 9
Study size	10	Explain how the study size was arrived at	6
Quantitative variables	11	Explain how quantitative variables were handled in the analyses. If applicable, describe which groupings were chosen and why	8 – 9
Statistical methods	12	(*a*) Describe all statistical methods, including those used to control for confounding	8 – 9
		(*b*) Describe any methods used to examine subgroups and interactions	9
		(*c*) Explain how missing data were addressed	8 – 9
		(*d*) If applicable, describe analytical methods taking account of sampling strategy	N/A
		€ Describe any sensitivity analyses	N/A
**Results**			
Participants	13*	(a) Report numbers of individuals at each stage of study—eg numbers potentially eligible, examined for eligibility, confirmed eligible, included in the study, completing follow-up, and analysed	See page 4 of supplementary material
		(b) Give reasons for non-participation at each stage	
		(c) Consider use of a flow diagram	
Descriptive data	14*	(a) Give characteristics of study participants (eg demographic,	10

For peer review only

		clinical, social) and information on exposures and potential confounders	
		(b) Indicate number of participants with missing data for each variable of interest	See page 4 of supplementary material
Outcome data	15*	Report numbers of outcome events or summary measures	10
Main results	16	(*a*) Give unadjusted estimates and, if applicable, confounder-adjusted estimates and their precision (eg, 95% confidence interval). Make clear which confounders were adjusted for and why they were included	12
		(*b*) Report category boundaries when continuous variables were categorized	N/A
		© If relevant, consider translating estimates of relative risk into absolute risk for a meaningful time period	N/A
Other analyses	17	Report other analyses done—eg analyses of subgroups and interactions, and sensitivity analyses	12 and Table 3
**Discussion**			
Key results	18	Summarise key results with reference to study objectives	17
Limitations	19	Discuss limitations of the study, taking into account sources of potential bias or imprecision. Discuss both direction and magnitude of any potential bias	16
Interpretation	20	Give a cautious overall interpretation of results considering objectives, limitations, multiplicity of analyses, results from similar studies, and other relevant evidence	16 - 17
Generalisability	21	Discuss the generalisability (external validity) of the study results	17
**Other information**			
Funding	22	Give the source of funding and the role of the funders for the present study and, if applicable, for the original study on which the present article is based	17

*Give information separately for exposed and unexposed groups.

Note: An Explanation and Elaboration article discusses each checklist item and gives methodological background and published examples of transparent reporting. The STROBE checklist is best used in conjunction with this article (freely available on the Web sites of PLoS Medicine at http://www.plosmedicine.org/, Annals of Internal Medicine at http://www.annals.org/, and Epidemiology at http://www.epidem.com/). Information on the STROBE Initiative is available at www.strobe-statement.org.

For peer review only

BMJ Open

The effects of education and age on the experience of youth violence in a very low-resource setting: a fixed-effects analysis in rural Burkina Faso.

Journal:	*BMJ Open*
Manuscript ID	bmjopen-2022-071104.R2
Article Type:	Original research
Date Submitted by the Author:	31-Aug-2023
Complete List of Authors:	Kuunibe, Naasegnibe; Simon Diedong Dombo University of Business and Integrated Development Studies,<br>Bountogo, Mamadou; Centre de Recherche en Sante de Nouna, Nouna; Université de Ouagadougou, Ouaga<br>Ouermi, Lucienne; Centre de Recherche en Sante de Nouna<br>Sié, Ali; Centre de Recherche en Sante de Nouna Nouna, Boucle du Mouhoun, BF<br>Bärnighausen, Till ; Heidelberg University, Heidelberg Institute of Global Health<br>Harling, Guy; University College London, Institute for Global Health; Harvard University T H Chan School of Public Health, Department of Epidemiology
**Primary Subject Heading**:	Public health
Secondary Subject Heading:	Public health
Keywords:	HEALTH ECONOMICS, PUBLIC HEALTH, STATISTICS & RESEARCH METHODS, Adolescent

SCHOLARONE™
Manuscripts

I, the Submitting Author has the right to grant and does grant on behalf of all authors of the Work (as defined in the below author licence), an exclusive licence and/or a non-exclusive licence for contributions from authors who are: i) UK Crown employees; ii) where BMJ has agreed a CC-BY licence shall apply, and/or iii) in accordance with the terms applicable for US Federal Government officers or employees acting as part of their official duties; on a worldwide, perpetual, irrevocable, royalty-free basis to BMJ Publishing Group Ltd ("BMJ") its licensees and where the relevant Journal is co-owned by BMJ to the co-owners of the Journal, to publish the Work in this journal and any other BMJ products and to exploit all rights, as set out in our licence.

The Submitting Author accepts and understands that any supply made under these terms is made by BMJ to the Submitting Author unless you are acting as an employee on behalf of your employer or a postgraduate student of an affiliated institution which is paying any applicable article publishing charge ("APC") for Open Access articles. Where the Submitting Author wishes to make the Work available on an Open Access basis (and intends to pay the relevant APC), the terms of reuse of such Open Access shall be governed by a Creative Commons licence – details of these licences and which Creative Commons licence will apply to this Work are set out in our licence referred to above.

Other than as permitted in any relevant BMJ Author's Self Archiving Policies, I confirm this Work has not been accepted for publication elsewhere, is not being considered for publication elsewhere and does not duplicate material already published. I confirm all authors consent to publication of this Work and authorise the granting of this licence.

For peer review only

The effects of education and age on the experience of youth violence in a very low-resource setting: a fixed-effects analysis in rural Burkina Faso.

Naasegnibe Kuunibe[1,2*], Mamadou Bountogo[3], Lucienne Ouermi[3], Ali Sié[3], Till Bärnighausen[2,4,5,], Guy Harling[4,6,7,8,9]

* Corresponding Author:

P O Box WA64, Wa, Upper West Region Ghana

nkuunibe@ubids.edu.gh / +233(0)209006165.

1. Department of Economics, Faculty of Social Science and Arts, SD-Dombo University of Business and Integrated Development Studies, Wa, Ghana
2. Heidelberg Institute for Global Health, Heidelberg University Hospital, Heidelberg, Germany
3. Centre de Recherche en Santé de Nouna, Nouna, Burkina Faso
4. Africa Health Research Institute, KwaZulu-Natal, South Africa
5. Department of Global Health and Population, Harvard T.H. Chan School of Public Health, Boston, MA, USA
6. Institute for Global Health, University College London, London, United Kingdom
7. MRC/Wits Rural Public Health & Health Transitions Research Unit (Agincourt), University of the Witwatersrand, South Africa
8. Department of Epidemiology & Harvard Center for Population and Development Studies, Harvard T.H. Chan School of Public Health, Boston, MA, USA
9. School of Nursing & Public Health, College of Health Sciences, University of KwaZulu-Natal, KwaZulu-Natal, South Africa

Keywords: Adolescence, physical violence, bullying, panel fixed-effects, Burkina Faso.

Word count: 3679

ABSTRACT

Objective: The study aimed to investigate the effects of education and age on the experience of youth violence in low- and middle-income country settings.

For peer review only - http://bmjopen.bmj.com/site/about/guidelines.xhtml

Design: Using a standardised questionnaire, our study collected two waves of longitudinal data on socio-demographics, health practices, health outcomes and risk factors. The panel fixed-effects OLS regression models were used for the analysis.

Settings: The study was conducted in 59 villages and the town of Nouna with a population of about 100,000 individuals, one hospital and 13 primary health centres in Burkina Faso.

Participants: We interviewed 1,644 adolescents in 2017 and 1,291 respondents in 2018 who participated in both rounds.

Outcome and exposure measures: We examined the experience of physical attacks in the past 12 months and bullying in the past 30 days. Our exposures were completed years of age and educational attainment.

Results: A substantial minority of respondents experienced violence in both waves (24.1% bullying and 12.2% physical attack), with males experiencing more violence. Bullying was positively associated with more education (β=0.12; 95% confidence interval [CI]: 0.02, 0.22) and non-significantly with older age. Both effects were stronger in males than females, although the gender differences were not significant. Physical attacks fell with increasing age (β=-0.18; 95% confidence interval [CI]: -0.31, -0.05) and this association was again stronger in males than females; education and physical attacks were not substantively associated.

Conclusions: Bullying and physical attacks are common for rural adolescent Burkinabe. The age patterns found suggest that, particularly for males, there is a need to target violence prevention at younger ages and bullying prevention at slightly older ones, particularly for those remaining in school. Nevertheless, a fuller understanding of the mechanisms behind our findings is needed to design effective interventions to protect youth in low-income settings from violence.

For peer review only - http://bmjopen.bmj.com/site/about/guidelines.xhtml

For peer review only

Strengths and limitations of this Study:

- A key strength of our study is the fixed-effects design which robustly removes time-invariant confounding of effect measures.
- While fixed-effects analyses are powerful, they cannot control for unmeasured time-varying confounding and we may not have fully accounted for factors that change rapidly among adolescents.
- The use of self-reported data may have led to reporting biases.
- Generalizability from a sample drawn from a single district is always difficult to assess.
- Using a population-based sampling frame, our analysis provides results that represent the entire local population and are likely broadly applicable in poor, rural Burkina Faso and beyond.

INTRODUCTION

Violence globally represents both public health and an economic problem. In health terms, violence generates both mortality and morbidity. Violence is estimated to cause 1.3 million deaths annually, accounting for 2.5% of global mortality,[1]. Violence often requires acute health service access (e.g., assault victims requiring emergency hospital care) and can result in long-term physical disability, depression or reproductive health problems,[1, 2]. Violence also affects local economies through workforce absenteeism, loss of productivity and loss of human capital. Families can fall into poverty if a breadwinner dies or becomes permanently disabled due to violence,[1, 3].

Youth violence – which includes bullying, physical fighting, sexual and physical assault and homicide – is particularly problematic because it generates higher economic, welfare and criminal justice costs,[4]. Besides death, injury and psychological harm, youth violence can lead to increased subsequent health risks behaviours such as smoking, substance abuse, unsafe

For peer review only - http://bmjopen.bmj.com/site/about/guidelines.xhtml
For peer review only

sex and further violence,[5, 6]. An estimated 200,000 youth homicides occur each year, 83% among males, nearly all in low- and middle-income countries (LMICs), with particularly high rates in Latin America and the Caribbean (LAC) and sub-Saharan Africa [4]. In sub-Saharan Africa in particular, historical, economic and social factors continue to expose youth to violence,[7, 8]. Although violence affects all youth, adolescent girls and young women (AGYW) in LMICs appear to be most affected,[9-11]. Social and cultural norms, such as arranged and teenage marriage and denial of resource access to women, often keep AGYW economically dependent on males and thus vulnerable to abuse, especially from intimate partners,[12-14]. AGYW experience all forms of violence (emotional, physical and sexual), perpetrated by men and often other females in domestic and social settings [15-18].

Many factors have been proposed as determinants of youth violence receipt and perpetration in LMICs, often adopting or adapting Heise's integrated ecological framework for violence against women,[19, 20]. This framework conceptualizes violence as a multifaceted phenomenon grounded in an interplay among personal, situational and sociocultural factors. We modified this conceptualization to cover all forms of adolescent violence, concentrating on individual-level and microsystem (family/household/relationship) factors given our focus within a specific geography. We focus on two individual-level factors believed to play an important role in determining violence experience: age and education. Given the magnitude of youth violence, evidence of associations between violence, age and educational attainment, and calls for youth violence interventions,[1, 2], causal evidence on whether policies based on age or education are likely to affect violence levels is important. Past longitudinal analyses of adolescent violence often focused on the consequences of violence experience, rather than predictors of violence itself,[21, 22]. We instead focus on predictors of adolescent violence experience, to contribute to the upstream prevention of violence, rather than efforts to break the connection between violence and its sequelae.

For peer review only - http://bmjopen.bmj.com/site/about/guidelines.xhtml

Age is strongly associated with both the experience and perpetuation of violence,[3, 23]. Youth aged 15 to 29 are more likely to both experience and perpetrate violence than older adults, often experiencing violence perpetrated by their older peers or family members,[3, 23]. Males are more likely to perpetrate violence, while young girls and women are more likely to experience it,[9, 10, 14, 24, 25]. However, evidence on the causal effect of age on violence is comparatively scarce, with few even cross-sectional studies explicitly focused on youth,[3, 11, 26].

Education is theorized to protect against violence, since more-educated persons are less likely to either perpetrate or experience violence,[26, 27]. Evidence shows that women without education were 5.6 times more likely than those with college education to experience intimate partner violence (IPV). Similarly, wives of uneducated men were 1.84 times more likely than those whose husbands had college education to experience IPV. Even at the community level, the likelihood of IPV declined as community male and female literacy increased (after controlling for individual level factors) [28]. However, causal evidence on the effect of education on youth violence in sub-Saharan Africa is again limited. Two studies have used changes in national school policy as natural experiments in this context. One focused on violence, finding that a one-year increase in grade attainment was associated to a nine percentage-point reduction in the probability of experiencing sexual violence in Uganda, but no significant effect in Malawi,[27]. A second focused on sexual health, finding an additional year of schooling was associated with 0.11 fewer births and 14 percentage points less teen marriage in Ghana,[29]. Both of these studies necessarily assess the overall impact of policy change, rather than the increase in education alone, and it is unclear how their findings extrapolate to lower educational attainment settings.

For peer review only

Burkina Faso is a landlocked country in West Africa, which despite economic and political reforms remains one of the poorest in the world, with about half of its population living below the international poverty line,[30]. Economic deprivation is strongly associated with youth violence,[7, 31, 32]. The country is very young, with around 45% aged under fifteen and a further 20% aged 15-24 in 2015,[30, 33]. Educational access and youth literacy are limited, with only 13% of adults having completed primary education,[33, 34]. Within Burkina Faso, poverty is highest in the Boucle du Mouhoun region,[35]. Violence experience is common for young Burkinabe, with lifetime physical violence prevalence reported at 47-80% and sexual violence at 33-51%,[36, 37]. Adverse psychological and mental health outcomes commonly follow such experiences,[38, 39]. However, studies of Burkinabe youth violence have generally used cross-sectional designs and have not explored the effects of education or age specifically,[3, 40].

We therefore analysed longitudinal data on adolescents in Boucle de Mouhoun to assess the effects of age and education on violence experience. The potential for educational interventions to have violence-specific benefits in such high-poverty, low-education settings is likely to be substantial,[38, 39]. By using fixed-effects analysis we were able to exclude time-specific and time-invariant confounders, something particularly important given the many unobserved predictors of violence perpetration and victimization,[22, 41].

METHODS

Study design

We used data from the Nouna Health and Demographic Surveillance Site (HDSS) in north western Burkina Faso, which has been gathering demographic and epidemiological health information data since 1992. The 59 villages and the town of Nouna that comprise the HDSS

For peer review only - http://bmjopen.bmj.com/site/about/guidelines.xhtml
For peer review only

have a population of slightly over 100,000 individuals and include one hospital and 13 primary health centres (CSPS),[42].

Our study used longitudinal data from two Burkina Faso waves of the Africa Research, Implementation Science and Education (ARISE) adolescent health study,[43], collected in the Nouna HDSS in 2017 and 2018. Data were collected from 1644 adolescents aged 12-20 in 2017, based on a stratified random sample of 2544 age-eligible residents in Nouna town and ten villages,[43, 44]. A follow-up round was conducted in 2018, attempting to contact all those who participated in 2017; 1291 interviews were completed. In both years a standardized questionnaire was used to collect self-reported information on socio-demographics, health practices, health outcomes and risk factors. Data were collected by field staff with background in public health, medicine or a related field who had experience in conducting research and had general knowledge about local culture, health issues and the population under study. All study staff received in-depth training at the beginning of the study, covering the topic of research, human research ethics, the study protocol, questionnaire modules, electronic data entry and the procedures for implementing the study, including anthropometric evaluation.

Patient and Public Involvement

No patient involved.

Measures

We used two primary outcomes of youth violence, both captured as count variables: experience of physical attack in the past 12 months; and experience of bullying in the past 30 days (where bullying was defined as physical attacks, threats, insults, frequent nasty teasing, being left out on purpose or having rumors passed about them). We also generated binary measures of any experience for each outcome, in alignment with the World Report of Violence and Health's

For peer review only

definitions,[2]. Our exposures were age (in completed years) and education (years of fulltime education).

We additionally considered a range of time-varying covariates at the individual and household levels (based on Heise framework [20]), plus media use,[45, 46]. Specifically, our individual-level covariates were: currently in school; marital status (never married vs all else); any work in the past 12 months; and sexual behaviour (sexual debut and number of sexual partners). Our household-level covariates were: household size; household wealth quintile; parental vital status; parental support level (16 point scale, converted to the first principal component of the four variables included); parental co-residence (respondent lives with both parents, only mother, only father or lives alone); respondent has their own bedroom. Our media covariates were: any access to television; and frequency of watching television or reading magazines (never, rarely, often, very often). Full variable definitions are provided in Supplementary Table 1 and Supplementary Table 2.

Statistical analyses

We first described our data using frequency and percentages in both waves, including a comparison of those lost-to-follow-up vs those completing both waves. We then dropped any respondents who were missing data for the outcome variables, either due to preferring not to respond, not know their answer or where fieldwork errors affected responses, while for some the question was not applicable.

When considering the causal effect of education and age on youth violence, a major concern is unobserved confounding. Given the difficulty of implementing randomized controlled trials, since age is not directly manipulatable and intentional exposure to violence unethical,[47, 48], we exploited the panel nature of the data structure to run fixed-effects regression models to remove all time-invariant confounding. We specified our model as:

For peer review only - http://bmjopen.bmj.com/site/about/guidelines.xhtml

$$y_{it} = \alpha_i + \beta X_{it} + \gamma Z_{it} + \delta_t + \rho_i \qquad (1)$$

Where y_{it} is youth violence for each individual i at each time point t, X_{it} represents our time-varying exposures (education and age), Z_{it} is other time-varying factors for each individual, δ_t is a period-specific fixed effect to capture all individual-invariant factors and ρ_i are individual-specific fixed-effects which capture all time-invariant factors for each individual, e.g., gender, ethnicity, underlying proclivity to violence.

For each outcome (bullying and physical attack), we implemented three linear regression models of the count of reported events, i.e., assuming an observation-specific error structure $\epsilon_{it} \sim N(0,\sigma^2)$. We attempted to use Poisson and Negative Binomial models, i.e., modelling y_{it} as count data using a log-link and assuming that the variance of y_{it} is either equal to its mean (Poisson) or its mean plus a dispersion term (Negative Binomial), however neither model converged.

Model 1 considered only mean-centred age and years of full-time education. In model 2, we add all time-varying covariates. In model 3, we included interaction terms for gender with age and education to identify any gender-specific effects.

RESULTS

Description of sample

At baseline in 2017, 1,644 young people were interviewed, of whom 948 (57.7%) were male. By 2018, 21.5% of respondents, comprising 167 (24.0%) females and 186 (19.6%) males, were lost to follow up, leaving 1,291 respondents who participated in both rounds. We dropped 32 individuals (64 data points) who had missing values for the question on bullying, leaving 1,258 respondents for the bulling analysis. Similarly, we dropped 14 individuals (28 data points) who

For peer review only - http://bmjopen.bmj.com/site/about/guidelines.xhtml

did not answer physical attack question to arrive at 1,276 respondents. Figure 1 provides a flow chart of how the data were managed. We compared those who were and were not lost to follow up (Supplementary Table 3) and found only one significant difference with those who were retained, those not reinterviewed were less likely to be in school and had lower school attainment in wave 1.

[Figure 1 about Here]

The 1,291 respondents were aged 12 to 20 years in 2017: median 15.5, interquartile range (IQR): 14-18 (see Figure 2A). Around half were enrolled in school at each interview: 703 (54.5%) in 2017; 671 (52.0%) in 2018 (see Figure 2B).

[Figure 2A about Here]

[[Figure 2B about Here]

Around one-quarter of respondents never completed a year of fulltime education, while most others had at most attended primary or post-primary level. Over 90% of respondents were single at both interviews, and less than 20% in both 2017 and 2018 reported ever having sexual intercourse (Table 1). The proportion of respondents who ever worked fell from over 60% in 2017 to under 42% in 2018. Most respondents had living fathers (over 97%) and mothers (over 91%), however around one-quarter did not live with their parents. Media access was mixed: about 20% had no access to television in 2017 but around 15% watched several hours a day (access dropped by 2018); magazine reading was rare. Household wealth was by design evenly distributed across wealth quintiles. Households were large, with a median of 8 or 9 members.

Table 1. Descriptive statistics of independent variables (in percent)

	**Bullying sample**		**Physical attacks sample**	
	**2017**	**2018**	**2017**	**2018**
**N**	1253		1271	
Marital status				

For peer review only - http://bmjopen.bmj.com/site/about/guidelines.xhtml

Single vs. all other	90.7	89.6	90.8	89.6
Living situation				
Ever worked vs never	62.4	42.1	61.4	41.7
Mother is alive	97.6	96.8	97.7	97.0
Father is alive	91.7	89.7	91.9	89.8
Lives with mother	78.0	80.1	77.7	80.3
Lives with father	74.8	76.1	74.6	76.1
Lives alone	4.5	0.2	4.4	0.2
Has own bedroom	18.0	19.9	17.8	19.7
Household wealth quintile				
Lowest	19.0	20.8	19.8	21.0
Second lowest	19.5	20.4	20.0	20.7
Middle	22.4	19.0	22.1	19.0
Second highest	18.4	20.1	17.9	20.2
Highest	20.8	19.6	20.2	19.1
Education				
Currently in school	54.0	51.6	54.6	52.1
Highest school level				
None	46.1	48.4	45.5	47.9
Primary (1 – 6)	20.3	10.8	20.8	11.3
Post-primary (7 – 10)	29.9	33.6	30.1	33.7
Secondary (1 – 3)	2.6	3.9	2.6	3.9
University		0.1		0.1
Not applicable	1.1	3.2	1.1	3.2
Media use				
Has access to television	80.5	70.6	80.1	70.3
Frequency of watching television				
Never	20.1	30.8	20.5	31.0
Rarely (some hours per month)	22.8	22.8	22.7	22.8
Often (several hours per week)	42.1	36.2	41.8	36.1
Very often (several hours per day)	15.1	10.1	15.1	10.2
Frequency of reading magazines				
Never	90.9	87.1	91.1	87.3
Rarely (some hours per month)	2.4	8.5	2.23	8.4
Often (several hours per week)	2.4	4.1	2.4	3.9
Very often (several hours per day)	4.3	0.3	4.3	0.3
Sexual behaviour				
Ever had intercourse	17.3	19.4	15.8	18.9
Number of lifetime sexual partners				
1	9.7	13.5	9.4	13.3
2 – 7	3.8	5.2	3.8	5.3
8 – 17	0.3		0.3	
No response	2.1	1.3	2.4	0.3
Household size †	9 [6, 12]	8.5 [7, 11]	8.5 [6, 12]	8 [7, 11]
Parental support†	7 [4, 12]	10 [7, 13]	7 [4, 11]	10 [7, 13]

† depict medians and interquartile ranges. Samples are those with non-missing outcome responses for each of the two measures.

For peer review only - http://bmjopen.bmj.com/site/about/guidelines.xhtml

A substantial minority of respondents experienced bullying and physical attacks (Table 2). Overall, 189 females (18.9%) and 416 males (27.6%) experienced bullying in the 30 days preceding the interview, while 111 females (10.6%) and 199 males (13.2%) experienced physical attacks in the preceding 12 months. Across both rounds, males experienced both more violence than females: males experienced 416 of 605 (68.8%) unique bullying instances in the past 30 days, and 199 of 310 (64.2%) unique violence instances in the past 12 months. Bullying experience declined slightly with age – from over 26% among under 15s to 21% among over 18s; physical attacks fell more sharply, especially in early adolescence.

Table 2: Distribution of violence experience across observations by age and gender

Variable	N	Bullying (%)	N	Physical attacks (%)
Gender				
Female	996	189 (18.9)	1,038	111 (10.6)
Male	1,510	416 (27.6)	1,504	199 (13.2)
Age				
12	19	52 (26.1)	210	51 (24.3)
13	387	100 (25.8)	398	73 (18.3)
14	389	104 (26.7)	403	65 (16.1)
15	327	76 (23.2)	331	30 (9.1)
16	286	67 (23.4)	288	22 (7.6)
17	274	64 (23.4)	272	27 (9.9)
18	297	63 (21.2)	295	20 (6.8)
19	214	43 (20.1)	212	15 (7.1)
20	130	35 (26.9)	130	7 (5.4)
21	3	1 (33.3)	3	0 (0.0)
Total	2506	605 (23.8)	2542	310 (12.2)

Note: Each individual is represented twice in this table, once per survey round.

For peer review only

Fixed-effects analysis

In bivariate fixed-effects regression, older age was associated with non-significantly more bullying (0.14 more bullying experiences per month for each additional year of age, 95% confidence interval [CI]: -0.12, 0.39) and associated with significantly fewer physical attacks (-0.19, 95%CI: -0.32, -0.06). More education was associated with significantly more bullying (0.11 bullying experiences per month for each additional year of schooling, 95%CI: 0.01, 0.21),

but not with physical attacks (Table 3, Model 1). Controlling for time-varying potential confounders attenuated the association of age and bullying but otherwise had limited effects on our relationship of interest (Table 3, Model 2). When we allowed effects to vary by gender (Table 3, Model 2), associations in all four models were more positive for men than for women, with wider gaps for the impact of age on both outcomes than for education. All these results were independent of a substantial but imprecise negative association between currently being in school and bullying or attacks.

Table 3: OLS models predicting count of violence events

	Model 1<br>Unadjusted	Model 2<br>Adjusted	Model 3<br>Adjusted & interaction	Interaction test
Bullying in the last 30 days (N=2506)				
Age in years	0.14 [-0.12, 0.39]	0.08 [-0.17, 0.33]		
Female			-0.08 [-0.39, 0.24]	
Male			0.18 [-0.10, 0.45]	F=2.76, p=0.10
Fulltime education in years	0.11 [0.01, 0.21]	0.12 [0.02, 0.22]		
Female			0.07 [-0.11, 0.26]	
Male			0.14 [0.03, 0.26]	F=0.39, p=0.53
Currently in school		-0.43 [-1.05, 0.19]	-0.41 [-1.03, 0.21]	
Physical attacks in the last 12 months (N=2542)				
Age in years	-0.19 [-0.32, -0.06]	-0.18 [-0.31, -0.05]		
Female			-0.10 [-0.26, 0.05]	
Male			-0.23 [-0.38, -0.09]	F=2.74, p=0.10
Fulltime education in years	0.02 [-0.03, 0.07]	0.04 [-0.01, 0.09]		
Female			0.06 [-0.03, 0.16]	
Male			0.03 [-0.03, 0.09]	F=0.41, p=0.52
Currently in school		-0.27 [-0.59, 0.05]	-0.27 [-0.59, 0.04]	

The table presents four regression models per column, all showing point estimates and 95% confidence intervals. All models contain fixed-effects for each respondent and interview round. Age centred at 15. Models 2 and 3 are also adjusted for marital status, ever worked, household size, parent alive, living with parents, household wealth, has own bedroom and access to media; full results available in Supplementary Table 4.

DISCUSSION

In this study we employed individual and time fixed-effects models to assess the effects of age and education on violence experience in the form of bullying and physical attacks among adolescents and young adults in a panel study in rural Burkina Faso. We found bullying

For peer review only - http://bmjopen.bmj.com/site/about/guidelines.xhtml

experience prevalence in the past 30 days ranging from 20-30%, and physical attack experience of 10-15% in the previous 12 months. While there is little directly comparable data in Burkina Faso, both levels seem concerning, if in line with studies elsewhere,[3, 40]. In fixed-effects models we found bullying was associated with more education and weakly with greater age, both effects stronger in males, while physical attacks were associated with younger age (again more strongly for males) but not with education.

Our findings on the effect of age on youth experience of physical attacks are consistent with some observational evidence elsewhere, for example physical violence from both peers and caregivers falls with age in Latin America and the Caribbean,[40]. Evidence on the causal effect of age on violence is rare, with even cross-sectional studies focused on youth uncommon,[3, 11, 26]. Studies of intimate partner violence (IPV) amongst women suggest rates are higher in older teenage girls compared to adults,[49, 50], but this does not allow within-adolescence comparisons. The faster decline with age that we see for males (from a higher initial level) is important to note: while criminal interpersonal violence appears peaks around age 18 in many settings, our findings and past work suggest that overall frequency of violence experience may in fact decline across teenage years, at least in non-urban settings,[51]. In combination, this evidence suggests a shift in violence experience composition for adolescent males that would be worth further investigation.

The implications of this negative association between age and violence experience in adolescence depend on what mechanisms are generating them. First, age might be a proxy for predictors of violence that we have not captured in this analysis. This might include adolescent autonomy in decision-making, for example relating to bedtime and the amount and type of television watched, which typically rises with age,[52]: adolescent autonomy was negatively associated with youth violence among US Latino youth,[53]. Alternatively, age might be a

For peer review only - http://bmjopen.bmj.com/site/about/guidelines.xhtml

distal determinant of factors leading more directly to violence experience. For example, older adolescents may be better able to protect themselves against aggressive behaviour from their peers or adults. If this is the case, then structural interventions (at the family, community or national levels) or behaviour change interventions (e.g., teaching adolescents how to avoid confrontations) might be beneficial in protecting younger adolescents,[54, 55]. Further research to understand these causal mechanisms, and thus design effective interventions, will need to include more detailed quantitative data on who perpetrates violence against younger adolescents and qualitative information on how and why such violence comes about.

Our finding that bullying increases with age contradicts some past research. Observationally, bullying victimization rates are higher in younger children than in older adolescents, both in the US and in sub-Saharan Africa,[56, 57]. Our results may reflect the stronger control our fixed-effects approach provides against between-individual and temporal confounding, suggesting that the decline in bullying seen elsewhere is a function of factors associted with age, rather than age itself. Our finding of a stronger association for males adds to a mixed literature, aligning with studies from Taiwan and Saskatchwan Canada,[58, 59], but in contrast to findings from the US and Manitoba Canada,[58, 60].

We found that education was not associated with violence experience in our setting. A similar null effect of education (grade attainment) was reported for 19-31 year old Malawian women,[27]. A recent meta-analysis of 86 studies in 60 LMICs noted that poor academic performance and weak school attachment were correlated with increased youth violence,[23], in contrast to our null findings. Again, more detail is available for IPV, with a Ugandan study finding that less-educated women were more likely to experience physical IPV – however this study included women aged 15-49, which makes direct comparison difficult,[61]. Other studies have confirmed the protective effect of education on violence in different settings,[49, 62]. The

For peer review only - http://bmjopen.bmj.com/site/about/guidelines.xhtml

discrepancy between others' findings and ours may reflect the majority of past studies being cross-sectional, while we were able to use panel data. It may also reflect different exposures, since we considered quantity (years of schooling) rather than quality (performance or attachment).

Past evidence on the effect of education on bullying is mixed, with several studies finding lower bullying amongst those with more education,[57, 58] and few finding the opposite,[60]. Our finding of a positive association between education and bullying in both males and females may reflect the a true causal association, or the residual effect of being in school – given the opportunities that this provides for bullying relative to the alternative settings of field-based work or animal herding.

Finally, our analysis covers a population where half of adolescents are not now, and one-quarter never have been, attending school. The role of education in promoting or protecting against violence at the community level may be different in settings where education is not even close to universal. Further investigation of why our results looks different from other settings, including qualitative study of social norms surrounding violence across levels of educational attainment, would be instructional.

For peer review only

Strengths and limitations

A key strength of our study is the fixed-effects design which robustly removes time-invariant confounding of effect measures,[63]. Nevertheless, our study also has potential limitations: while fixed-effects analyses are powerful, they cannot control for unmeasured time-varying confounding and we may have therefore not fully accounted for factors that change rapidly among adolescents, such as increased social media access or social network change. The use of self-reported data may have led to reporting biases, although these would have had to vary differentially over time within respondents in order to bias our fixed-effects analyses.

For peer review only - http://bmjopen.bmj.com/site/about/guidelines.xhtml

Generalizability from a sample drawn from a single district is always difficult to assess. However, by using a population-based sampling frame our analysis provides results that represent the entire local population and are likely to be broadly applicable in poor, rural settings in Burkina Faso and beyond.

Conclusion

A substantial minority of adolescents in rural north-western Burkina Faso report recent experiences of bullying or physical attack. We hypothesised years of education received and age would be associated with violence experience in Burkina Faso. Our findings show the prevalence of these experiences was not significantly associated with years of education received, even within individual respondents, but did fall with age. However, our study was not able to identify mechanisms behind these associations, and we therefore recommend a mixed method study that includes study of household dynamics to move beyond an individualized understanding of violence amongst adolescents. Such an understanding is central to designing interventions to better protect youth in low-income settings from violence.

For peer review only

Contributorship statement

NK: Conceptualization, Methodology, Formal analysis, Writing – original draft. **MB**: Investigation, Writing – Review & Editing, Project administration. **LO**: Investigation, Writing – Review & Editing, Project administration. **AS**: Investigation, Resources, Writing – Review & Editing, Supervision. **TB**: Conceptualization, Methodology, Writing – Review & Editing, Project administration, Supervision, Funding acquisition. **GH**: Methodology, Data curation, Formal analysis, Writing – Review & Editing, Supervision, Project administration. All authors contributed revisions to the text, approved the final version and agree to be accountable for the work.

Competing interest

Authors declare no competing interests.

Funding

Funding for the study on which these data are based is provided by the Alexander von Humboldt Foundation through an award to Professor Bärnighausen. GH was supported by a fellowship from the Royal Society and the Wellcome Trust [Grant number 210479/Z/18/Z]. This research was funded in whole, or in part, by the Wellcome Trust [Grant number 210479/Z/18/Z]. For the purpose of open access, the authors have applied a CC BY public copyright licence to any Author Accepted Manuscript version arising from this submission.

Data sharing statement

The data that support the findings of this study are available from the corresponding author, TB, upon reasonable request and after signing a data use agreement. The data are not publicly available due to the risk of participant re-identification given the large amount of information contained for each person.

Ethical considerations

The Nouna ARISE study was approved by CRSN's Institutional Ethics Committee (2017/08 and 2018/017) and Heidelberg Medical Faculty's Ethics Commission (S-607/2018). Secondary

For peer review only - http://bmjopen.bmj.com/site/about/guidelines.xhtml

data analysis was approved by University College London's Research Ethics Committee (15231/005). Prior to the study village elder provided oral assent. Each participants provided written informed consent; for respondents aged under 18, parents or guardians provided written consent alongside the minor's written assent. In cases of illiteracy, a literate witness assisted.

For peer review only

For peer review only - http://bmjopen.bmj.com/site/about/guidelines.xhtml

References

1. World Health organization, *Global Status report on Viloence Prevention*. 2014.

2. Krug, E.G., et al., *The world report on violence and health*, in *Lancet*. 2002. p. 1083-8.

3. Golshiri, P., et al., *Youth Violence and Related Risk Factors: A Cross-sectional Study in 2800 Adolescents.* Adv Biomed Res, 2018. **7**: p. 138.

4. World Health Organization, *Preventing youth violence: an overview of the evidence*. 2015.

5. Bellis, M.A., et al., *Adverse childhood experiences and associations with health-harming behaviours in young adults: surveys in eight eastern European countries.* Bulletin of the World Health Organization, 2014. **92**: p. 641-655.

6. World Health Organization. *Youth Violence*. 2019; Available from: https://www.who.int/health-topics/violence-against-children#tab=tab_3.

7. Ward, C.L., A. Dawes, and R. Matzopoulos, *Youth violence in South Africa: Setting the scene*, in *Youth violence: Sources and Solutions in South Africa*, C.L. Ward, A. Van der Merwe, and A. Dawes, Editors. 2013, University of Cape Town Press: Cape Town. p. 1-20.

8. Lamb, G., *Social cohesion and violence in South Africa: constructing a puzzle with missing pieces.* Crime, Law and Social Change, 2019. **72**(4): p. 365-385.

9. Rumble, L., et al., *Childhood sexual violence in Indonesia: a systematic review.* Trauma, Violence and Abuse, 2020. **21**(2): p. 284-299.

10. Iman'ishimwe Mukamana, J., P. Machakanja, and N.K. Adjei, *Trends in prevalence and correlates of intimate partner violence against women in Zimbabwe, 2005-2015.* BMC Int Health Hum Rights, 2020. **20**(1): p. 2.

For peer review only - http://bmjopen.bmj.com/site/about/guidelines.xhtml

11. Malta, D.C., et al., *Factors associated with family violence against adolescents based on the results of the National School Health Survey (PeNSE).* Cien Saude Colet, 2019. **24**(4): p. 1287-1298.

12. World Health Organization, *World Bank Group Gender Strategy (FY16-23) : Gender Equality, Poverty Reduction and Inclusive Growth*. 2015: World Bank, Washington, DC. © World Bank. .

13. Shamu, S., et al., *A systematic review of African studies on intimate partner violence against pregnant women: prevalence and risk factors.* PLoS One, 2011. **6**(3): p. e17591.

14. Girmay, A., et al., *Intimate partner physical violence and associated factors in reproductive age married women in Aksum Town, Tigray, Ethiopia 2018, and community based study.* BMC Res Notes, 2019. **12**(1): p. 627.

15. Breiding MJ, S.S., Basile KC, Walters ML, Chen J, & Merrick MT, *Prevalence and characteristics of sexual violence, stalking, and intimate partner violence victimization—National Intimate Partner and Sexual Violence Survey, United States, 2011.* Morbidity and Mortality Weekly Report, 2014. **63**(8): p. 1-18.

16. Baigorria, J., et al., *[Prevalence and associated factors with sexual violence against women: systematic review].* Rev Salud Publica (Bogota), 2017. **19**(6): p. 818-826.

17. Belay, S., et al., *Intimate partner violence and maternal depression during pregnancy: A community-based cross-sectional study in Ethiopia.* PLoS One, 2019. **14**(7): p. e0220003.

18. Semahegn, A. and B. Mengistie, *Domestic violence against women and associated factors in Ethiopia; systematic review.* Reprod Health, 2015. **12**: p. 78.

19. Puri, M., et al., *The prevalence and determinants of sexual violence against young married women by husbands in rural Nepal.* BMC research notes, 2012. **5**(1): p. 291.

20. Heise, L.L., *Violence against women: an integrated, ecological framework.* Violence Against Women, 1998. **4**(3): p. 262-90.

For peer review only - http://bmjopen.bmj.com/site/about/guidelines.xhtml

21. Ttofi, M.M., et al., *Do the victims of school bullies tend to become depressed later in life? A systematic review and meta-analysis of longitudinal studies.* Journal of Aggression, Conflict and Peace Research, 2011. **3**(2): p. 63-73.

22. Valido, A., et al., *Intra-familial Violence and Peer Aggression Among Early Adolescents: Moderating Role of School Sense of Belonging.* Journal of Family Violence, 2020. **36**: p. 87-98.

23. de Ribera, O.S., et al., *Correlates of youth violence in low-and middle-income countries: A meta-analysis.* Aggression and Violent Behavior, 2019. **49**: p. 101306.

24. Mason-Jones, A.J., et al., *Intimate partner violence in early adolescence: The role of gender, socioeconomic factors and the school.* South African Medical Journal, 2016. **106**(5): p. 502-509.

25. Masuda, K. and C. Yamauchi, *How Does Female Education Reduce Adolescent Pregnancy and Improve Child Health?: Evidence from Uganda's Universal Primary Education for Fully Treated Cohorts.* The Journal of Development Studies, 2020. **56**(1): p. 63-86.

26. Obradovic-Tomasevic, B., et al., *Prevalence and Predictors of Violence Victimization and Violent Behavior among Youths: A Population-Based Study in Serbia.* Int J Environ Res Public Health, 2019. **16**(17): p. 3203.

27. Behrman, J.A., A. Peterman, and T. Palermo, *Does Keeping Adolescent Girls in School Protect Against Sexual Violence? Quasi-Experimental Evidence From East and Southern Africa.* Journal of Adolescent Health, 2017. **60**(2): p. 184-190.

28. Ackerson, L.K., et al., *Effects of individual and proximate educational context on intimate partner violence: a population-based study of women in India.* American journal of public health, 2008. **98**(3): p. 507-514.

29. Adu Boahen, E. and C. Yamauchi, *The effect of female education on adolescent fertility and early marriage: Evidence from free compulsory universal basic education in Ghana.* Journal of African Economies, 2018. **27**(2): p. 227-248.

For peer review only

For peer review only - http://bmjopen.bmj.com/site/about/guidelines.xhtml

30. World Health Organization. *WHO Country Cooperation Strategy at a glance: Burkina Faso*. 2016 [cited 2019 19/03/2019]; Available from: https://apps.who.int/iris/handle/10665/136973.

31. de Ribera, O.S., et al., *Correlates of youth violence in low-and middle-income countries: A meta-analysis.* 2019. **49**: p. 101306.

32. van der Merwe, A. and A. Dawes, *Youth violence: A review of risk factors, causal pathways and effective intervention.* Journal of Child and Adolescent Mental Health, 2007. **19**(2): p. 95-113.

33. World Health Organization. *World Health Statistics 2015*. 2015 [cited 2018 10/06/2018]; Available from: https://www.who.int/gho/publications/world_health_statistics/2015/en/.

34. World Health Organization, *Country Cooperation Strategy at a glance: Burkina Faso*. 2016.

35. Burkina Faso Mininstere de L'Economie des Finances et du Development Secretariat General, *Tableau de Bord Social du Burkina Faso*. 2017, INSTITUT NATIONAL DE LA STATISTIQUE ET DE LA DEMOGRAPHIE

36. Wirtz, A.L., et al., *Sexual violence, condom negotiation, and condom use in the context of sex work: results from two West African countries.* J Acquir Immune Defic Syndr, 2015. **68 Suppl 2**: p. S171-9.

37. Ouédraogo, S.Y.Y.A., E.J. Sisawo, and S.-L. Huang, *Sexual abuse and risky sexual behaviors among young female hawkers in Burkina Faso: a mixed method study.* BMC international health and human rights, 2017. **17**(1): p. 1.

38. Ismayilova, L., et al., *Maltreatment and mental health outcomes among ultra-poor children in Burkina Faso: A latent class analysis.* Plos One, 2016. **11**(10): p. e0164790.

39. Ismayilova, L. and L. Karimli, *Harsh parenting and violence against children: a trial with ultrapoor families in Francophone West Africa.* Journal of Clinical, Child and Adult psychology, 2020. **49**(1): p. 18-35.

For peer review only - http://bmjopen.bmj.com/site/about/guidelines.xhtml

40. Devries, K., et al., *Violence against children in Latin America and the Caribbean: What do available data reveal about prevalence and perpetrators?* Revista panamericana de salud publica, 2019. **43**: p. e66.

41. Farrington, D.P. and M.M. Ttofi, *Advancing Knowledge about Youth Violence: Child Maltreatment, Bullying, Dating Violence, and Intimate Partner Violence.* Journal of Family Violence, 2020. **36**(1): p. 109-115.

42. Sié, A., et al., *The Health and Demographic Surveillance System (HDSS) in Nouna, Burkina Faso, 1993-2007.* Glob Health Action, 2010. **3**(1).

43. Darling, A.M., et al., *Design and field methods of the ARISE Network Adolescent Health Study.* Journal of Tropical Medicine and International Health, 2020. **25**(1): p. 5-14.

44. Greis, A., et al., *Attitudes towards female genital cutting among adolescents in rural Burkina Faso: A multilevel analysis Female genital cutting in Burkina Faso.* Journal of Tropical Medicine and International Health, 2019. **25**(1): p. 119-131.

45. Bushman, B.J., et al., *Youth violence: What we know and what we need to know.* American Psychologist, 2016. **71**(1): p. 17.

46. Tripathi, V., *Youth violence and social media.* Journal of Social Sciences, 2017. **52**(1-3): p. 1-7.

47. Khandker, S.R., G.B. Koolwal, and H.A. Samad, *Handbook on impact evaluation: quantitative methods and practices*. 2010: World Bank Publications.

48. Gertler, P.J., et al., *Impact Evaluation in Practice*. Second ed. 2016, Washington D.C: International Bank for Reconstruction and Development/The World Bank.

49. Abramsky, T., et al., *What factors are associated with recent intimate partner violence? Findings from the WHO multi-country study on women's health and domestic violence.* BMC public health, 2011. **11**(1): p. 1-17.

For peer review only - http://bmjopen.bmj.com/site/about/guidelines.xhtml

50. Black, E., et al., *Prevalence and correlates of intimate partner violence against women in conflict affected northern Uganda: a cross-sectional study.* Confl Health, 2019. **13**: p. 35.

51. Fahlgren, M.K., et al., *Age and gender effects in recent violence perpetration.* Journal of interpersonal violence, 2020. **35**(17-18): p. 3513-3529.

52. Tashjian, S.M., J.L. Mullins, and A. Galván, *Bedtime autonomy and cellphone use influence sleep duration in adolescents.* Journal of Adolescent Health, 2019. **64**(1): p. 124-130.

53. Estrada-Martínez, L.M., et al., *Families, Neighborhood Socio-Demographic Factors, and Violent Behaviors among Latino, White, and Black Adolescents.* Youth & Society, 2011. **45**(2): p. 221-242.

54. Kellermann, A.L., et al., *Preventing youth violence: what works?* Annual review of public health, 1998. **19**(1): p. 271-292.

55. Botvin, G.J., K.W. Griffin, and T.D. Nichols, *Preventing youth violence and delinquency through a universal school-based prevention approach.* Prevention science, 2006. **7**(4): p. 403-408.

56. Lebrun-Harris, L.A., et al., *Bullying victimization and perpetration among US children and adolescents: 2016 National Survey of Children's Health.* Journal of Child and Family Studies, 2019. **28**(9): p. 2543-2557.

57. Aboagye, R.G., et al., *A multi-country analysis of the prevalence and factors associated with bullying victimisation among in-school adolescents in sub-Saharan Africa: evidence from the global school-based health survey.* BMC Psychiatry, 2021. **21**(1): p. 325.

58. Ashrafi, A., et al., *Bullying victimization among preadolescents in a community-based sample in Canada: a latent class analysis.* BMC research notes, 2020. **13**(1): p. 138.

59. Hokoda, A., H.-H.A. Lu, and M. Angeles, *School bullying in Taiwanese adolescents.* Journal of Emotional Abuse, 2006. **6**(4): p. 69-90.

For peer review only - http://bmjopen.bmj.com/site/about/guidelines.xhtml

60. Salmon, S., et al., *Bullying victimization experiences among middle and high school adolescents: Traditional bullying, discriminatory harassment, and cybervictimization.* Journal of Adolescence, 2018. **63**: p. 29-40.

61. Amegbor, P.M. and L. Pascoe, *Variations in Emotional, Sexual, and Physical Intimate Partner Violence Among Women in Uganda: A Multilevel Analysis.* J Interpers Violence, 2019. **36**(15-16): p. NP7868-NP7898.

62. Cofie, N., *A multilevel analysis of contextual risk factors for intimate partner violence in Ghana.* international Review of Victimology, 2020. **26**(1): p. 50-78.

63. Imai, K. and I.S. Kim, *When should we use unit fixed effects regression models for causal inference with longitudinal data?* American Journal of Political Science, 2019. **63**(2): p. 467-490.

For peer review only

For peer review only - http://bmjopen.bmj.com/site/about/guidelines.xhtml

Figure 1: Flow chart of sample

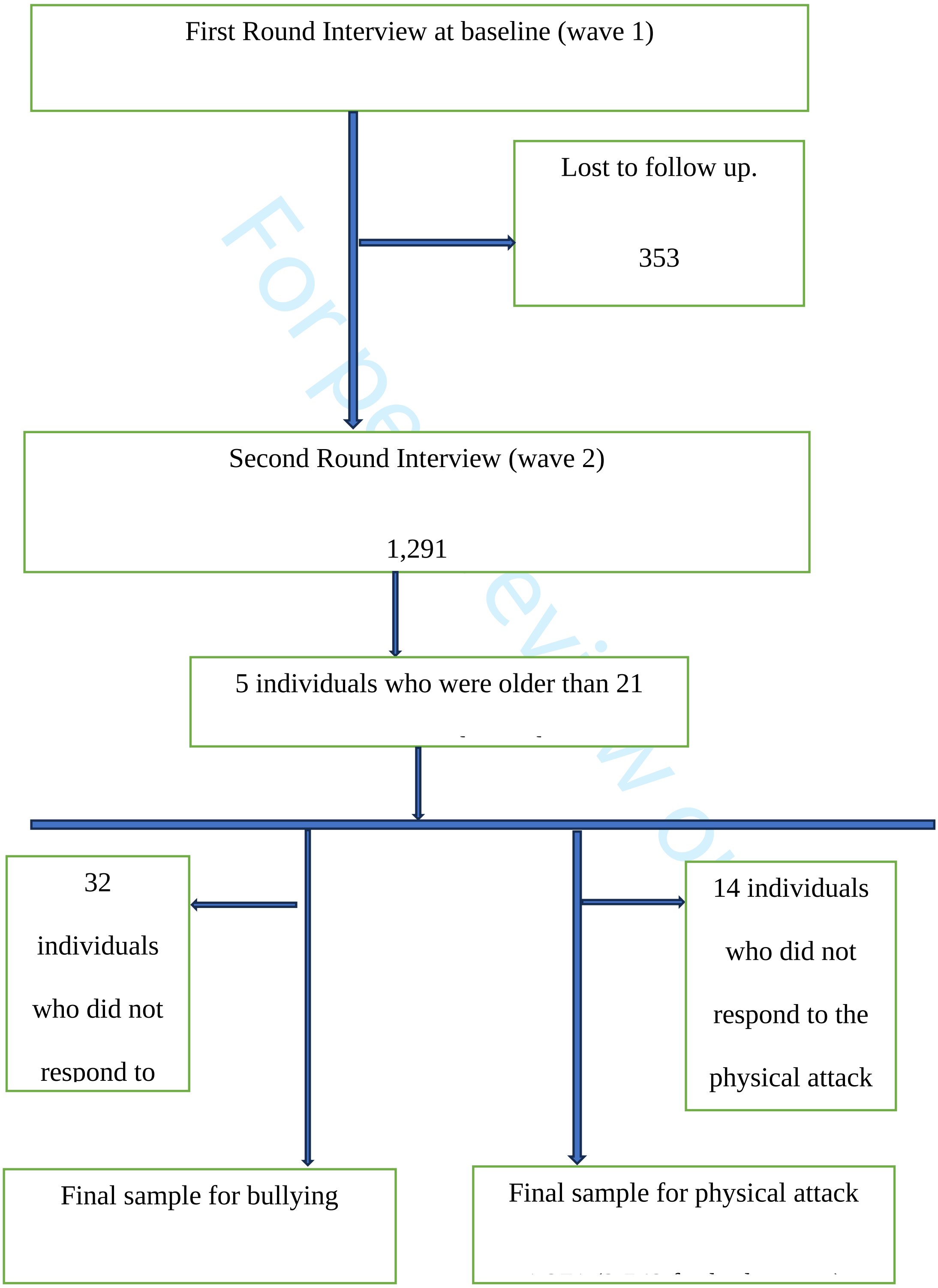

Figure 2. Exposure distribution among respondents

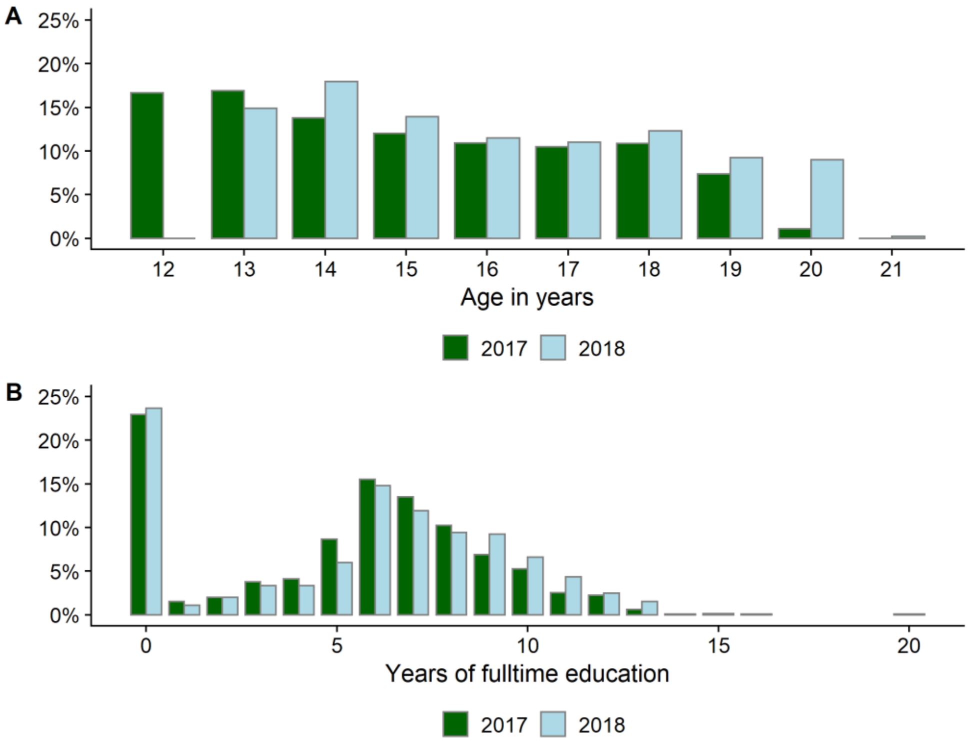

For peer review only - http://bmjopen.bmj.com/site/about/guidelines.xhtml

SUPPLEMENTARY MATERIAL

Title: The effects of education and age on experience of youth violence in a very low-income setting: a fixed-effects analysis from rural Burkina Faso

Supplementary Table 1a: Variables and their measurements

	Measurement
Outcome	
Violence experience (VE1)	Ever experienced bullying, physical attack or unwelcome sexual advances in the last 6 months
Bullying	Yes / No
Physical attack	Yes / No
Sexual violence	Yes / No
Violence perpetration (VP1)	Ever perpetrated bullying or physical attack in the last 6 months
Bullying	Yes / No
Physical attack	Yes / No
Individual level factors	
Age	Age in complete years
Education	
Level of schooling	Primary, post-primary or secondary level
School attainment	Grade/class/standard
Years of schooling	Years of education completed in years
Highest level of schooling	Primary, post-primary, secondary, technical/vocational or university
Marital status	Single, engaged, married (monogamous), married (polygamous), civil union, separated/divorced or widowed
Experienced first sex	Had first sex in the last 6 months (Yes / No)
Multiple sex partners	Had sex with more than one person in your life time (Yes / No)
Number of sexual partners	Number of life time sexual partners
Employment	Done any work in the last 12 months to earn money or obtain food (Yes/ No)
Alcohol use	Ever drunk alcohol on your own (Yes / No)
Substance use	Ever used drugs (Yes / No)
Household level factors	
Household asset	Asset index for household
Household size	Number of persons living in household
Aged 0 – 14 years	
Aged 15 – 29 years	
Aged 30 – 44 years	
Aged 45 – 59 years	
Aged 60 and above	
Moving between household	Sometimes live in another household other than primary household (Yes / No)
Living with adult figure	Yes / No
Adult figure	Father / Mother
Age of adult figure	Age in complete years

For peer review only - http://bmjopen.bmj.com/site/about/guidelines.xhtml

For peer review only

Less than 30 years	
30 – 45 years	
46 – 60 years	
Above 60 years	
No adult figure	
Educational level adult figure Male Female	Primary, post-primary, secondary, technical/vocational or university
Occupation of adult figure Male Female	Farmer, merchant, teacher, other government worker, other (specified)
Frequency of parental /guardian support	In the last 30 days how often did parent/ guardian……
With important life decisions	Helped you make important decisions (never, rarely, sometimes, most of the times or always)
With understanding problems	Understand your problems and worries (never, rarely, sometimes, most of the times or always)
Talking about what happens	Take time to talk with you about things that happen to you (never, rarely, sometimes, most of the times or always)
Paying attention to opinion	Pay attention to your opinion (never, rarely, sometimes, most of the times or always)
Media use	
Access to media	
Radio	Yes / No
TV	Yes / No
Magazines	Yes / No
Use of media	
Radio	Average number of hours per day spent listening to radio
TV_1	Frequency of watching TV [never, rarely (some hours per month), often (some hours per week) or very frequently (several hours per day)]
TV_2	Average number of hour per day spent watching TV
Magazines	How often do you read magazines [never, rarely (some hours per month), often (some hours per week) or very frequently (several hours per day)]
Community level factor	
Access to education	Community share of school enrolment

For peer review only - http://bmjopen.bmj.com/site/about/guidelines.xhtml

Supplementary Table 2: Parental support (n=1291)

Variable	2017		2018	
	Frequency	Percent	Frequency	Percent
Parents understand worries				
Never	299	23.27	68	5.29
Rarely	201	15.64	193	15.01
Sometimes	271	21.09	391	30.40
Most of the time	159	12.37	295	22.94
Always	351	27.32	339	26.36
Missing	4	0.31		
Parents help make decisions				
Never	212	16.50	27	2.10
Rarely	257	20.00	193	15.01
Sometimes	311	24.20	350	27.22
Most of the time	159	12.37	331	25.74
Always	337	26.23	384	29.86
Missing	9	0.70	1	0.08
Parents talk about things that happen				
Never	299	23.27	83	6.45
Rarely	253	19.69	268	20.84
Sometimes	324	25.21	333	25.89
Most of the time	161	12.53	340	26.44
Always	243	18.91	262	20.37
Missing	5	0.39		
Parents pay attention to what you say				
Never	317	24.67	94	7.31
Rarely	296	23.04	225	17.50
Sometimes	381	29.65	416	32.35
Most of the time	133	10.35	236	18.35
Always	153	11.91	314	24.42
Missing	5	0.39	1	0.08

We built a summative score of parental support by adding the four variables, with a minimum score of 0 and a maximum score of 16.

For peer review only

For peer review only - http://bmjopen.bmj.com/site/about/guidelines.xhtml

Supplementary Table 3: Difference between those who dropped out in wave 2 and remaining sample.

	participation_w2 = 0	participation_w2 = 1	Test	Statistic	p-value
	N=353	N=1,291			
Number of physical attacks, last 12m	0 (0-0)	0 (0-0)	Wilcoxon rank-sum	Z= -0.74	0.46
Days bullied, last 30d	0 (0-0)	0 (0-0)	Wilcoxon rank-sum	Z= -1.18	0.24
Gender			Chi-square	Chi2(1)= 4.55	0.033
Female	167 (47.3%)	529 (41.0%)			
Male	186 (52.7%)	762 (59.0%)			
Age (years)	15 (14-18)	15 (13-17)	Wilcoxon rank-sum	Z= 3.64	<0.001
Marital status cat			Chi-square	Chi2(1)= 2.08	0.15
other	*41 (11.6%)*	*117 (9.1%)*			
single	*312 (88.4%)*	*1,174 (90.9%)*			
Ever worked	222 (62.9%)	786 (60.9%)	Chi-square	Chi2(1)= 0.47	0.49
Mother is alive	342 (96.9%)	1,261 (97.7%)	Chi-square	Chi2(1)= 0.72	0.40
Father is alive	316 (89.5%)	1,186 (91.9%)	Chi-square	Chi2(1)= 1.94	0.16
Lives with Mother	262 (74.2%)	1,005 (77.8%)	Chi-square	Chi2(1)= 2.06	0.15
Lives with father	245 (69.4%)	965 (74.7%)	Chi-square	Chi2(1)= 4.07	0.044
Lives alone	23 (6.5%)	56 (4.3%)	Chi-square	Chi2(1)= 2.87	0.090
Has own bedroom	58 (16.4%)	228 (17.7%)	Chi-square	Chi2(1)= 0.29	0.59
Wealth quintile			Chi-square	Chi2(4)= 1.12	0.89
1	*76 (21.5%)*	*255 (19.8%)*			
2	*74 (21.0%)*	*257 (19.9%)*			
3	*73 (20.7%)*	*282 (21.8%)*			
4	*64 (18.1%)*	*235 (18.2%)*			
5	*66 (18.7%)*	*262 (20.3%)*			
Currently in school	119 (33.7%)	703 (54.5%)	Chi-square	Chi2(1)= 47.71	<0.001
Current school level			Chi-square	Chi2(4)= 53.56	<0.001
None	*234 (67.2%)*	*589 (46.2%)*			
Primary (1-6)	*40 (11.5%)*	*268 (21.0%)*			
Post Primary (7-10)	*66 (19.0%)*	*386 (30.3%)*			
Secondary (1-3)	*7 (2.0%)*	*33 (2.6%)*			
Technical/Vocational	*1 (0.3%)*	*0 (0.0%)*			
Has access to TV	295 (83.6%)	1,035 (80.2%)	Chi-square	Chi2(1)= 2.07	0.15
Frequency of watching TV			Chi-square	Chi2(3)= 2.69	0.44
Never	*59 (16.8%)*	*263 (20.4%)*			
Rarely (some hours per month)	*88 (25.0%)*	*290 (22.5%)*			

For peer review only

Often (several hours per week)	*151 (42.9%)*	*540 (41.9%)*			
Very often (several hours per day)	*54 (15.3%)*	*197 (15.3%)*			
Frequency of reading magazines			Chi-square	Chi2(3)= 6.12	0.11
Never	*334 (94.6%)*	*1,177 (91.2%)*			
Rarely (some hours per month)	*8 (2.3%)*	*30 (2.3%)*			
Often (several hours per week)	*5 (1.4%)*	*30 (2.3%)*			
Very often (several hours per day)	*6 (1.7%)*	*54 (4.2%)*			
Ever had intercourse?	*55 (16.8%)*	*202 (17.0%)*	*Chi-square*	*Chi2(1)= 0.01*	*0.93*
Number of sex partners, lifetime			Chi-square	Chi2(11)= 6.01	0.87
0	*298 (86.4%)*	*1,087 (86.3%)*			
1	*29 (8.4%)*	*121 (9.6%)*			
2	*9 (2.6%)*	*25 (2.0%)*			
3	*5 (1.4%)*	*14 (1.1%)*			
4	*1 (0.3%)*	*3 (0.2%)*			
5	*0 (0.0%)*	*4 (0.3%)*			
6	*1 (0.3%)*	*1 (0.1%)*			
7	*0 (0.0%)*	*1 (0.1%)*			
8	*1 (0.3%)*	*1 (0.1%)*			
9	*0 (0.0%)*	*1 (0.1%)*			
10	*1 (0.3%)*	*1 (0.1%)*			
17	*0 (0.0%)*	*1 (0.1%)*			
Scores for component 1	-.2998273 (-1.694384-.8893526)	-.6083845 (-1.707631-.9265742)	Wilcoxon rank-sum	Z= 0.57	0.57
Parental_support	2 (1-2.75)	1.75 (1-2.75)	Wilcoxon rank-sum	Z= 0.71	0.48

Data are presented as median (IQR) for continuous measures, and n (%) for categorical measures.

For peer review only

For peer review only - http://bmjopen.bmj.com/site/about/guidelines.xhtml

Supplementary Table 4: Full regression models predicting violence experiences

Variables	Bullying		Physical Attacks	
	Model 2 Adjusted	Model 3 Adjusted & interaction	Model 2 Adjusted	Model 3 Adjusted & interaction
Interview 2 vs interview 1	-0.42[-0.75,-0.08]	-0.44 [-0.78,-0.11]	-0.10[-0.27,0.07]	-0.09[-0.26,0.08]
Marital status (vs other)				
Single	-0.20[-1.03,0.63]	-0.24[-1.07, 0.59]	0.03[-0.40,0.46]	0.05[-0.38,0.48]
Ever worked	0.01[-0.27,0.28]	0.01[-0.27,0.28]	0.05[-0.09,0.20]	0.05[-0.09,0.19]
Number of people in the household	-0.04 [-0.07,-0.02]	-0.04 [-0.07,-0.02]	-0.01[-0.02,0.00]	-0.01[-0.02,0.00]
Mother is alive	0.74[-1.34, 2.82]	0.77[-1.31,2.85]	-0.30[-1.38,0.77]	-0.32[-1.40,0.76]
Father is alive	1.12[-2.41,0.17]	-1.11[-2.41, 0.18]	-0.35[-0.99,0.28]	-0.34[-0.98,0.29]
Lives with Mother	-0.12[-0.74,0.51]	-0.15[-0.77,0.48]	0.06[-0.26,0.39]	0.08[-0.25,0.40]
Lives with father	0.46[-0.18,1.10]	0.45[-0.18,1.09]	-0.13[-0.46,0.20]	-0.12[-0.45,0.21]
Lives alone	-0.17[-1.03,0.69]	-0.14[-1.00,0.72]	-0.38[-0.83,0.07]	-0.40[-0.84,0.05]
Parental support	-0.18 [-0.31,-0.05]	-0.18 [-0.31,-0.05]	-0.04[-0.11,0.03]	-0.04[-0.11,0.03]
Household wealth quintile				
Lowest (base)				
2	0.08[-0.34,0.50-]	0.06[-0.36,0.49]	-0.05[-0.27,0.17]	-0.04[-0.26,0.17]
3	-0.01[-0.44,0.42]	-0.02[-0.44,0.41]	-0.06[-0.28,0.16]	-0.06[-0.28,0.16]
4	-0.04[-0.52,0.44]	-0.07[-0.55,0.41]	-0.08[-0.32,0.17]	-0.06[-0.31,0.18]
Highest	0.43[-0.15,1.00]	0.40[-0.18,0.98]	0.12[-0.18,0.41]	0.13[-0.17,0.42]
Has own bedroom	-0.08[0.47,0.32]	-0.08[-0.47,0.32]	-0.01[-0.21,0.20]	-0.01[-0.21,0.20]
Radio hours/day	0.13[-0.01,0.27]	0.13[-0.00,0.27]	-0.02[-0.09,0.05]	-0.02[-0.09,0.05]
Frequency of watching television				
Never (base)				
Rarely (some hours per month)	-0.34[0.72,0.03]	-0.29[-0.67,0.09]	0.06[-0.13,0.26]	0.03[-0.16,0.23]
Often (several hours per week)	-0.11[-0.46,0.24]	-0.07[-0.42,0.29]	-0.04[-0.22,0.14]	-0.06-0.24,0.12]
Very often (several hours per day)	0.63 [0.14,1.11]	0.63 [0.15,1.12]	-0.19[00.44,0.06]	-0.19[-0.44,0.06]
Frequency of reading magazines				

For peer review only - http://bmjopen.bmj.com/site/about/guidelines.xhtml

Never (base)				
Rarely (some hours per month)	1.01 [0.46,1.57]	0.99 [0.43,1.54]	-0.18[-0.47,0.11]	-0.17[-0.46,0.12]
Often (several hours per week)	1.12 [0.38,1.85]	1.08 [0.35,1.81]	-0.22[-0.61,0.16]	-0.21[-0.59,0.17]
Very often (several hours per day)	-0.17[-1.06,0.73]	-0.07[-0.97,0.83]	0.90 [0.44,1.36]	0.85 [0.38,1.31]
Household size missing	-1.77[-3.73, 0.20]	-1.68[-3.64, 0.29]	-0.46[-1.39,0.46]	-0.50[-1.43,0.43]
Freq. of reading magazine missing	6.99 [4.23, 9.75]	6.93 [4.16,9.69]	0.46[-0.97,1.89]	0.49[-0.94,1.92]

All models present coefficients and 95% confidence intervals. All models contain fixed effects for each respondent. Age centred at 15.

For peer review only

STROBE Statement—Checklist of items that should be included in reports of ***cross-sectional studies***

	Item No	Recommendation	Reported on page no.
**Title and abstract**	1	(*a*) Indicate the study's design with a commonly used term in the title or the abstract	1 – 2
		(*b*) Provide in the abstract an informative and balanced summary of what was done and what was found	1 – 2
**Introduction**			
Background/rationale	2	Explain the scientific background and rationale for the investigation being reported	2 – 5
Objectives	3	State specific objectives, including any prespecified hypotheses	5 – 6
**Methods**			
Study design	4	Present key elements of study design early in the paper	6
Setting	5	Describe the setting, locations, and relevant dates, including periods of recruitment, exposure, follow-up, and data collection	6 – 7
Participants	6	(*a*) Give the eligibility criteria, and the sources and methods of selection of participants	6
Variables	7	Clearly define all outcomes, exposures, predictors, potential confounders, and effect modifiers. Give diagnostic criteria, if applicable	7
Data sources/ measurement	8*	For each variable of interest, give sources of data and details of methods of assessment (measurement). Describe comparability of assessment methods if there is more than one group	6 -7
Bias	9	Describe any efforts to address potential sources of bias	8 - 9
Study size	10	Explain how the study size was arrived at	6
Quantitative variables	11	Explain how quantitative variables were handled in the analyses. If applicable, describe which groupings were chosen and why	8 – 9
Statistical methods	12	(*a*) Describe all statistical methods, including those used to control for confounding	8 – 9
		(*b*) Describe any methods used to examine subgroups and interactions	N/A
		© Explain how missing data were addressed	N/A
		(*d*) If applicable, describe analytical methods taking account of sampling strategy	N/A
		€ Describe any sensitivity analyses	N/A
**Results**			
Participants	13*	(a) Report numbers of individuals at each stage of study—eg numbers potentially eligible, examined for eligibility, confirmed eligible, included in the study, completing follow-up, and analysed	See page 4 of supplementary material
		(b) Give reasons for non-participation at each stage	
		(c) Consider use of a flow diagram	
Descriptive data	14*	(a) Give characteristics of study participants (eg demographic,	10

For peer review only - http://bmjopen.bmj.com/site/about/guidelines.xhtml

		clinical, social) and information on exposures and potential confounders	
		(b) Indicate number of participants with missing data for each variable of interest	See page 4 of supplementary material
Outcome data	15*	Report numbers of outcome events or summary measures	10
Main results	16	(*a*) Give unadjusted estimates and, if applicable, confounder-adjusted estimates and their precision (eg, 95% confidence interval). Make clear which confounders were adjusted for and why they were included	12
		(*b*) Report category boundaries when continuous variables were categorized	N/A
		© If relevant, consider translating estimates of relative risk into absolute risk for a meaningful time period	N/A
Other analyses	17	Report other analyses done—eg analyses of subgroups and interactions, and sensitivity analyses	N/A
**Discussion**			
Key results	18	Summarise key results with reference to study objectives	17
Limitations	19	Discuss limitations of the study, taking into account sources of potential bias or imprecision. Discuss both direction and magnitude of any potential bias	16
Interpretation	20	Give a cautious overall interpretation of results considering objectives, limitations, multiplicity of analyses, results from similar studies, and other relevant evidence	16 - 17
Generalisability	21	Discuss the generalisability (external validity) of the study results	17
**Other information**			
Funding	22	Give the source of funding and the role of the funders for the present study and, if applicable, for the original study on which the present article is based	17

*Give information separately for exposed and unexposed groups.

Note: An Explanation and Elaboration article discusses each checklist item and gives methodological background and published examples of transparent reporting. The STROBE checklist is best used in conjunction with this article (freely available on the Web sites of PLoS Medicine at http://www.plosmedicine.org/, Annals of Internal Medicine at http://www.annals.org/, and Epidemiology at http://www.epidem.com/). Information on the STROBE Initiative is available at www.strobe-statement.org.

For peer review only
